# Supplementary figures and images for: Systematic analysis of the molecular and biophysical properties of key DNA damage response factors
Source: eLife. 2023 Jun 21;12:e87086. doi: 10.7554/eLife.87086 (PMC10319438; doi:10.7554/eLife.87086)

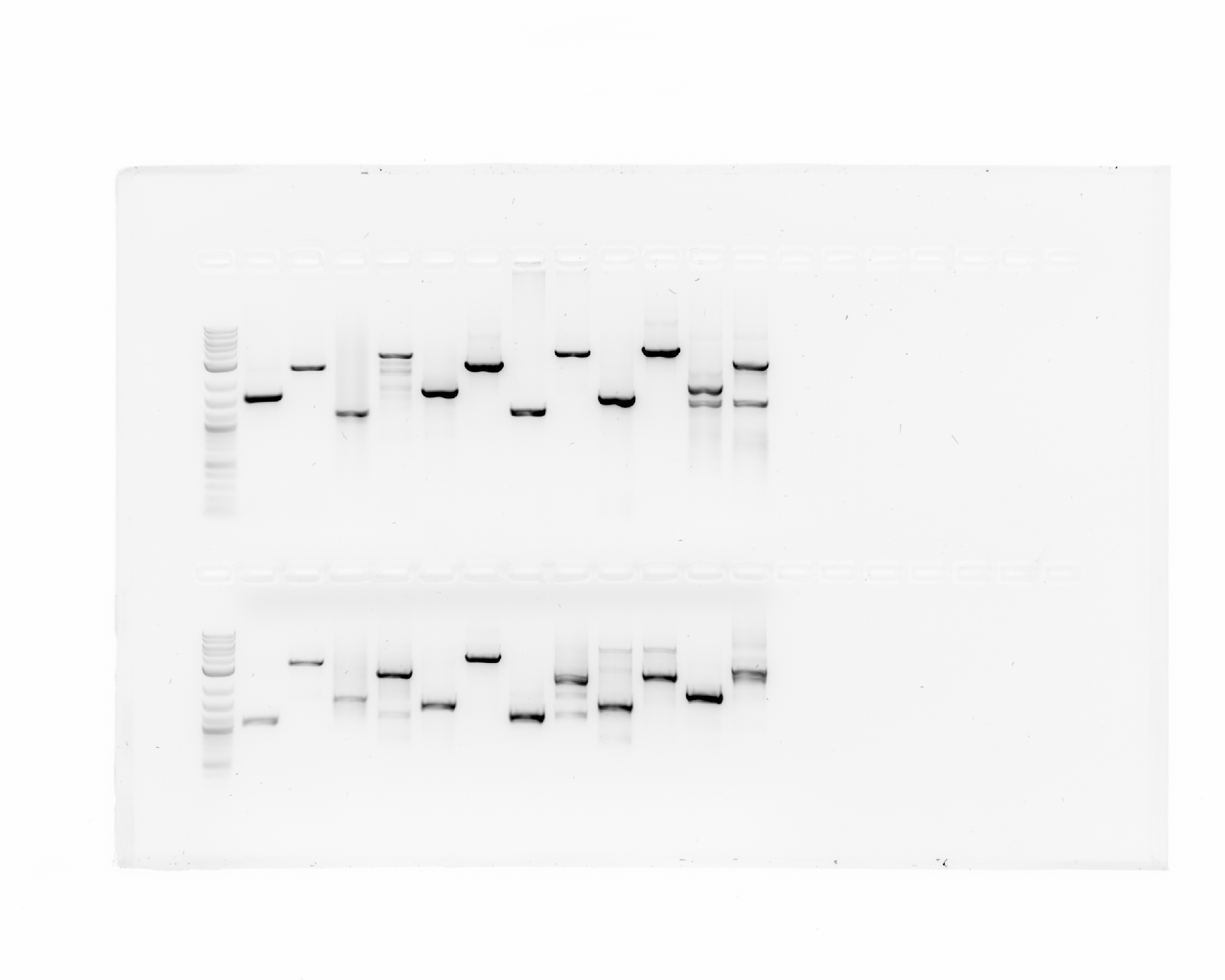

Supplement: Figure 1—source data 1. [file elife-87086-fig1-data1.zip › Figure 1-Source Data 1/Figure 1B/Agarose Gel genomic PCR for Primary HaloTag Clones.tif]

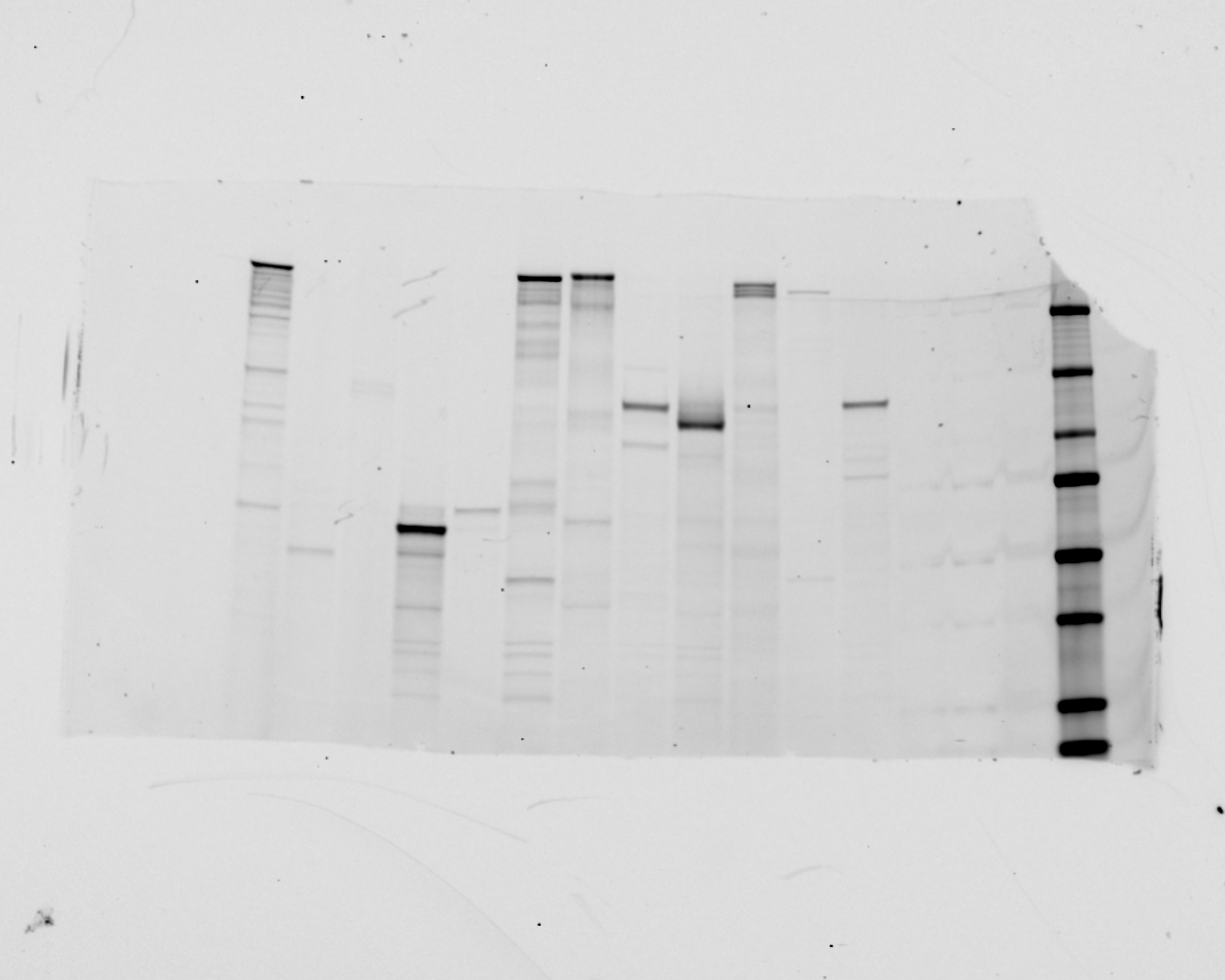

Supplement: Figure 1—source data 1. [file elife-87086-fig1-data1.zip › Figure 1-Source Data 1/Figure 1C/JF646 Fluorescent Gel for HaloTag Proteins.tif]

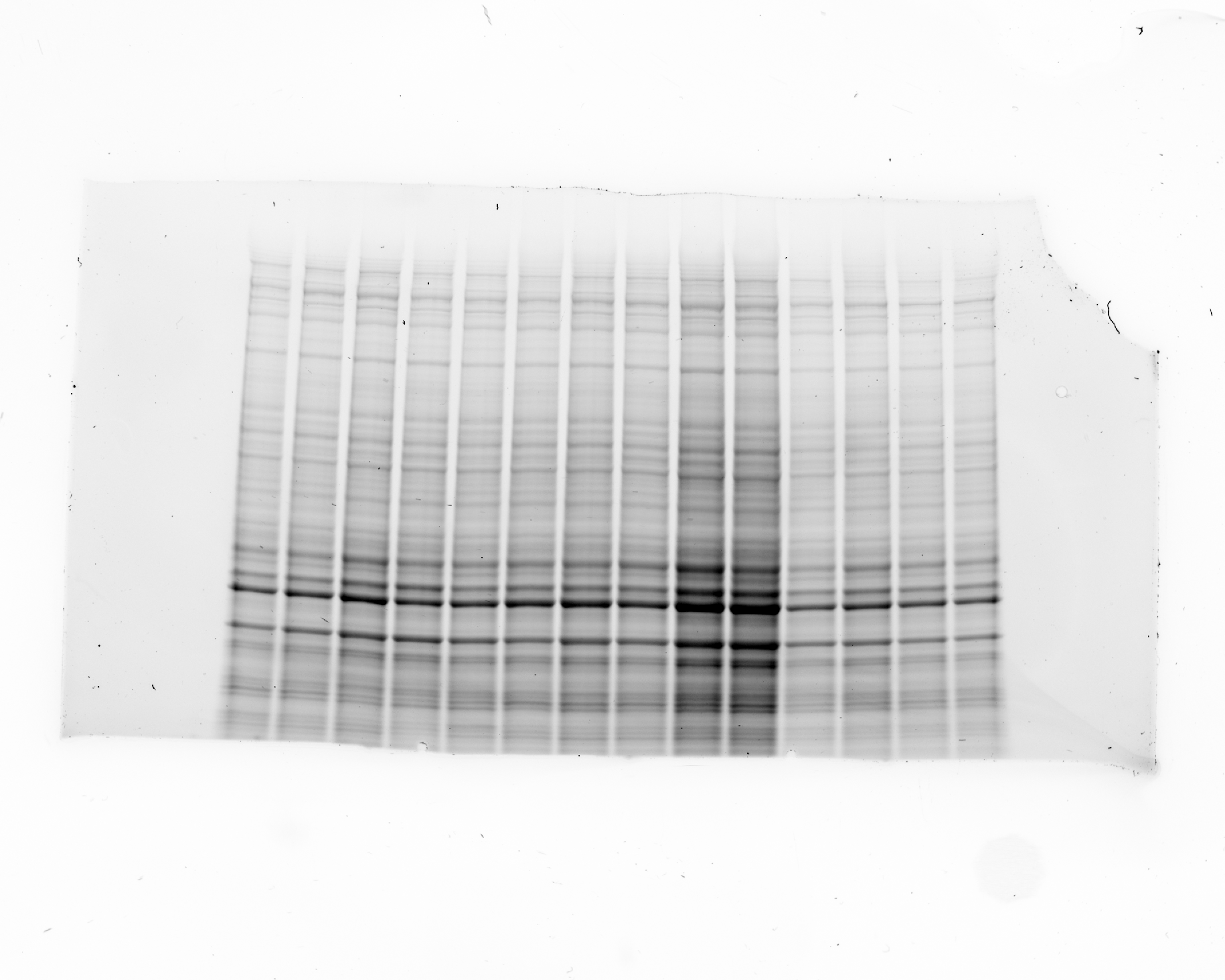

Supplement: Figure 1—source data 1. [file elife-87086-fig1-data1.zip › Figure 1-Source Data 1/Figure 1C/Stain-Free Loading Control.tif]

Figure 1-source data 1

Figure 1B

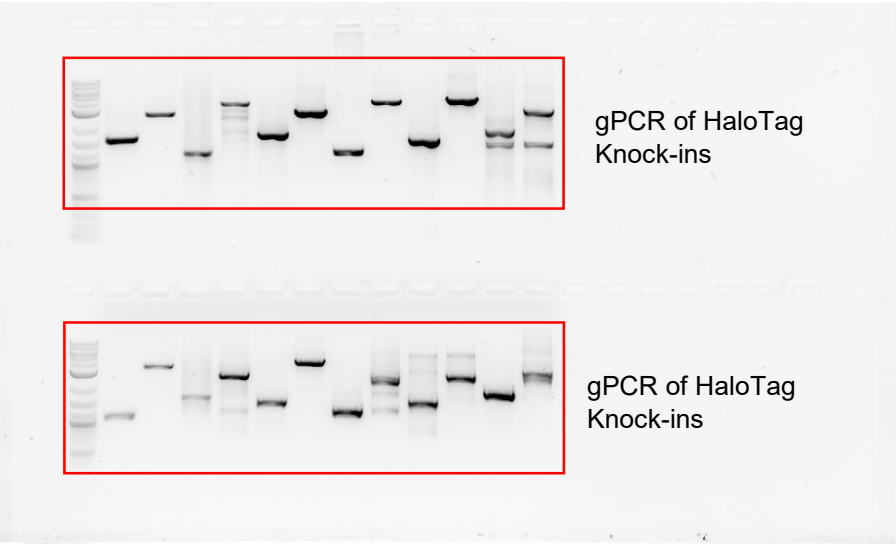

Figure 1C

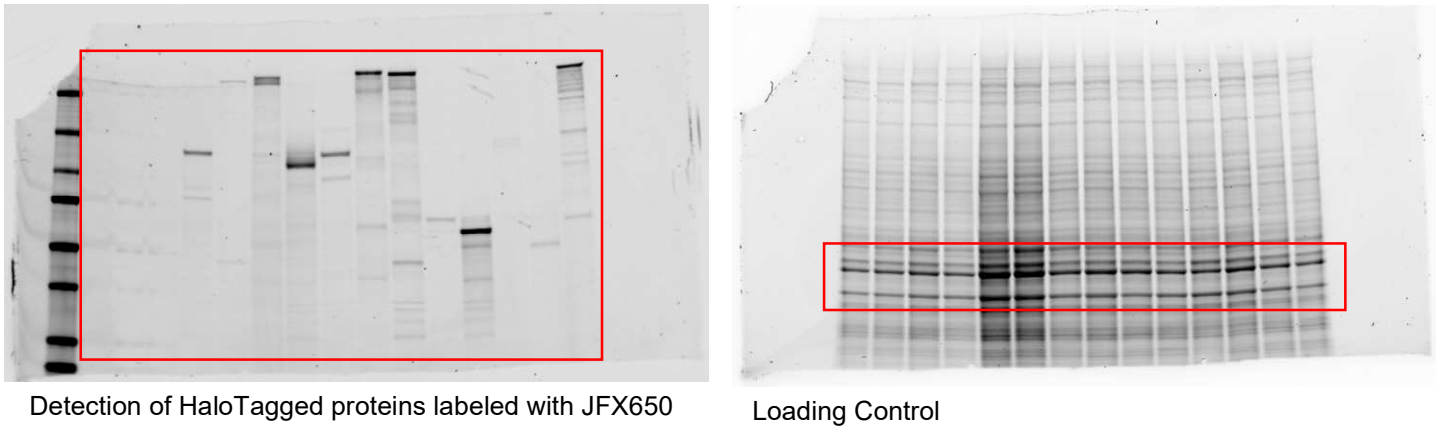

Supplement: Figure 1—source data 1. [file elife-87086-fig1-data1.zip › Figure 1-Source Data 1/Figure 1-Source Data 1.pdf]

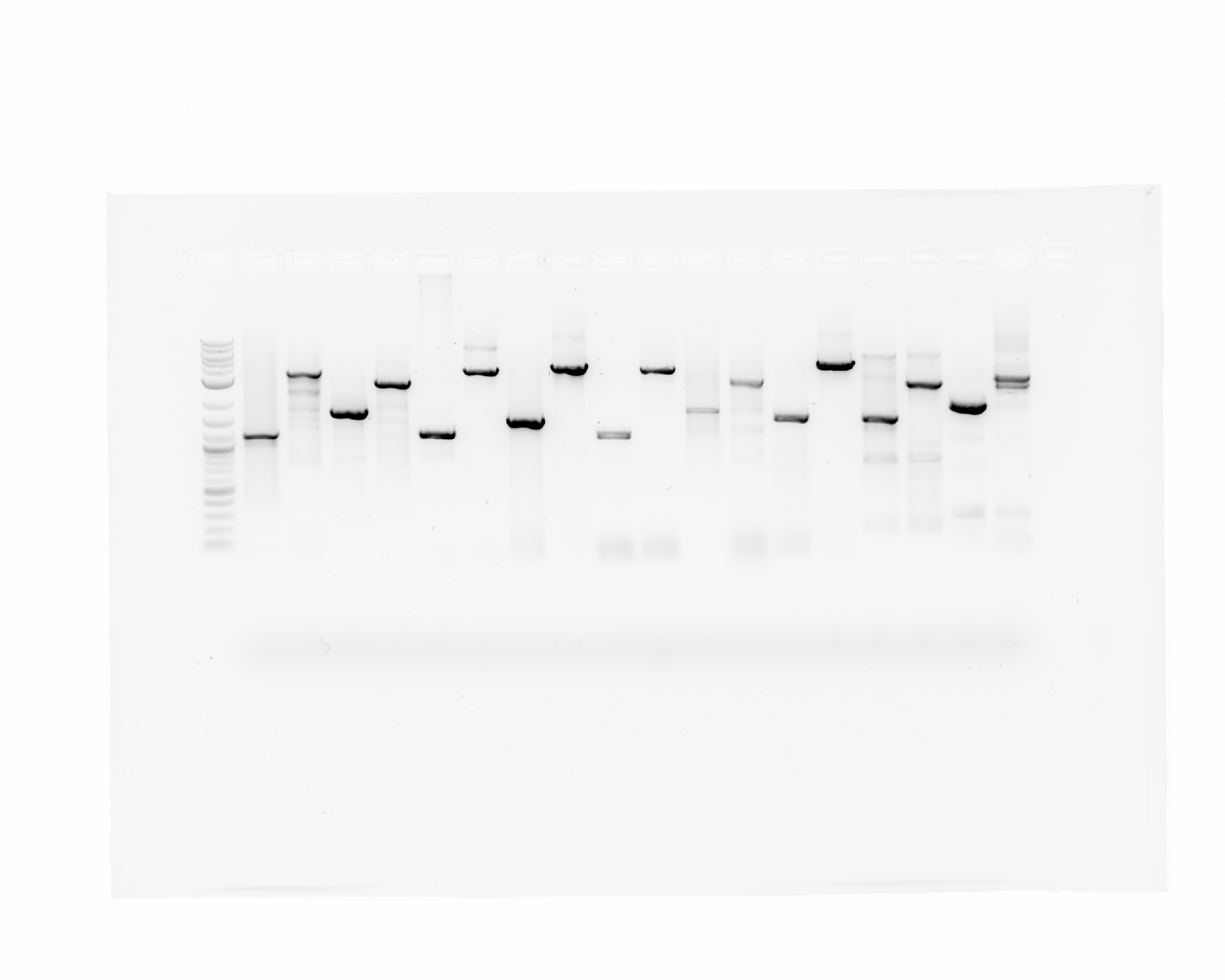

Supplement: Figure 1—figure supplement 1—source data 1. [file elife-87086-fig1-figsupp1-data1.zip › Figure 1-Figure Supplement 1-Source Data 1/Figure 1-figure supplement 1C/Agarose Gel of genomic PCR products for secondary HaloTag Clones.tif]

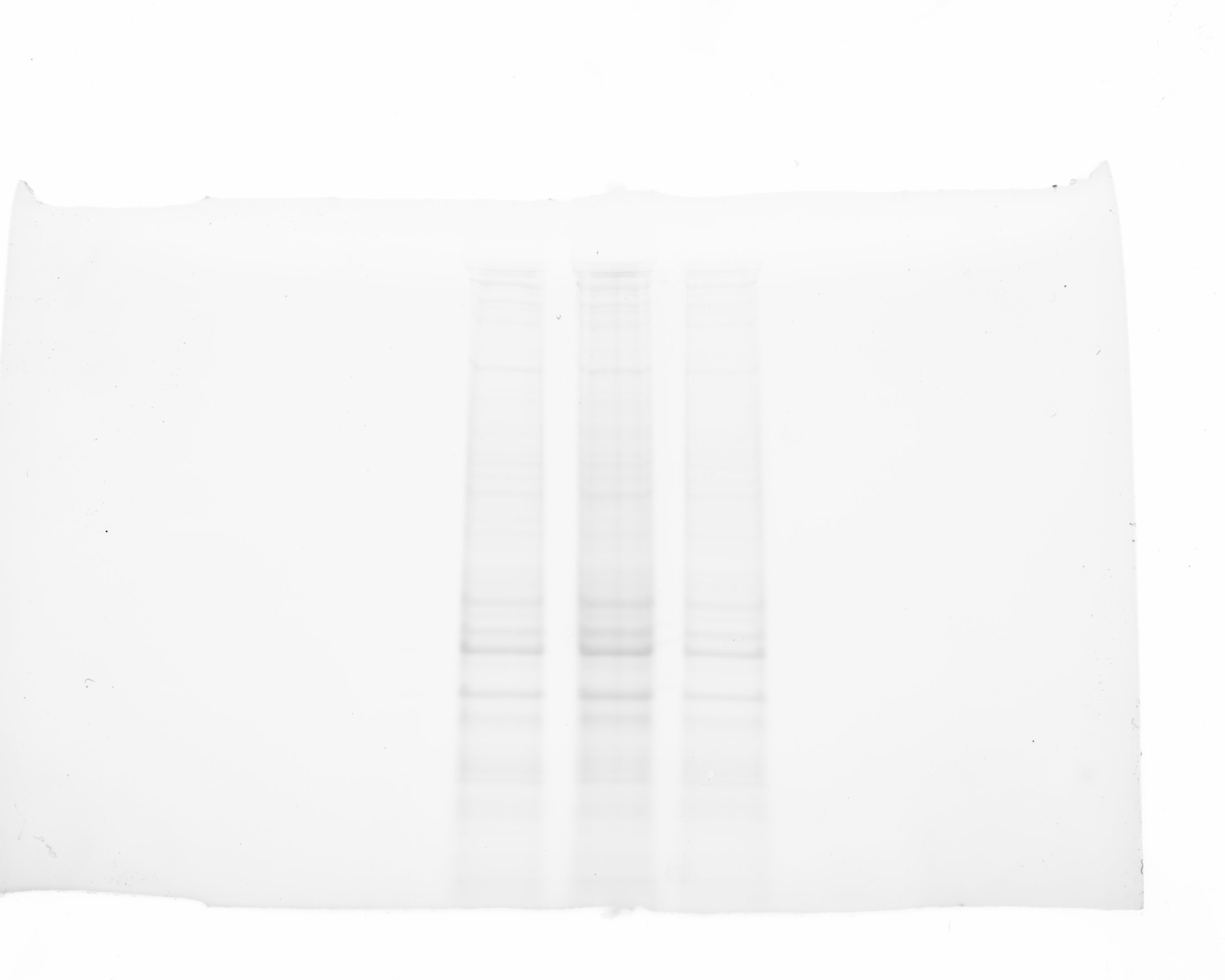

Supplement: Figure 1—figure supplement 1—source data 1. [file elife-87086-fig1-figsupp1-data1.zip › Figure 1-Figure Supplement 1-Source Data 1/Figure 1-figure supplement 1D/Supplemental Figure 1D Bottom Panel.tif]

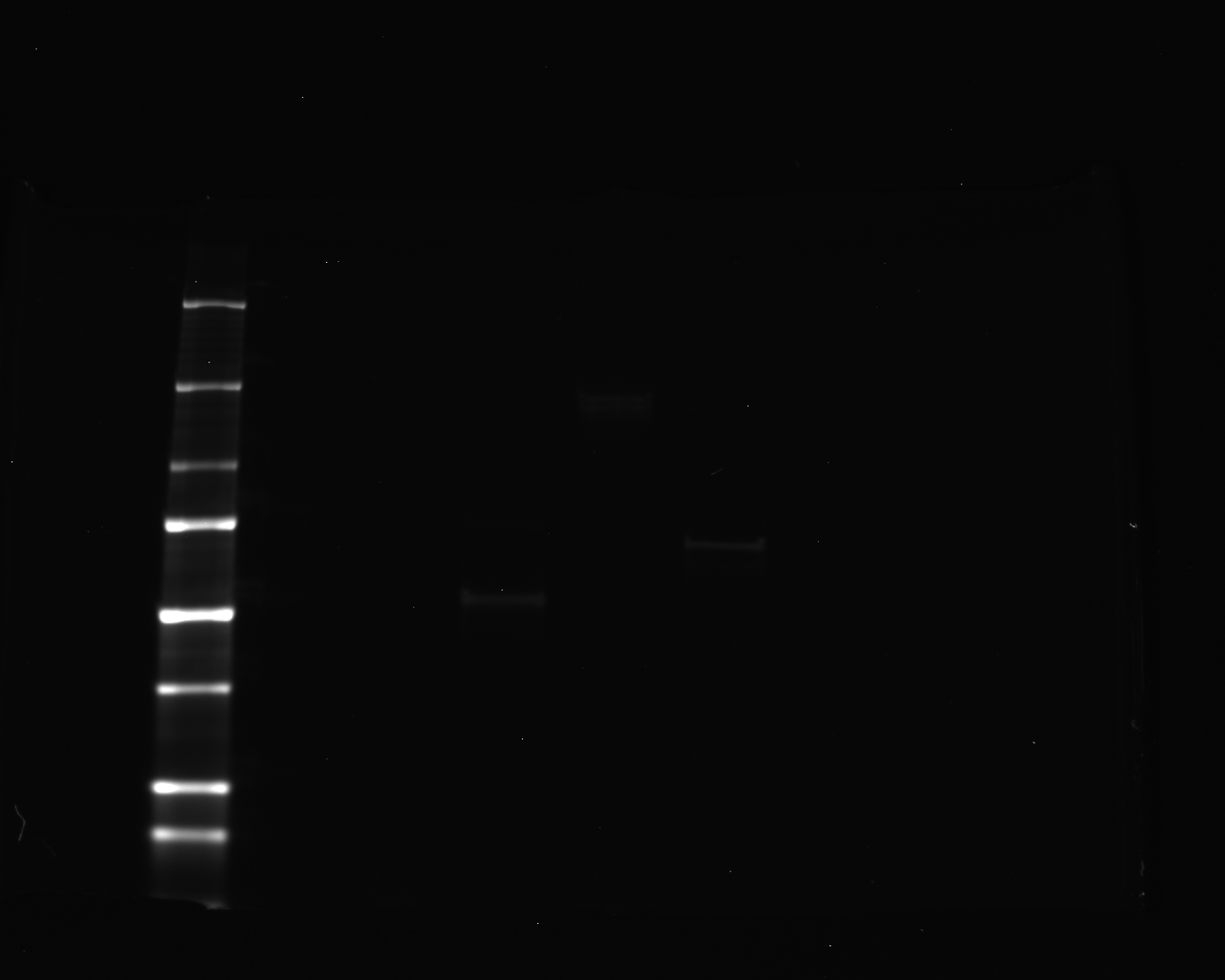

Supplement: Figure 1—figure supplement 1—source data 1. [file elife-87086-fig1-figsupp1-data1.zip › Figure 1-Figure Supplement 1-Source Data 1/Figure 1-figure supplement 1D/Supplemental Figure 1D Top Panel.tif]

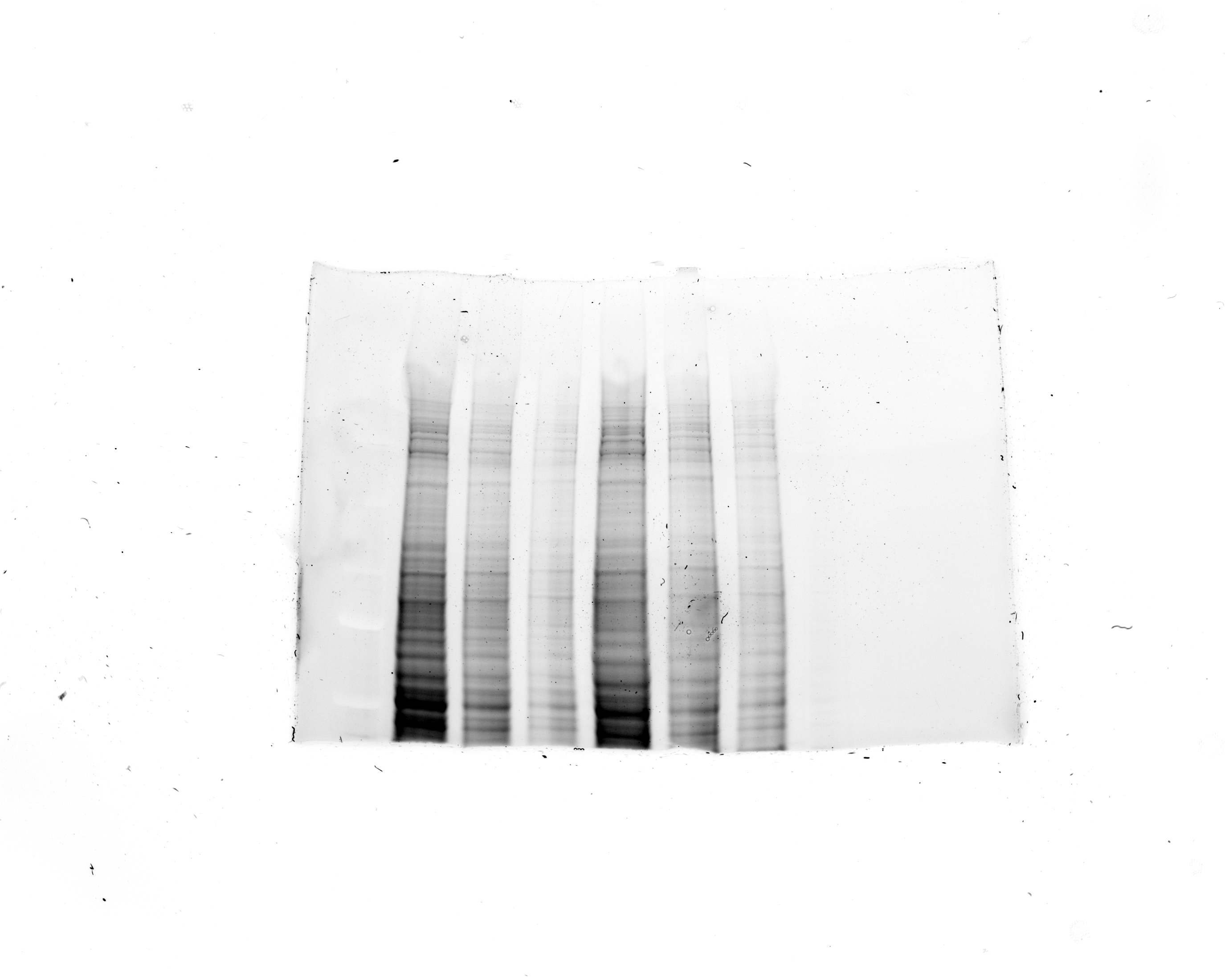

Supplement: Figure 1—figure supplement 1—source data 1. [file elife-87086-fig1-figsupp1-data1.zip › Figure 1-Figure Supplement 1-Source Data 1/Figure 1-figure supplement 1F/anti-53BP1 Stain-Free Loading Control.tif]

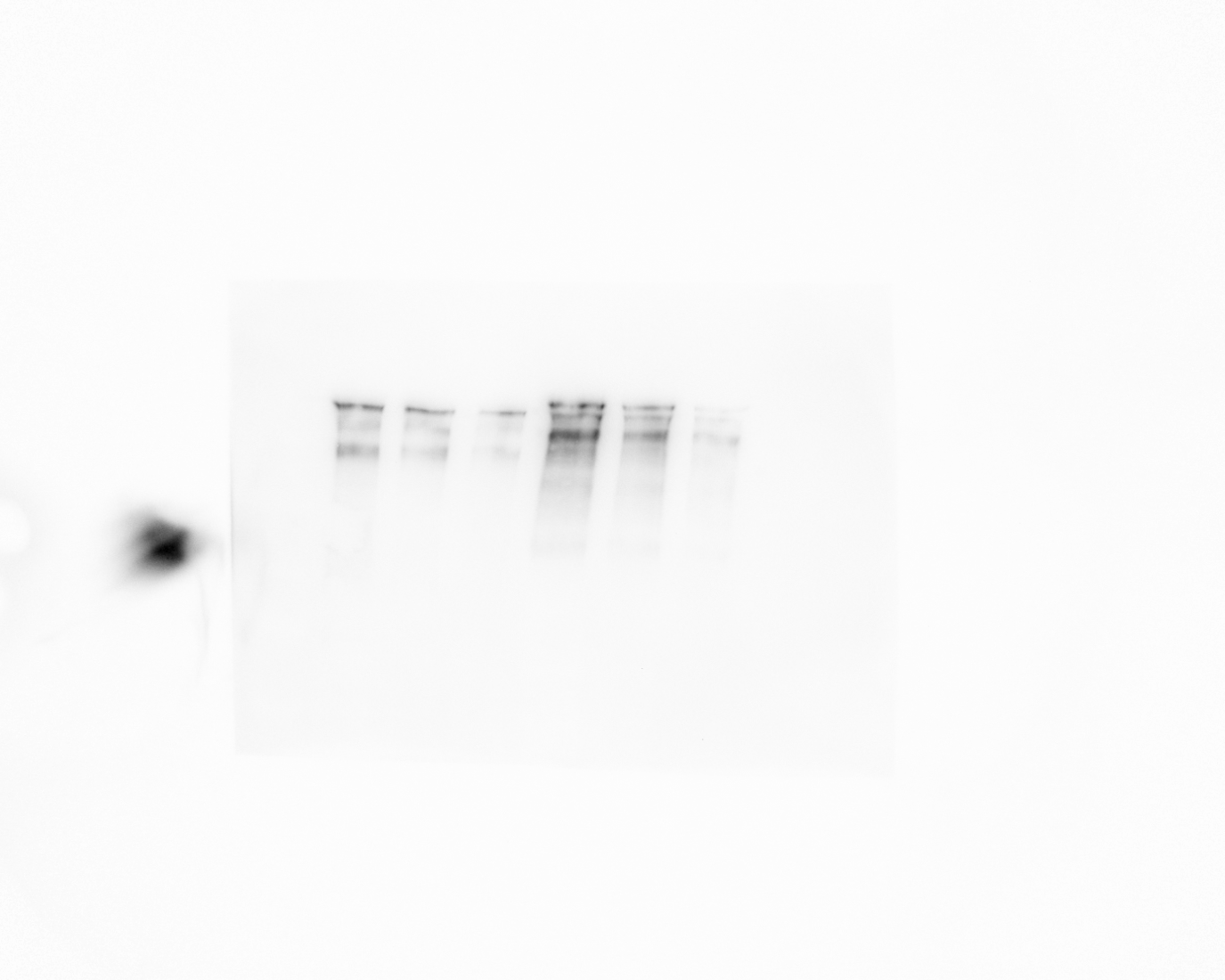

Supplement: Figure 1—figure supplement 1—source data 1. [file elife-87086-fig1-figsupp1-data1.zip › Figure 1-Figure Supplement 1-Source Data 1/Figure 1-figure supplement 1F/anti-53BP1 Western Blot.tif]

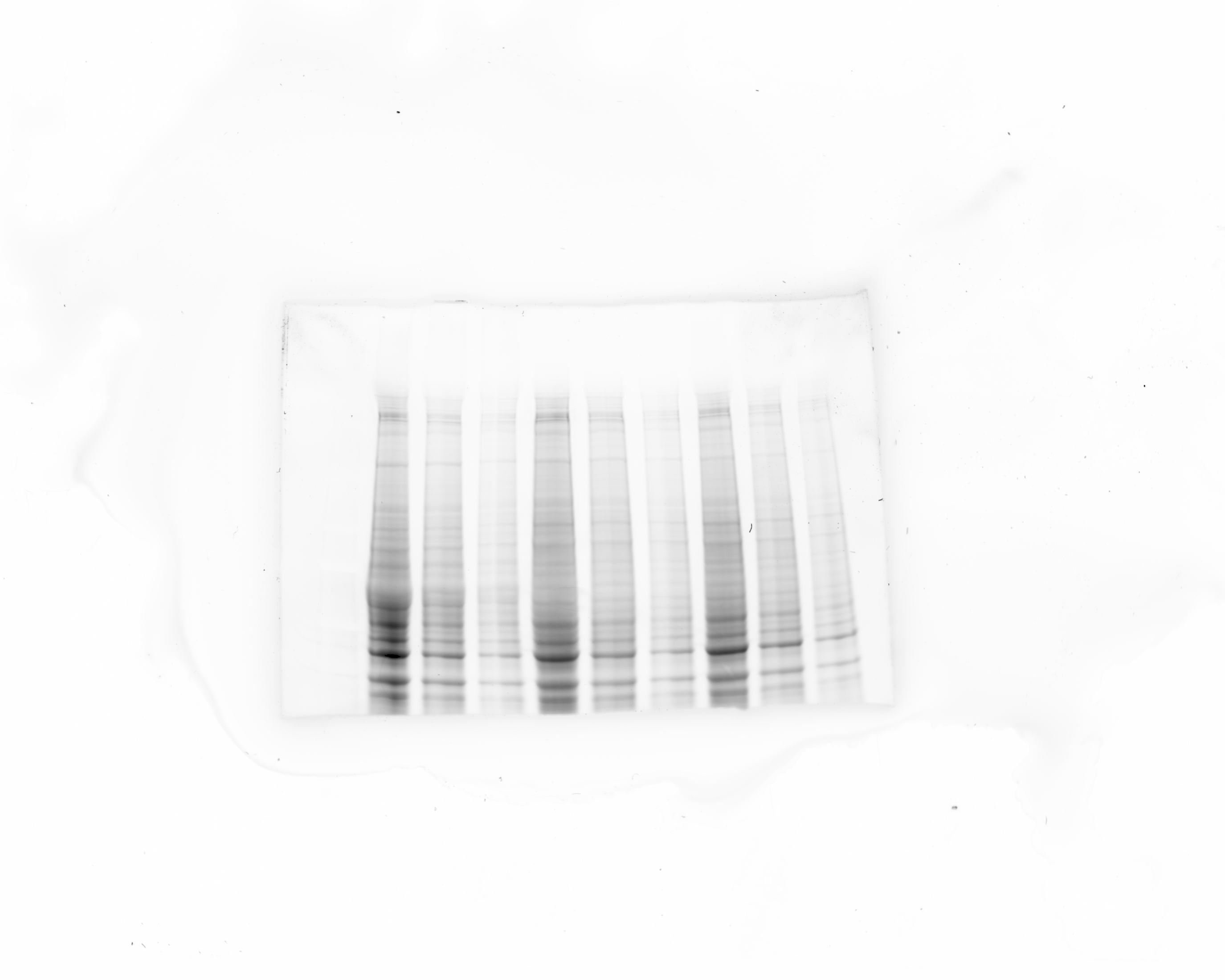

Supplement: Figure 1—figure supplement 1—source data 1. [file elife-87086-fig1-figsupp1-data1.zip › Figure 1-Figure Supplement 1-Source Data 1/Figure 1-figure supplement 1F/anti-ATM (C-Term Tag) Stain-Free Loading Control.tif]

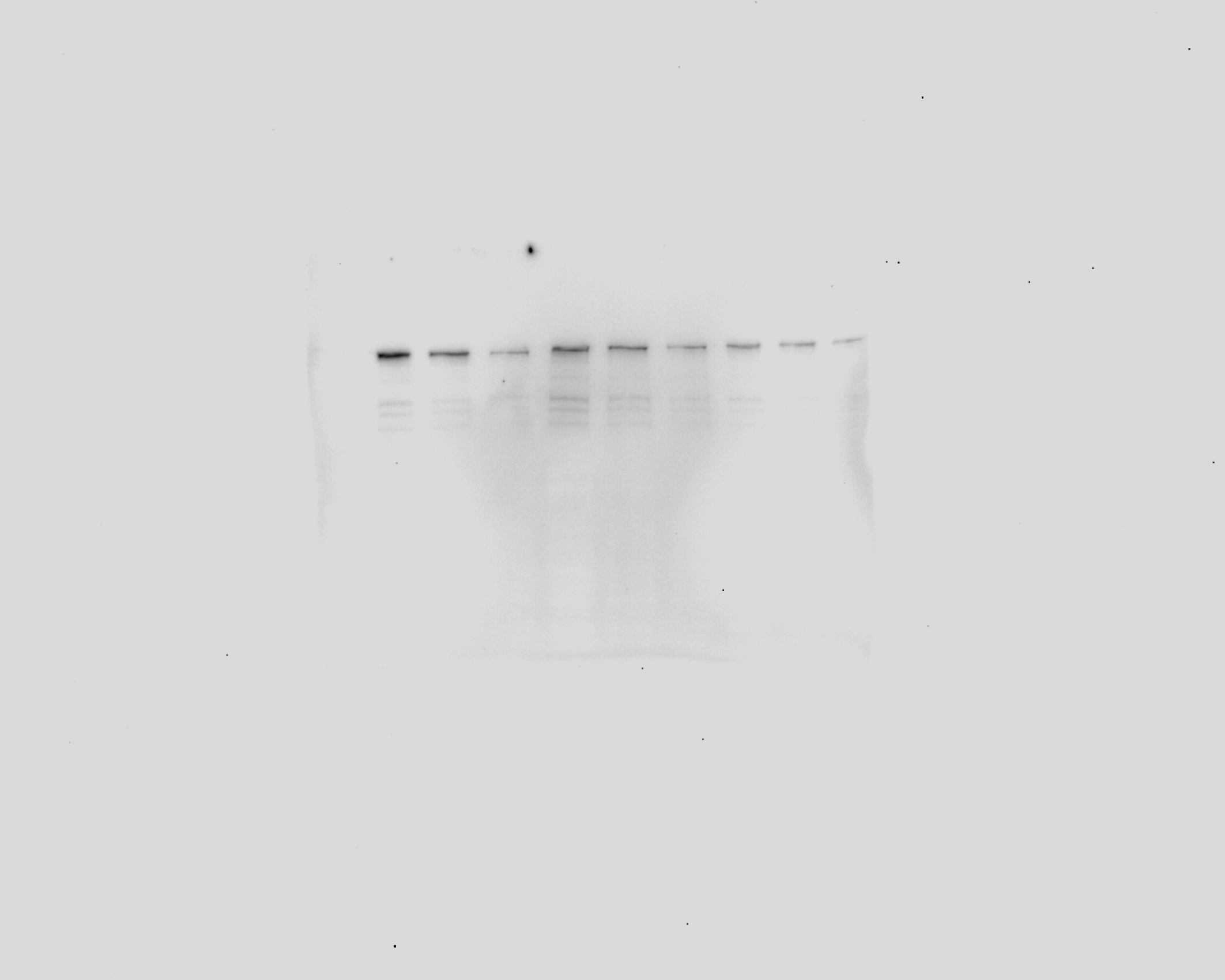

Supplement: Figure 1—figure supplement 1—source data 1. [file elife-87086-fig1-figsupp1-data1.zip › Figure 1-Figure Supplement 1-Source Data 1/Figure 1-figure supplement 1F/anti-ATM (C-Term Tag) Western Blot.tif]

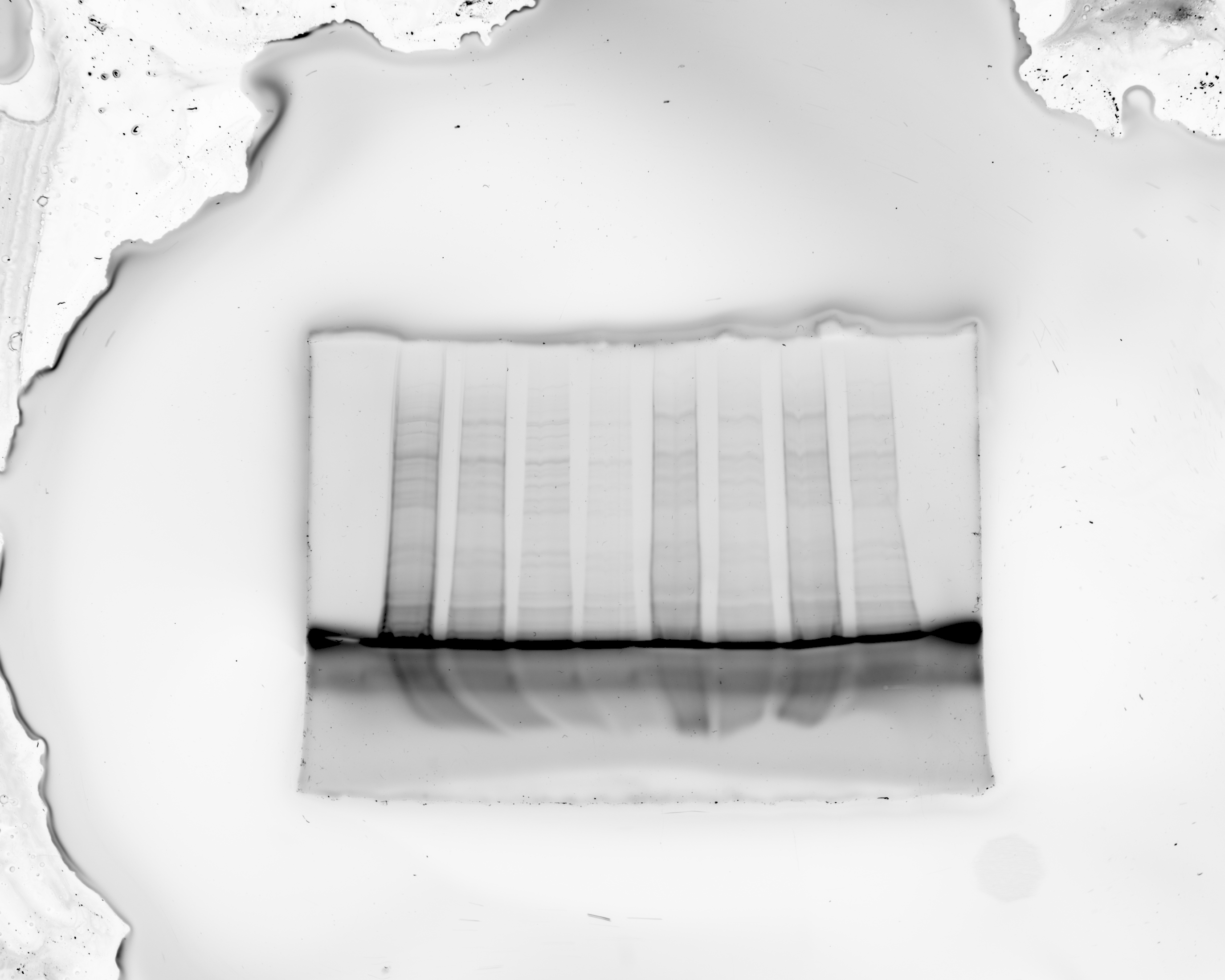

Supplement: Figure 1—figure supplement 1—source data 1. [file elife-87086-fig1-figsupp1-data1.zip › Figure 1-Figure Supplement 1-Source Data 1/Figure 1-figure supplement 1F/anti-DNA-PKcs Stain-Free Loading Control.tif]

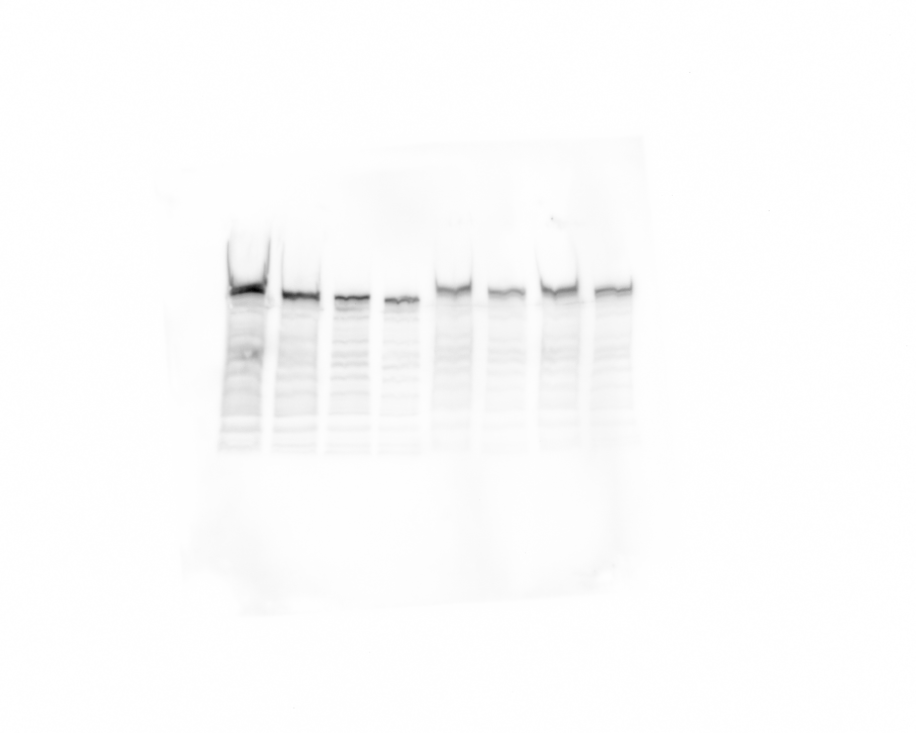

Supplement: Figure 1—figure supplement 1—source data 1. [file elife-87086-fig1-figsupp1-data1.zip › Figure 1-Figure Supplement 1-Source Data 1/Figure 1-figure supplement 1F/anti-DNA-PKcs Western Blot.tif]

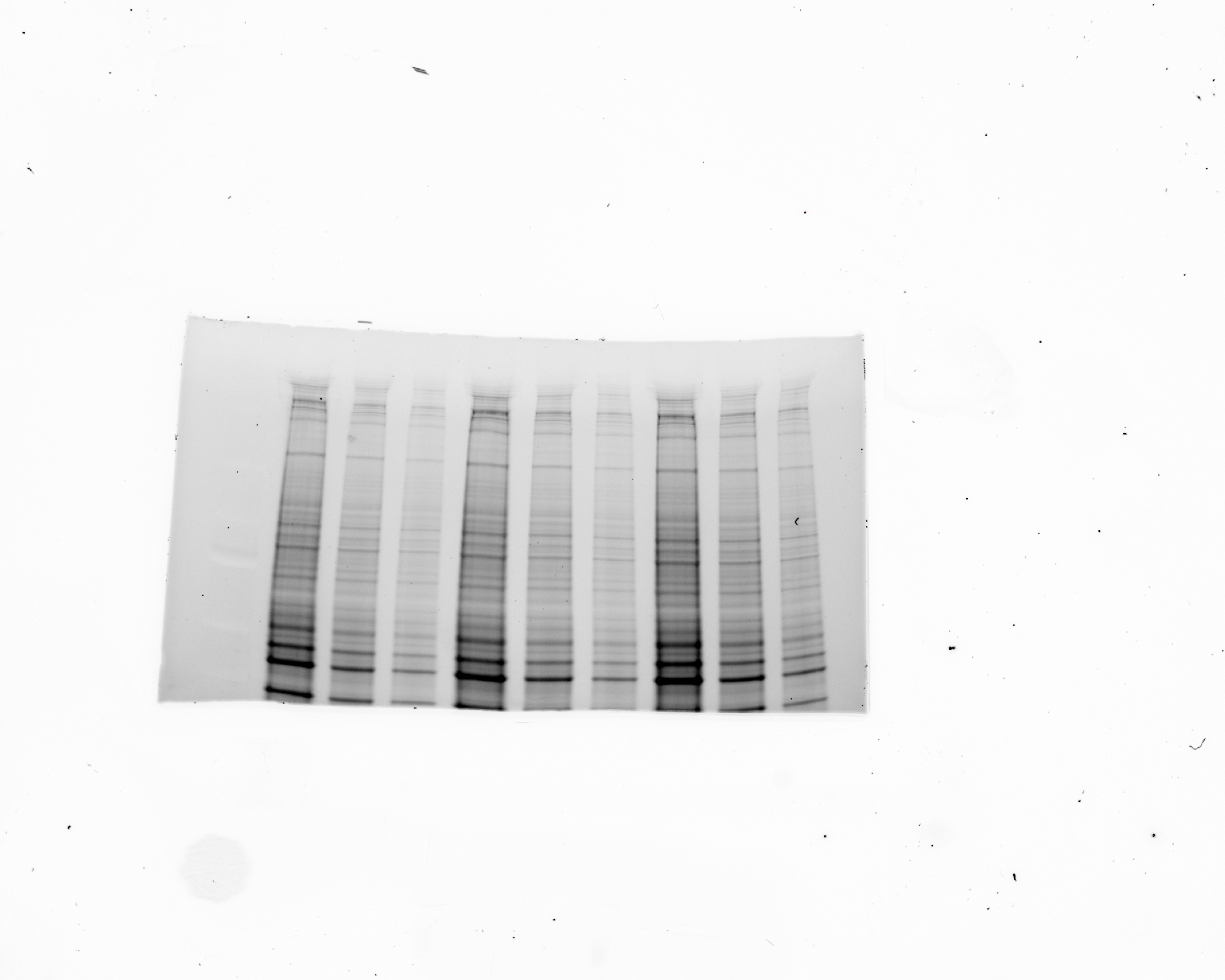

Supplement: Figure 1—figure supplement 1—source data 1. [file elife-87086-fig1-figsupp1-data1.zip › Figure 1-Figure Supplement 1-Source Data 1/Figure 1-figure supplement 1F/anti-FLAG (Halo-RNF169) Stain-Free Loading Control.tif]

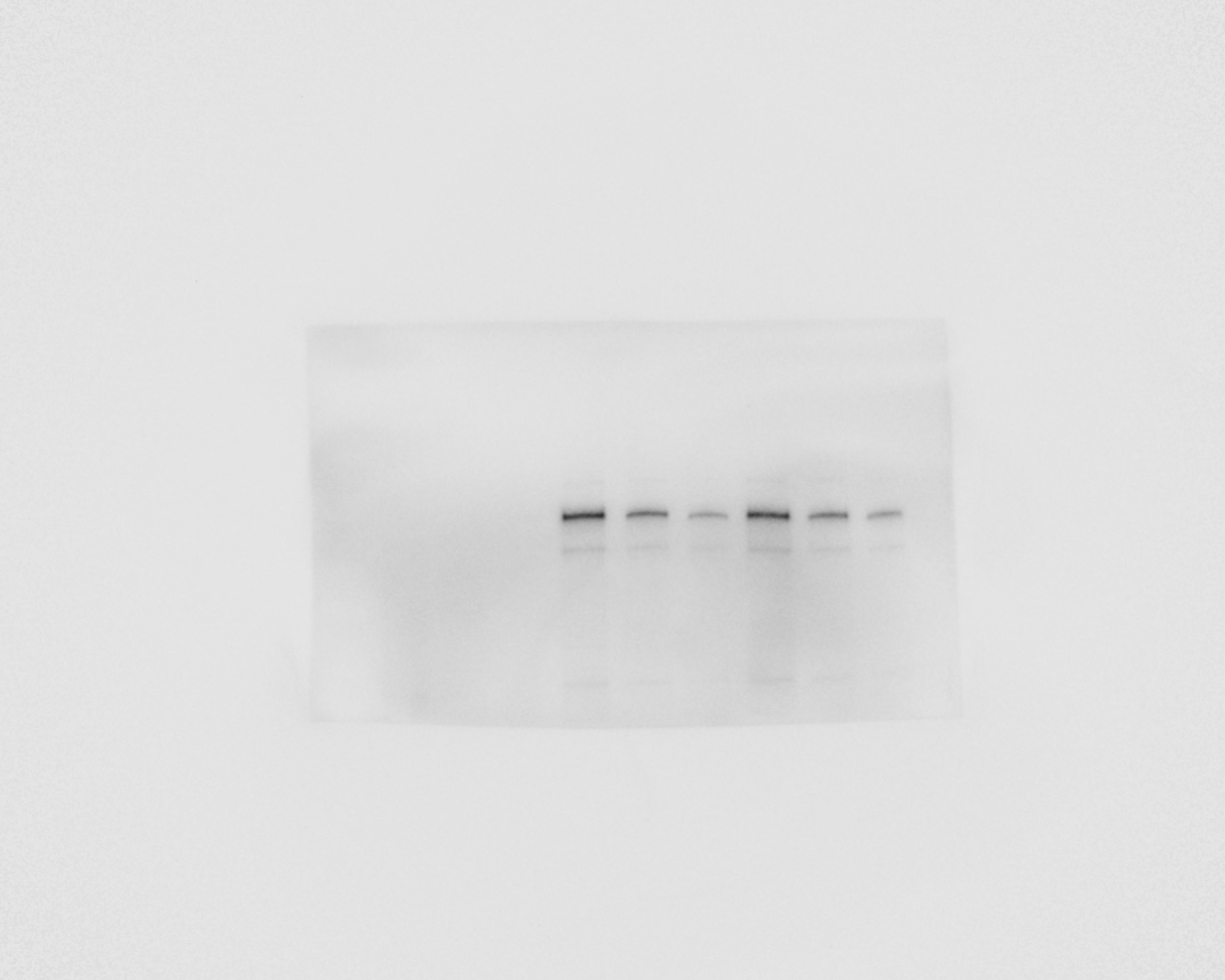

Supplement: Figure 1—figure supplement 1—source data 1. [file elife-87086-fig1-figsupp1-data1.zip › Figure 1-Figure Supplement 1-Source Data 1/Figure 1-figure supplement 1F/anti-FLAG (Halo-RNF169) Western Blot.tif]

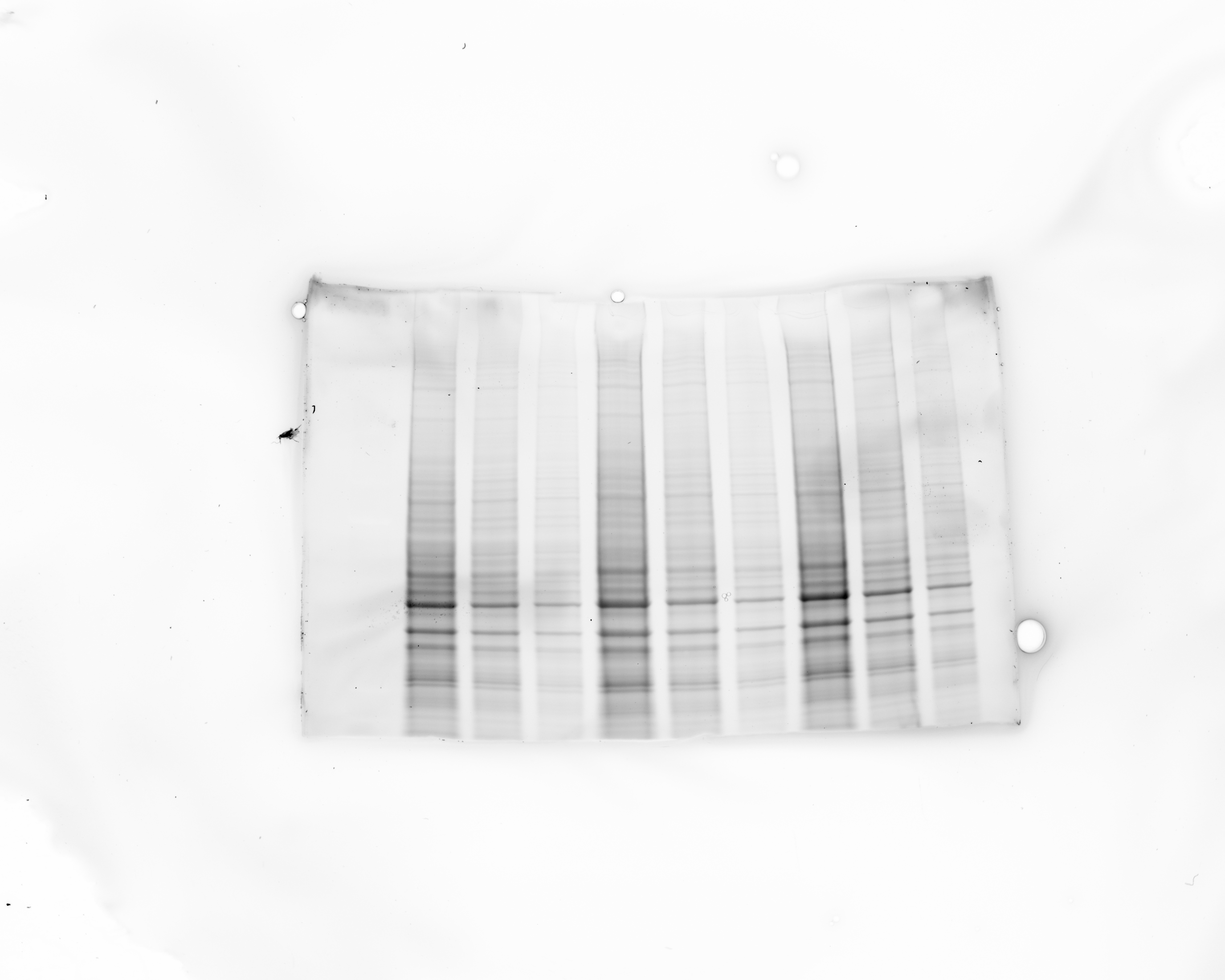

Supplement: Figure 1—figure supplement 1—source data 1. [file elife-87086-fig1-figsupp1-data1.zip › Figure 1-Figure Supplement 1-Source Data 1/Figure 1-figure supplement 1F/anti-FLAG (Halo-SHLD1) Stain-Free Loading Control.tif]

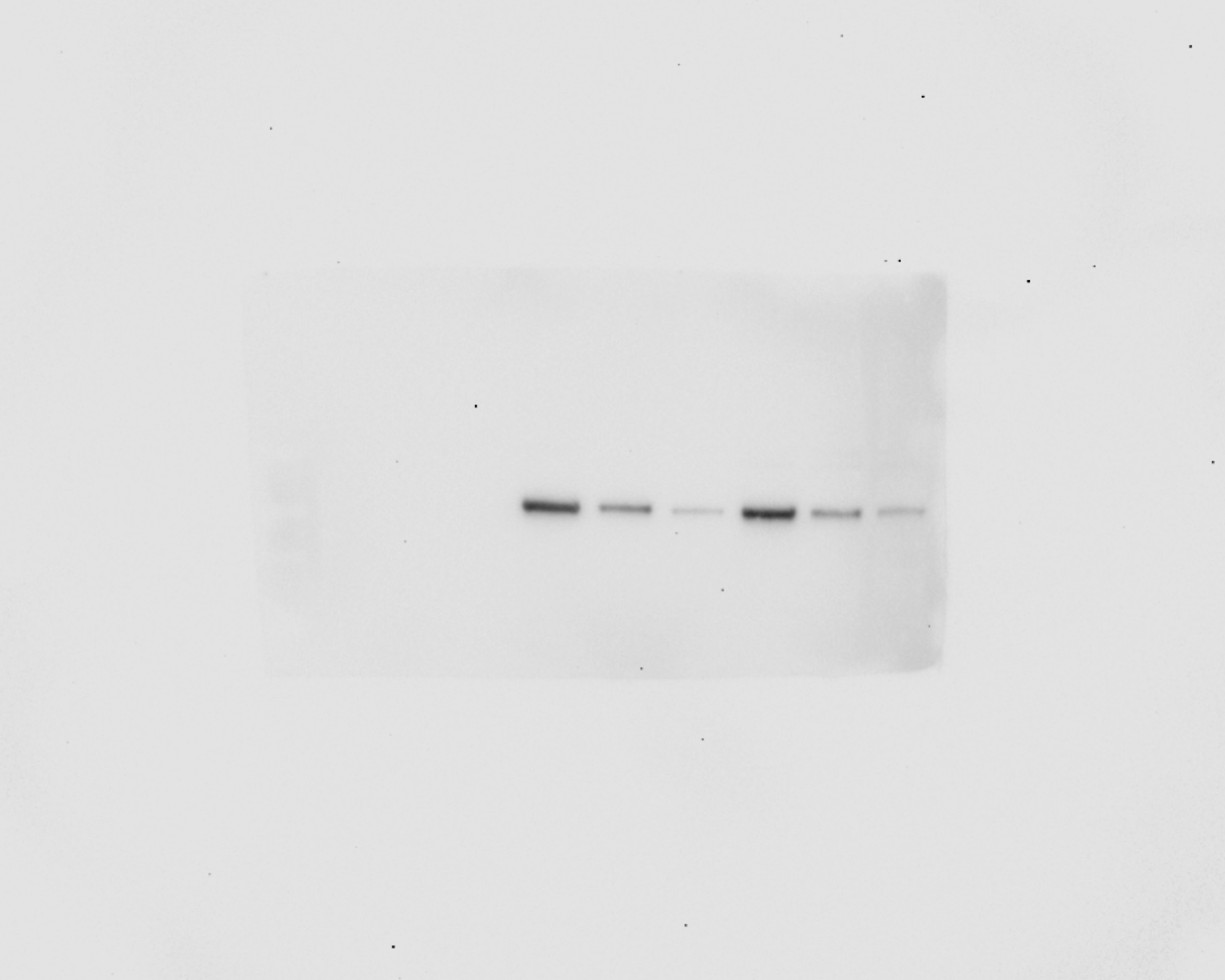

Supplement: Figure 1—figure supplement 1—source data 1. [file elife-87086-fig1-figsupp1-data1.zip › Figure 1-Figure Supplement 1-Source Data 1/Figure 1-figure supplement 1F/anti-FLAG (Halo-SHLD1) Western Blot.tif]

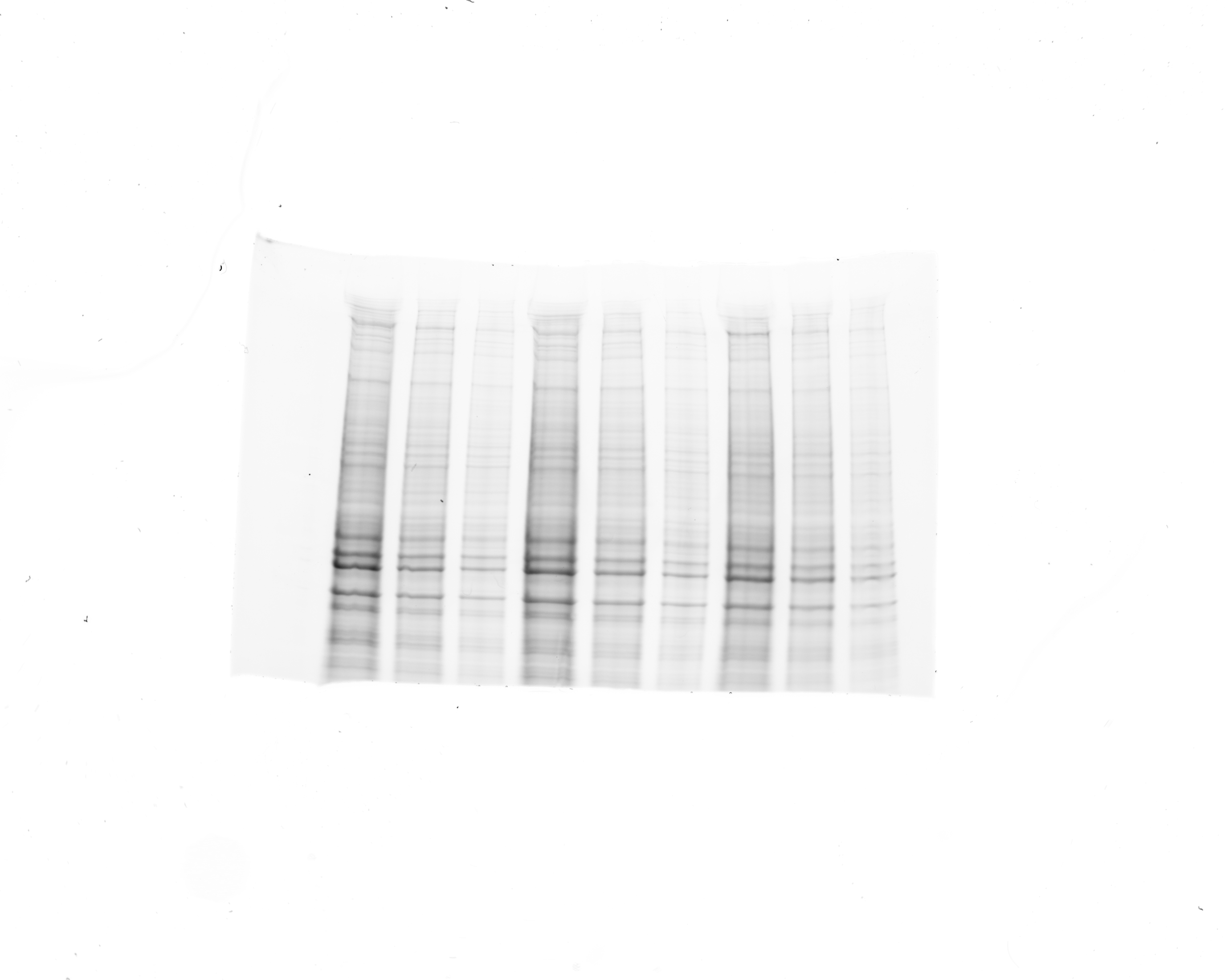

Supplement: Figure 1—figure supplement 1—source data 1. [file elife-87086-fig1-figsupp1-data1.zip › Figure 1-Figure Supplement 1-Source Data 1/Figure 1-figure supplement 1F/anti-FLAG (Halo-SHLD2) Stain-Free Loading Control.tif]

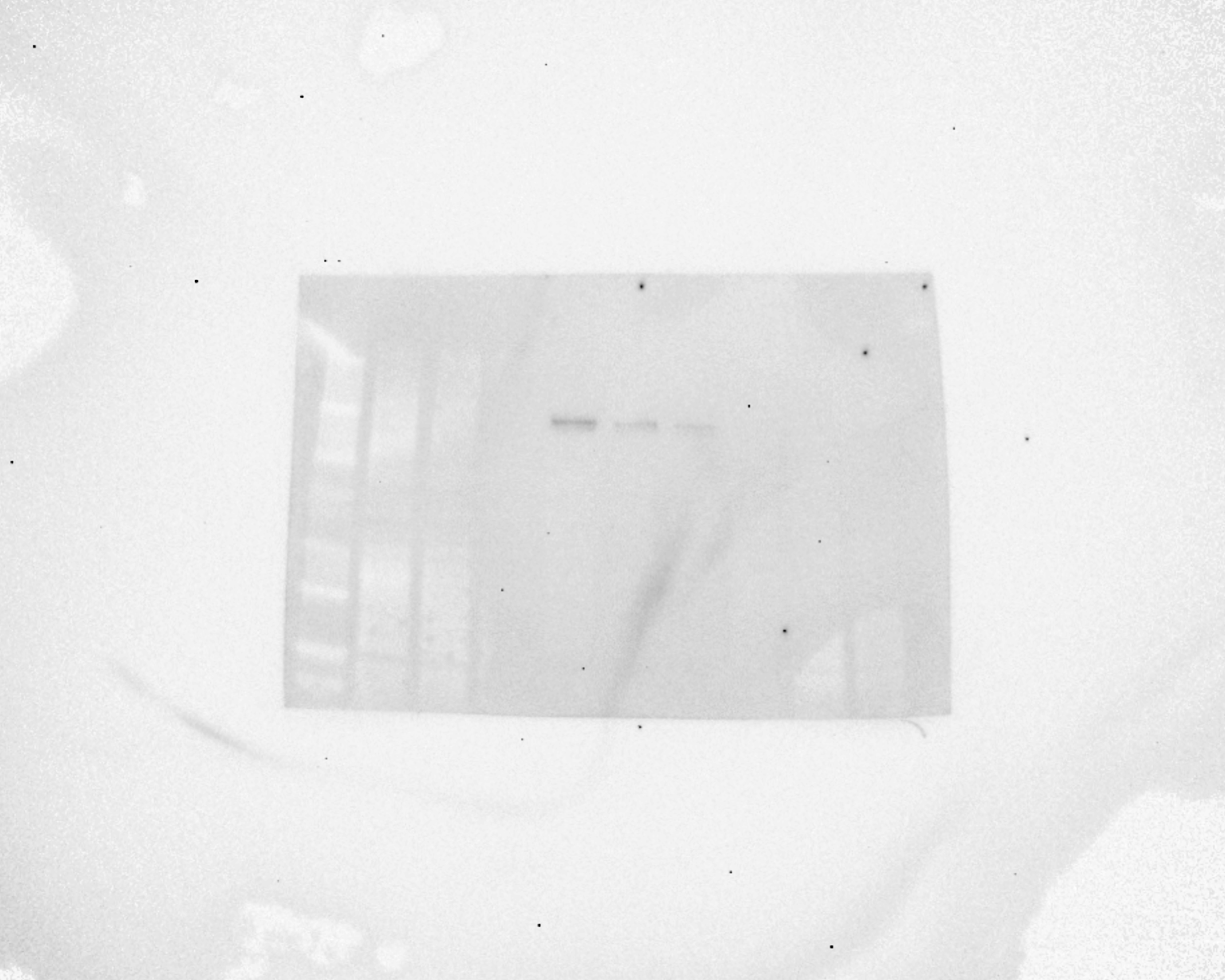

Supplement: Figure 1—figure supplement 1—source data 1. [file elife-87086-fig1-figsupp1-data1.zip › Figure 1-Figure Supplement 1-Source Data 1/Figure 1-figure supplement 1F/anti-FLAG (Halo-SHLD2) Western Blot.tif]

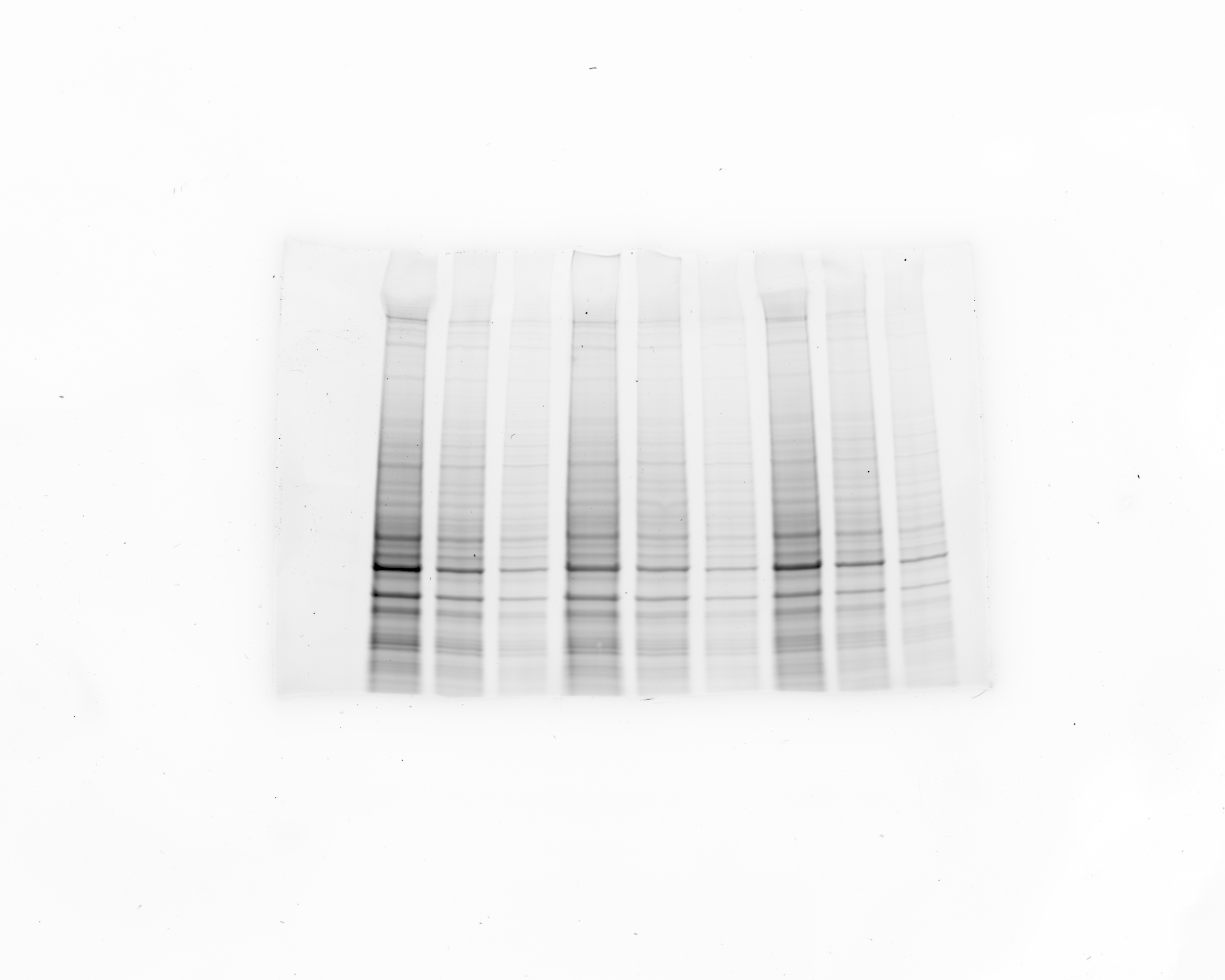

Supplement: Figure 1—figure supplement 1—source data 1. [file elife-87086-fig1-figsupp1-data1.zip › Figure 1-Figure Supplement 1-Source Data 1/Figure 1-figure supplement 1F/anti-FLAG (Halo-SHLD3) Stain-Free Loading Control.tif]

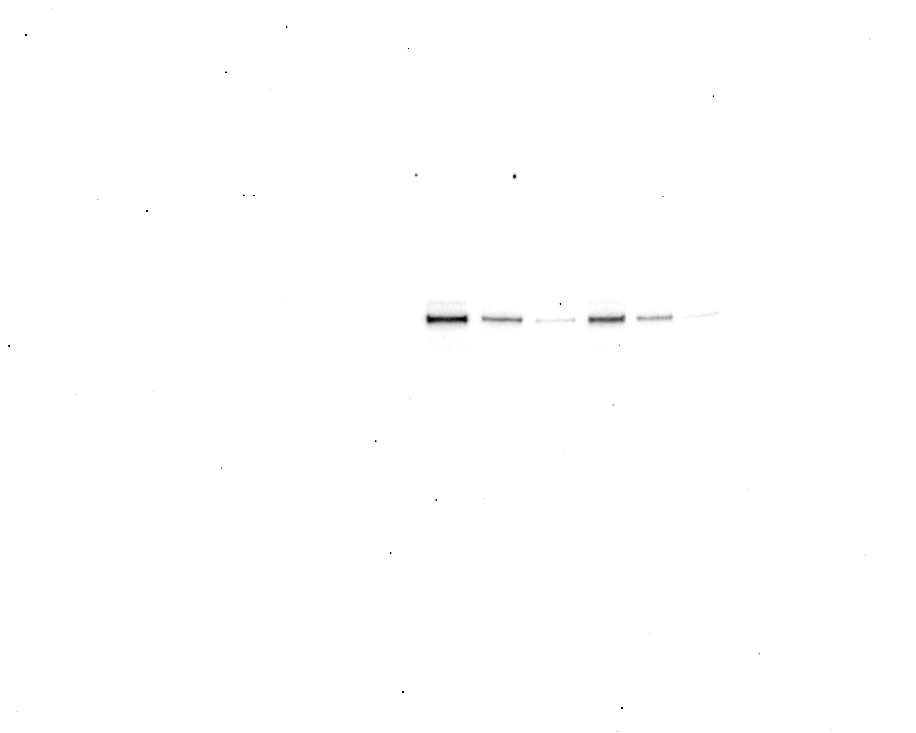

Supplement: Figure 1—figure supplement 1—source data 1. [file elife-87086-fig1-figsupp1-data1.zip › Figure 1-Figure Supplement 1-Source Data 1/Figure 1-figure supplement 1F/anti-FLAG (Halo-SHLD3) Western Blot.tif]

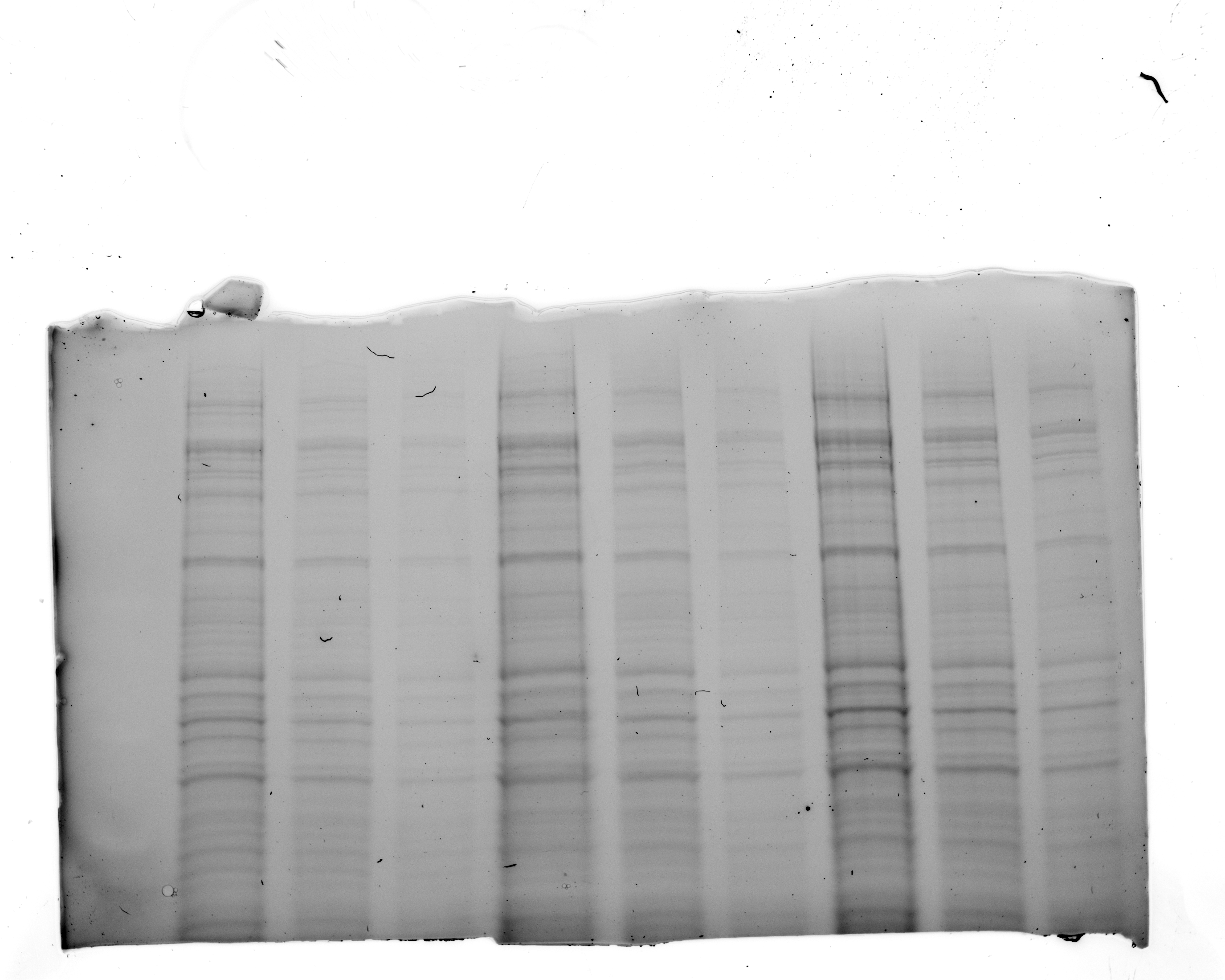

Supplement: Figure 1—figure supplement 1—source data 1. [file elife-87086-fig1-figsupp1-data1.zip › Figure 1-Figure Supplement 1-Source Data 1/Figure 1-figure supplement 1F/anti-MDC1 Stain-Free Loading Control.tif]

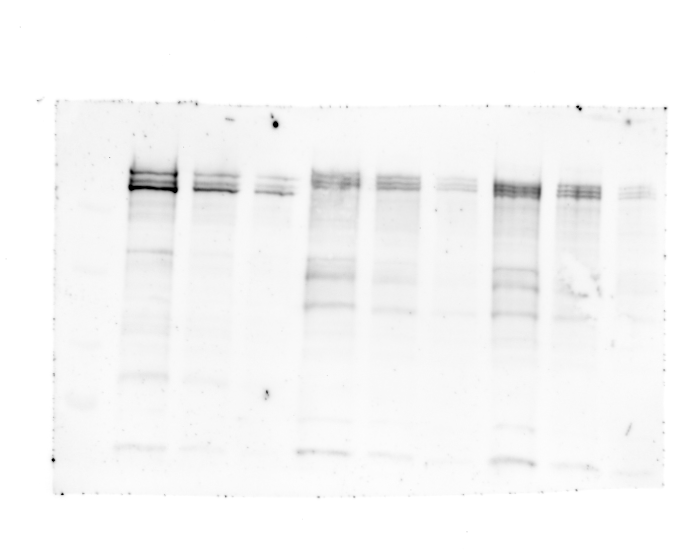

Supplement: Figure 1—figure supplement 1—source data 1. [file elife-87086-fig1-figsupp1-data1.zip › Figure 1-Figure Supplement 1-Source Data 1/Figure 1-figure supplement 1F/anti-MDC1 Western Blot.tif]

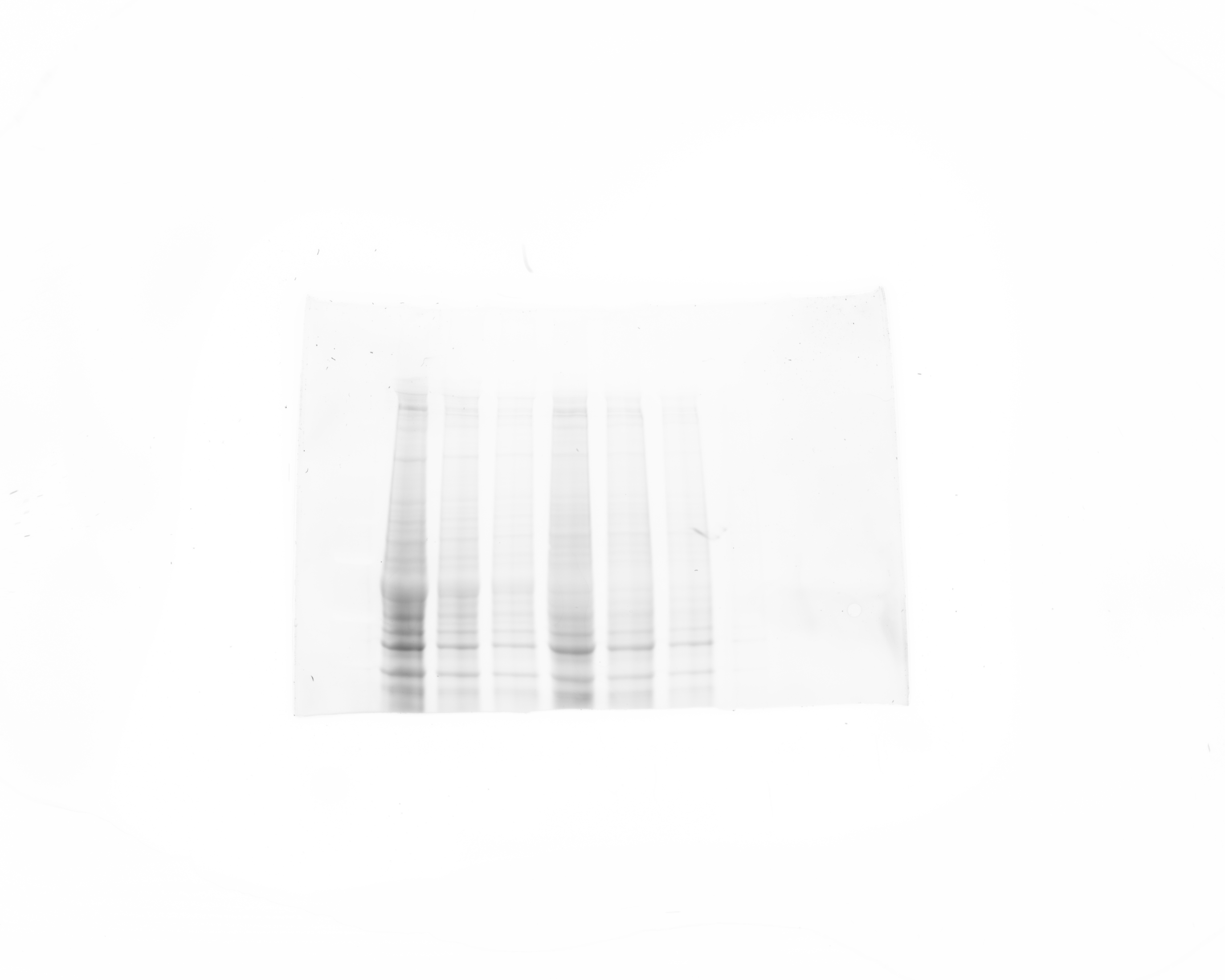

Supplement: Figure 1—figure supplement 1—source data 1. [file elife-87086-fig1-figsupp1-data1.zip › Figure 1-Figure Supplement 1-Source Data 1/Figure 1-figure supplement 1F/anti-NBS1 Stain-Free Loading Control.tif]

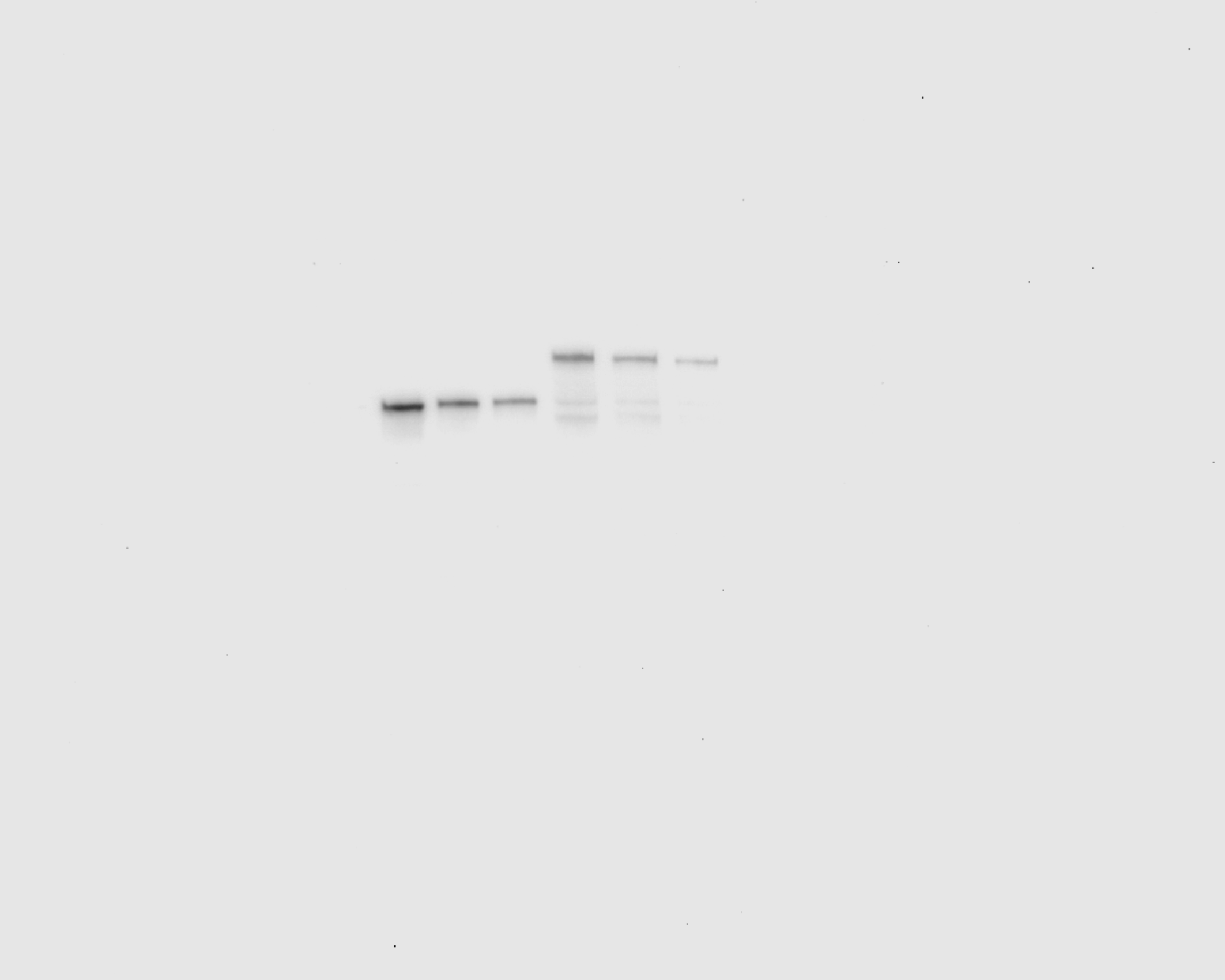

Supplement: Figure 1—figure supplement 1—source data 1. [file elife-87086-fig1-figsupp1-data1.zip › Figure 1-Figure Supplement 1-Source Data 1/Figure 1-figure supplement 1F/anti-NBS1 Western Blot.tif]

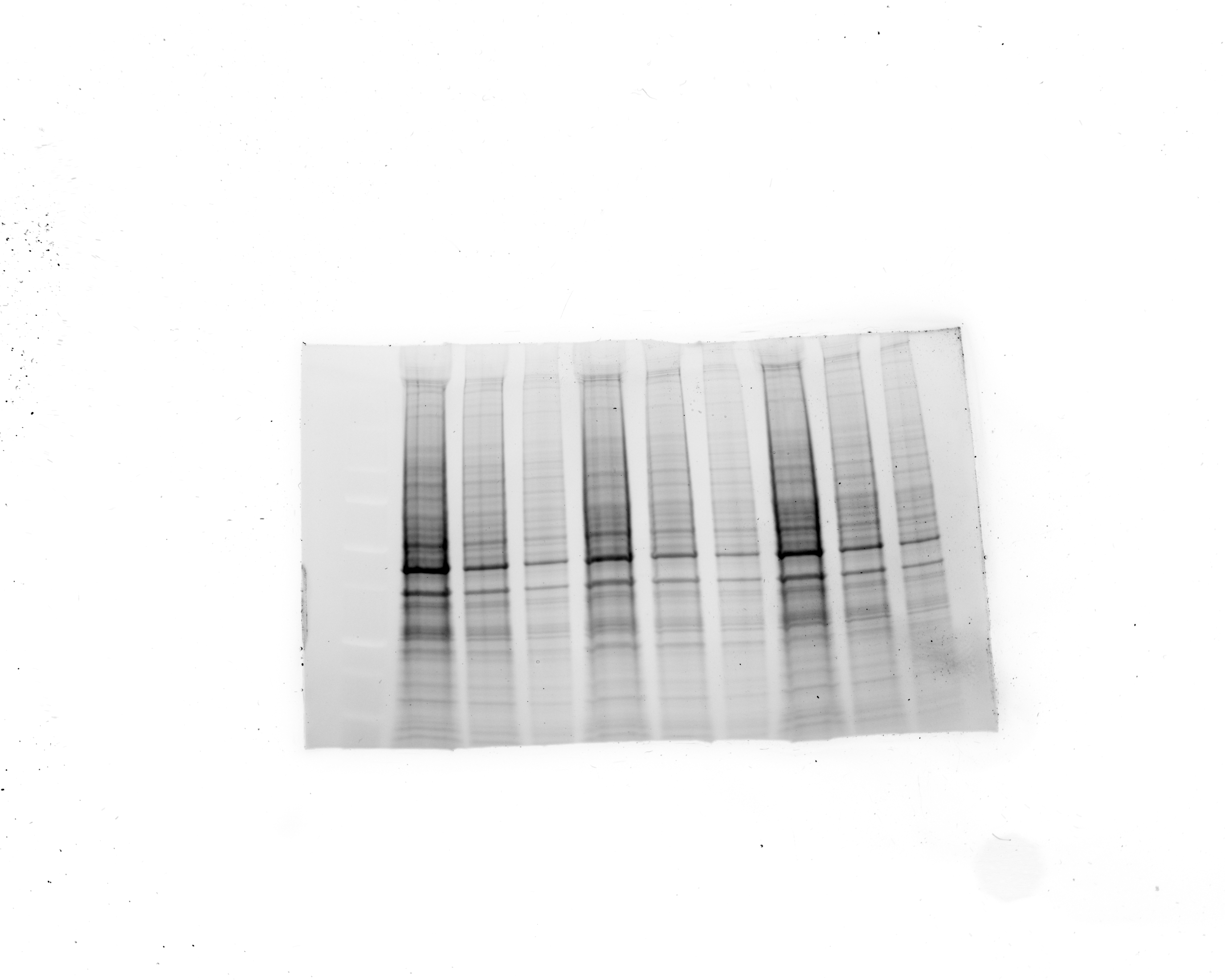

Supplement: Figure 1—figure supplement 1—source data 1. [file elife-87086-fig1-figsupp1-data1.zip › Figure 1-Figure Supplement 1-Source Data 1/Figure 1-figure supplement 1F/anti-REV7 Stain-Free Loading Control.tif]

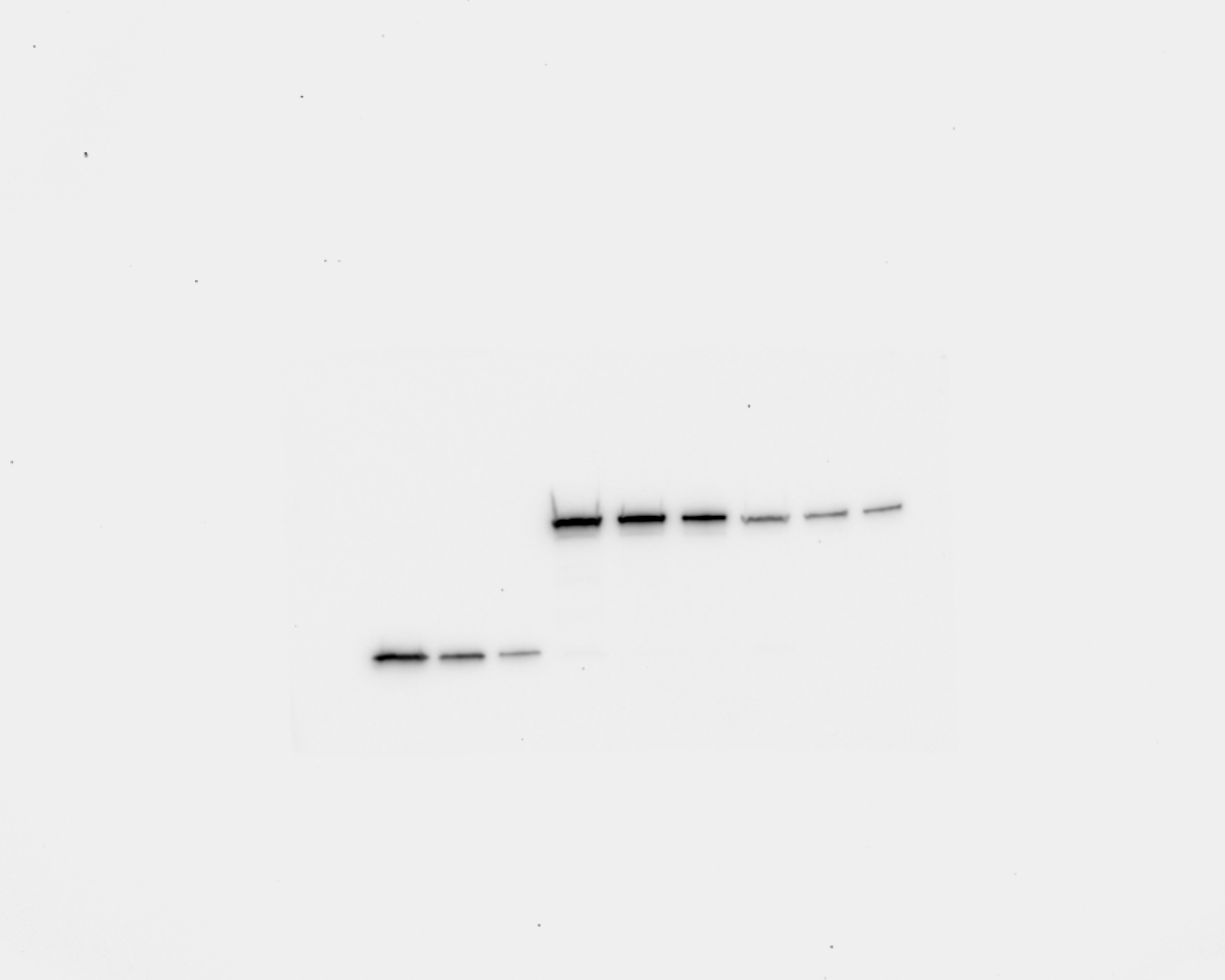

Supplement: Figure 1—figure supplement 1—source data 1. [file elife-87086-fig1-figsupp1-data1.zip › Figure 1-Figure Supplement 1-Source Data 1/Figure 1-figure supplement 1F/anti-REV7 Western Blot.tif]

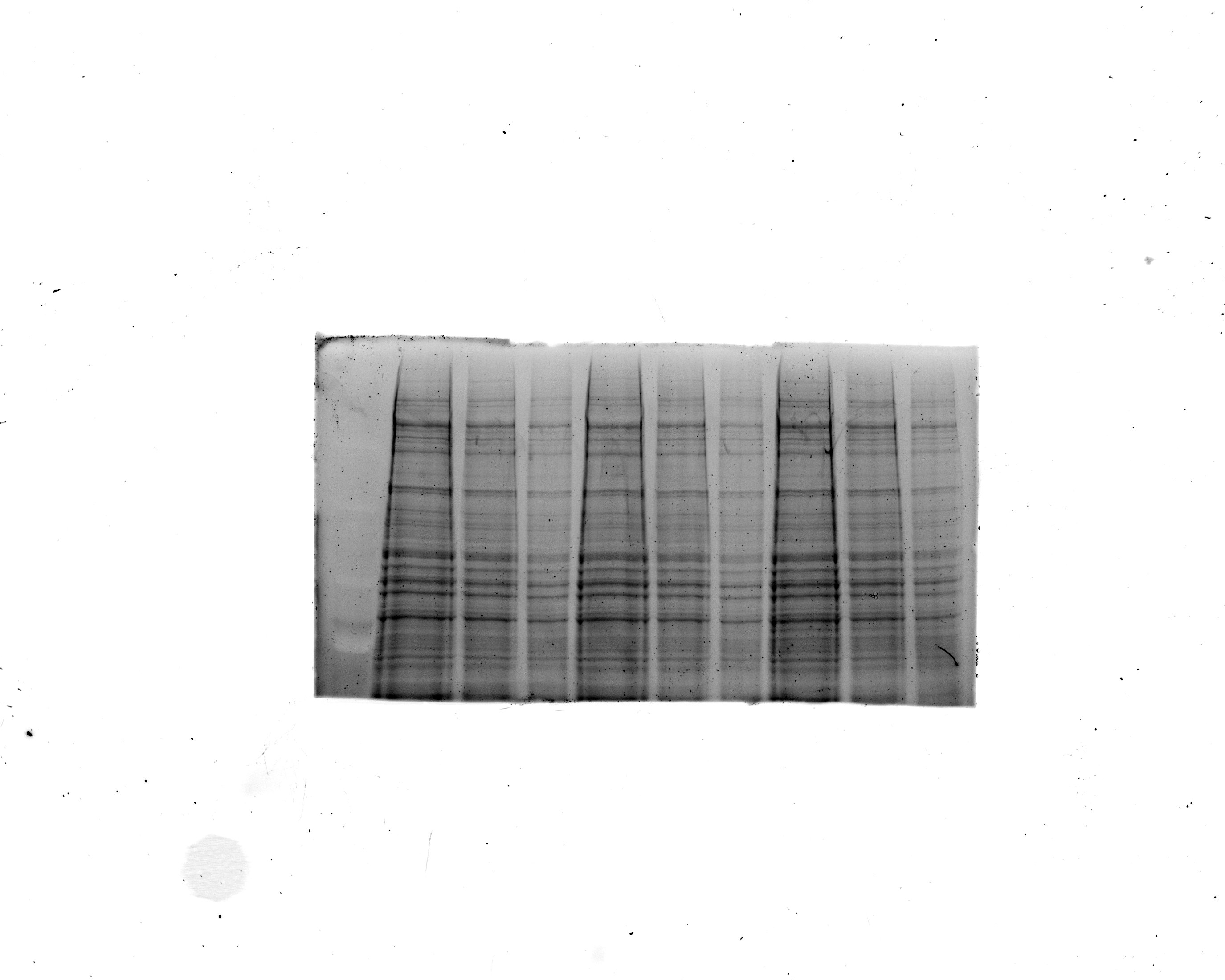

Supplement: Figure 1—figure supplement 1—source data 1. [file elife-87086-fig1-figsupp1-data1.zip › Figure 1-Figure Supplement 1-Source Data 1/Figure 1-figure supplement 1F/anti-RIF1 Stain-Free Loading Control.tif]

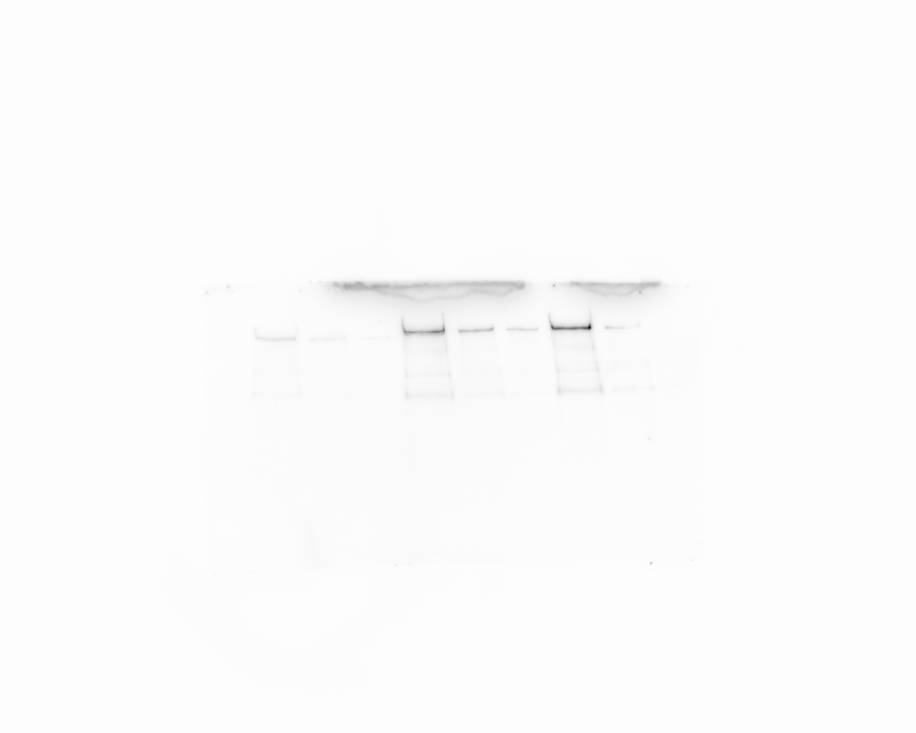

Supplement: Figure 1—figure supplement 1—source data 1. [file elife-87086-fig1-figsupp1-data1.zip › Figure 1-Figure Supplement 1-Source Data 1/Figure 1-figure supplement 1F/anti-RIF1 Western Blot.tif]

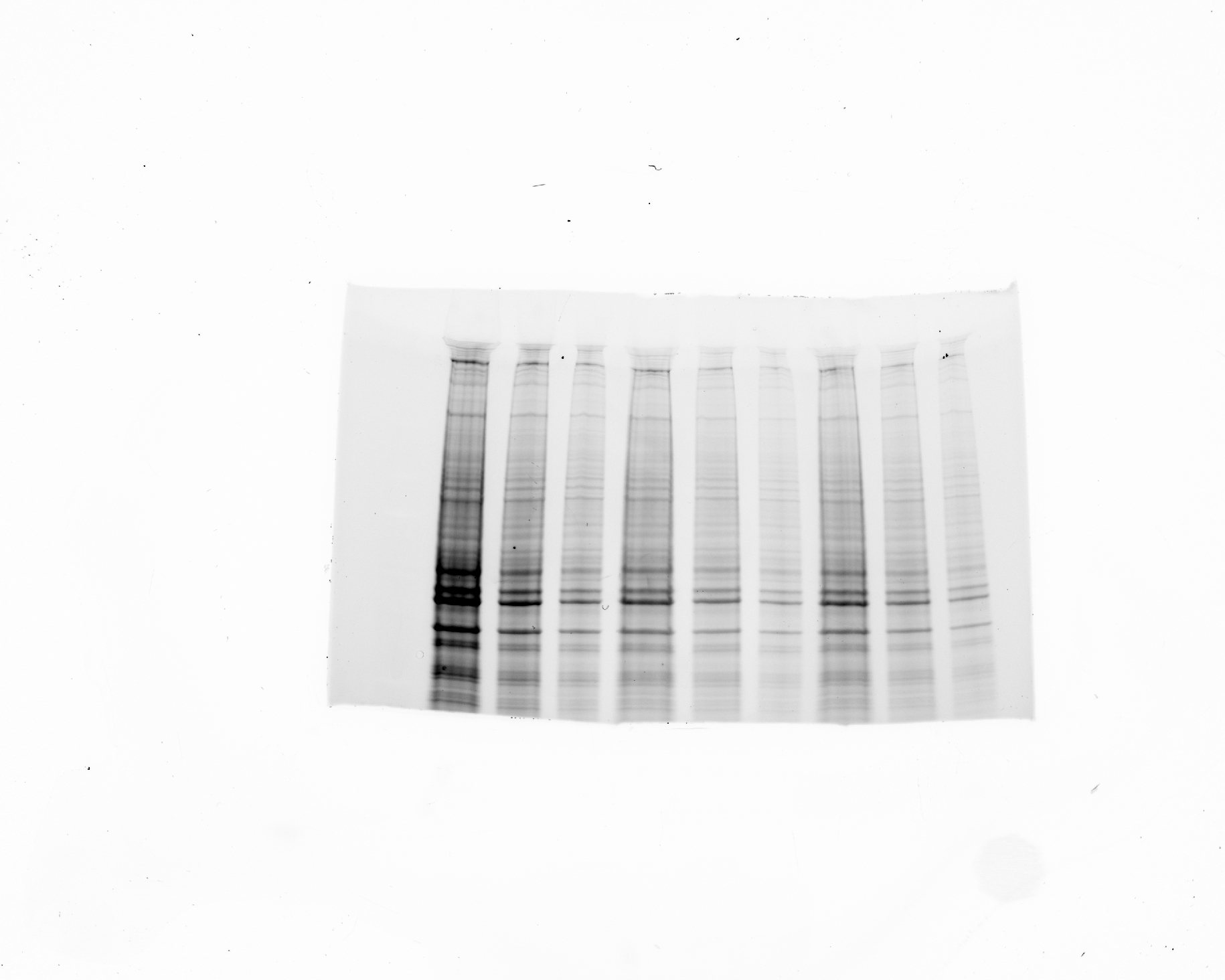

Supplement: Figure 1—figure supplement 1—source data 1. [file elife-87086-fig1-figsupp1-data1.zip › Figure 1-Figure Supplement 1-Source Data 1/Figure 1-figure supplement 1F/anti-RNF168 Stain-Free Loading Control.tif]

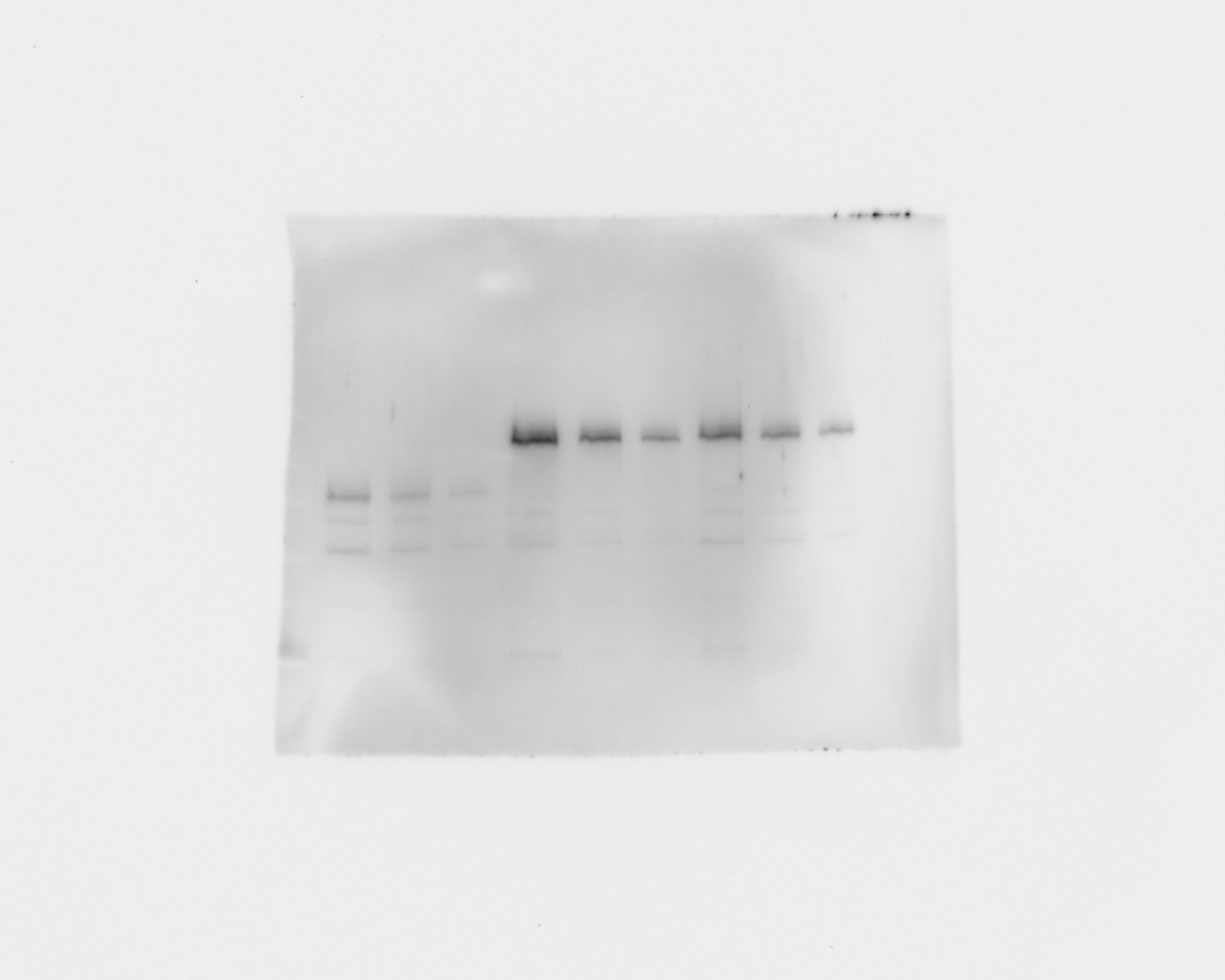

Supplement: Figure 1—figure supplement 1—source data 1. [file elife-87086-fig1-figsupp1-data1.zip › Figure 1-Figure Supplement 1-Source Data 1/Figure 1-figure supplement 1F/anti-RNF168 Western Blot.tif]

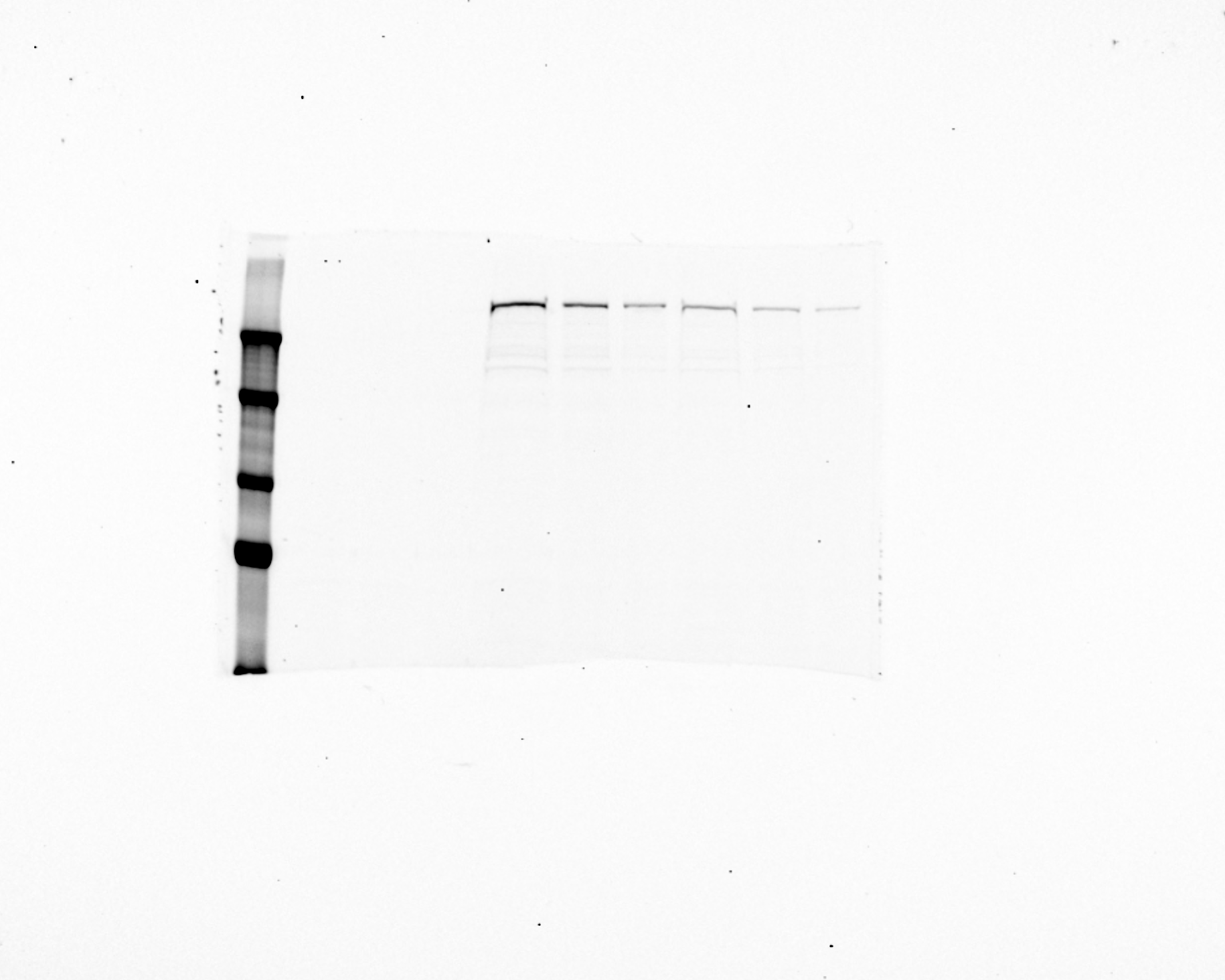

Supplement: Figure 2—figure supplement 1—source data 1. [file elife-87086-fig2-figsupp1-data1.zip › Figure 2-Figure Supplement 1-Source Data 1/Figure 2-figure supplement 1B/JFX650 Fluorescent Gel N-Term ATM Clones.tif]

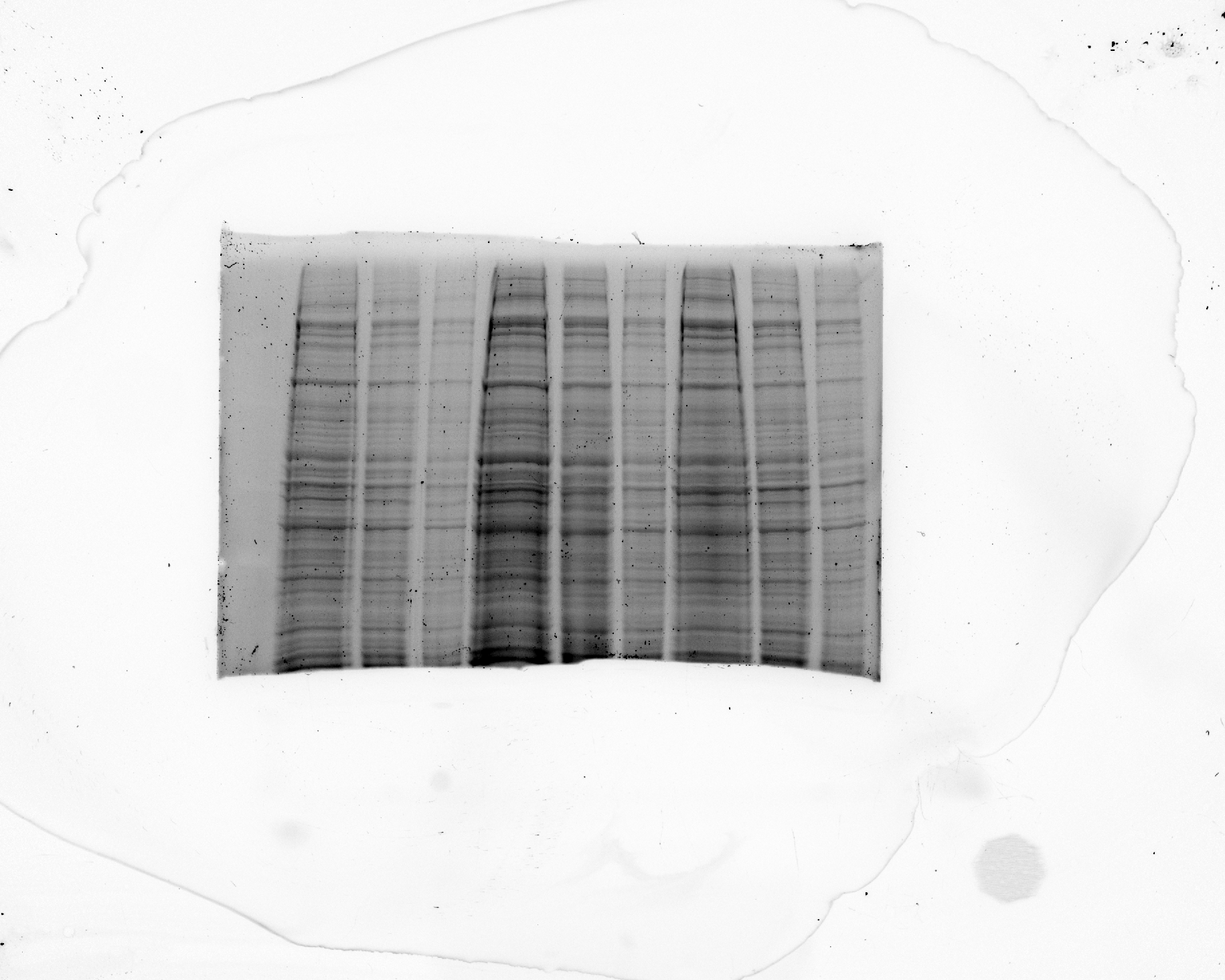

Supplement: Figure 2—figure supplement 1—source data 1. [file elife-87086-fig2-figsupp1-data1.zip › Figure 2-Figure Supplement 1-Source Data 1/Figure 2-figure supplement 1B/Stain-Free Loading Control.tif]

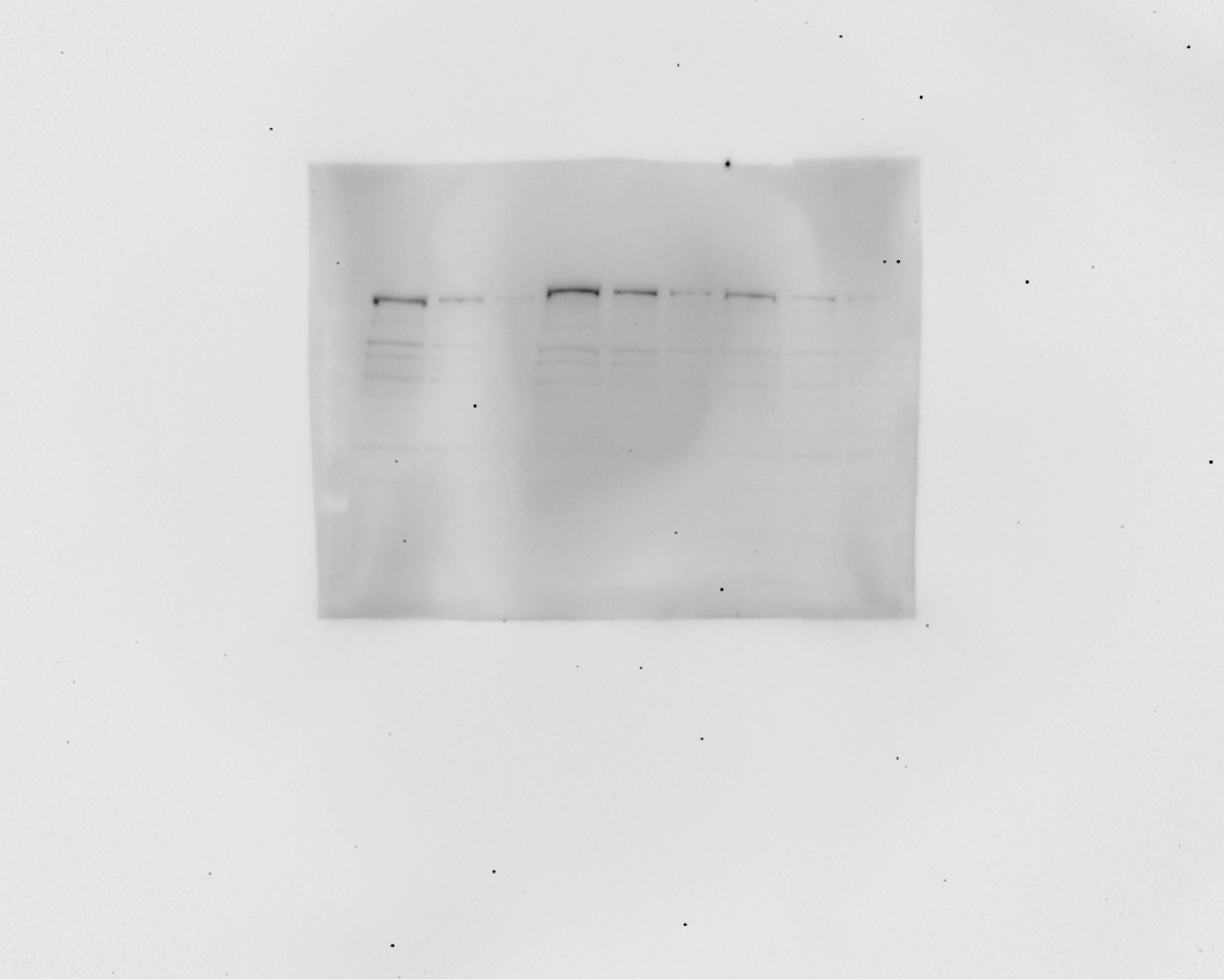

Supplement: Figure 2—figure supplement 1—source data 1. [file elife-87086-fig2-figsupp1-data1.zip › Figure 2-Figure Supplement 1-Source Data 1/Figure 2-figure supplement 1B/Western Blot detection of ATM.tif]

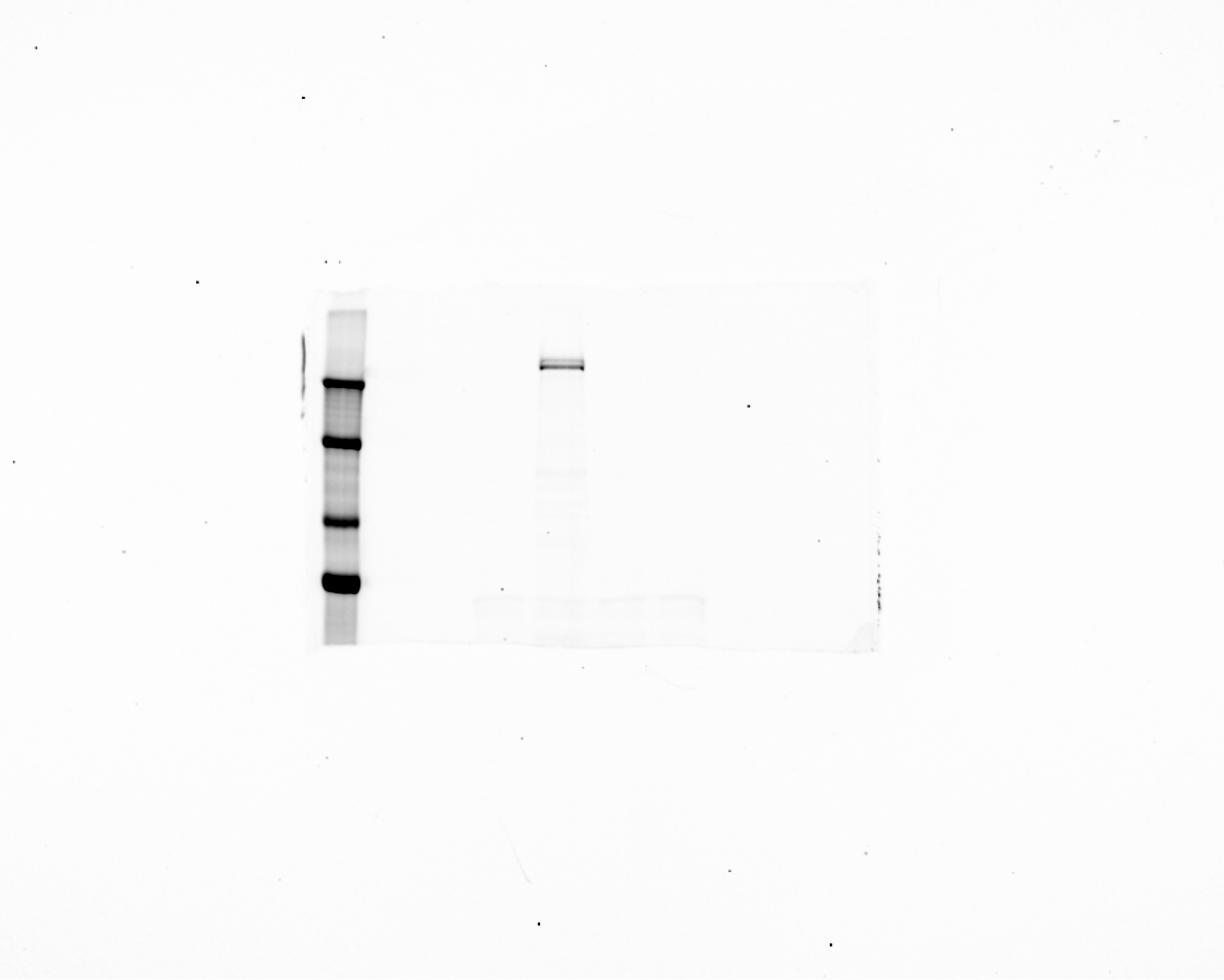

Supplement: Figure 2—figure supplement 1—source data 1. [file elife-87086-fig2-figsupp1-data1.zip › Figure 2-Figure Supplement 1-Source Data 1/Figure 2-figure supplement 1E/JFX650 Fluorescent Gel of HaloTag MDC1 and knockout clones.tif]

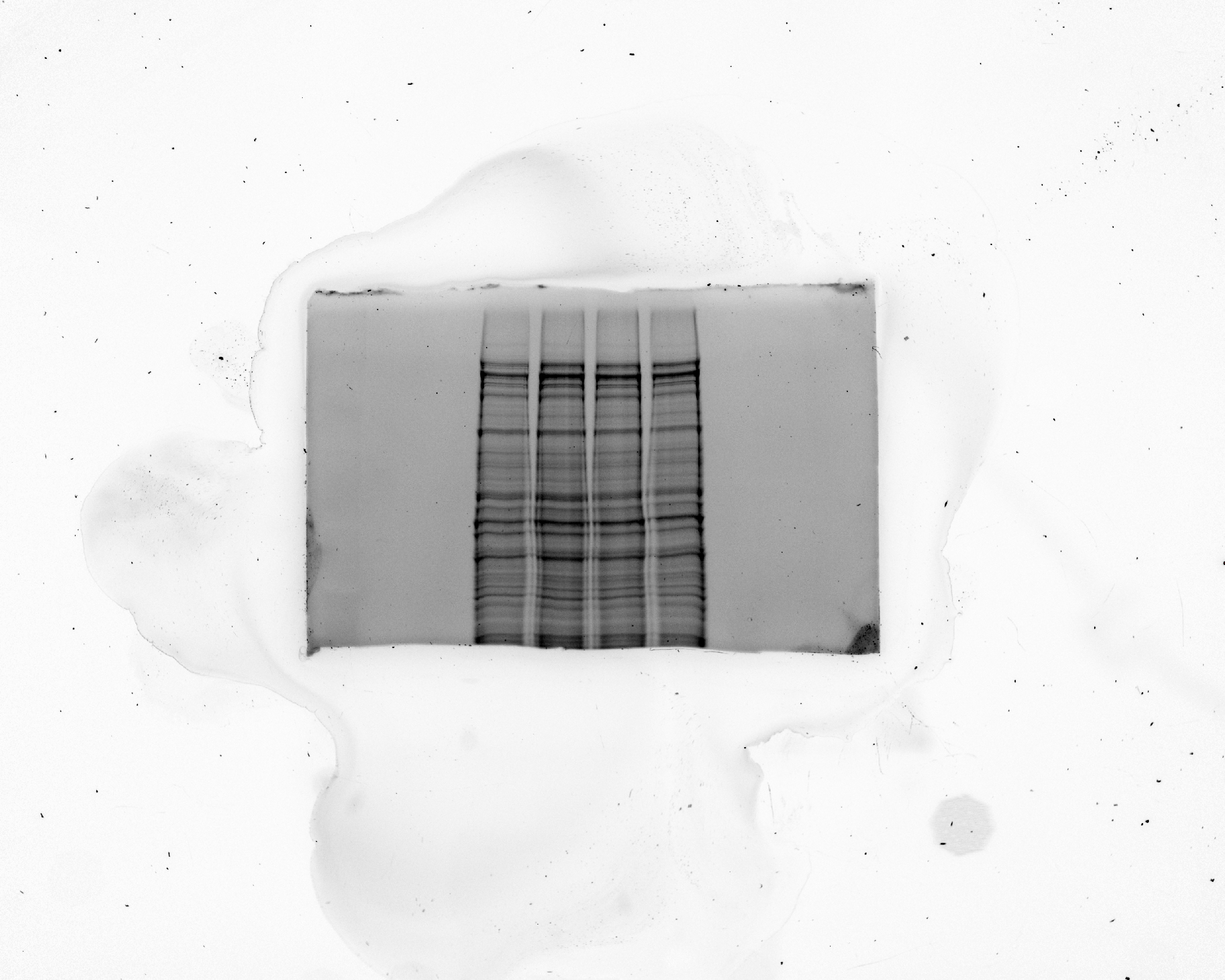

Supplement: Figure 2—figure supplement 1—source data 1. [file elife-87086-fig2-figsupp1-data1.zip › Figure 2-Figure Supplement 1-Source Data 1/Figure 2-figure supplement 1E/Stain-Free Loading Control.tif]

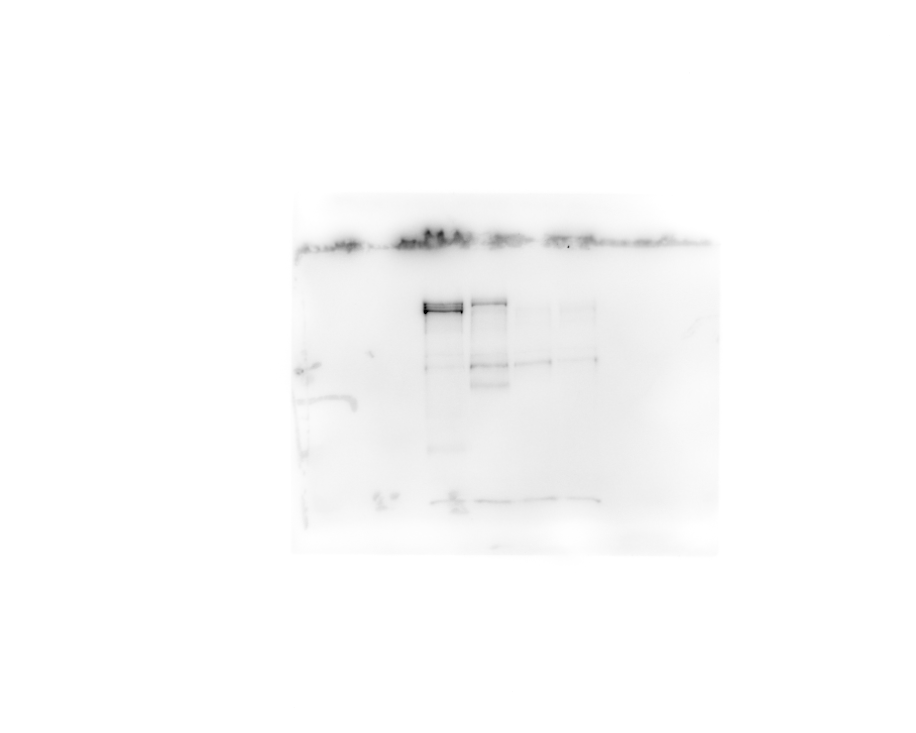

Supplement: Figure 2—figure supplement 1—source data 1. [file elife-87086-fig2-figsupp1-data1.zip › Figure 2-Figure Supplement 1-Source Data 1/Figure 2-figure supplement 1E/Western Blot of MDC1.tif]

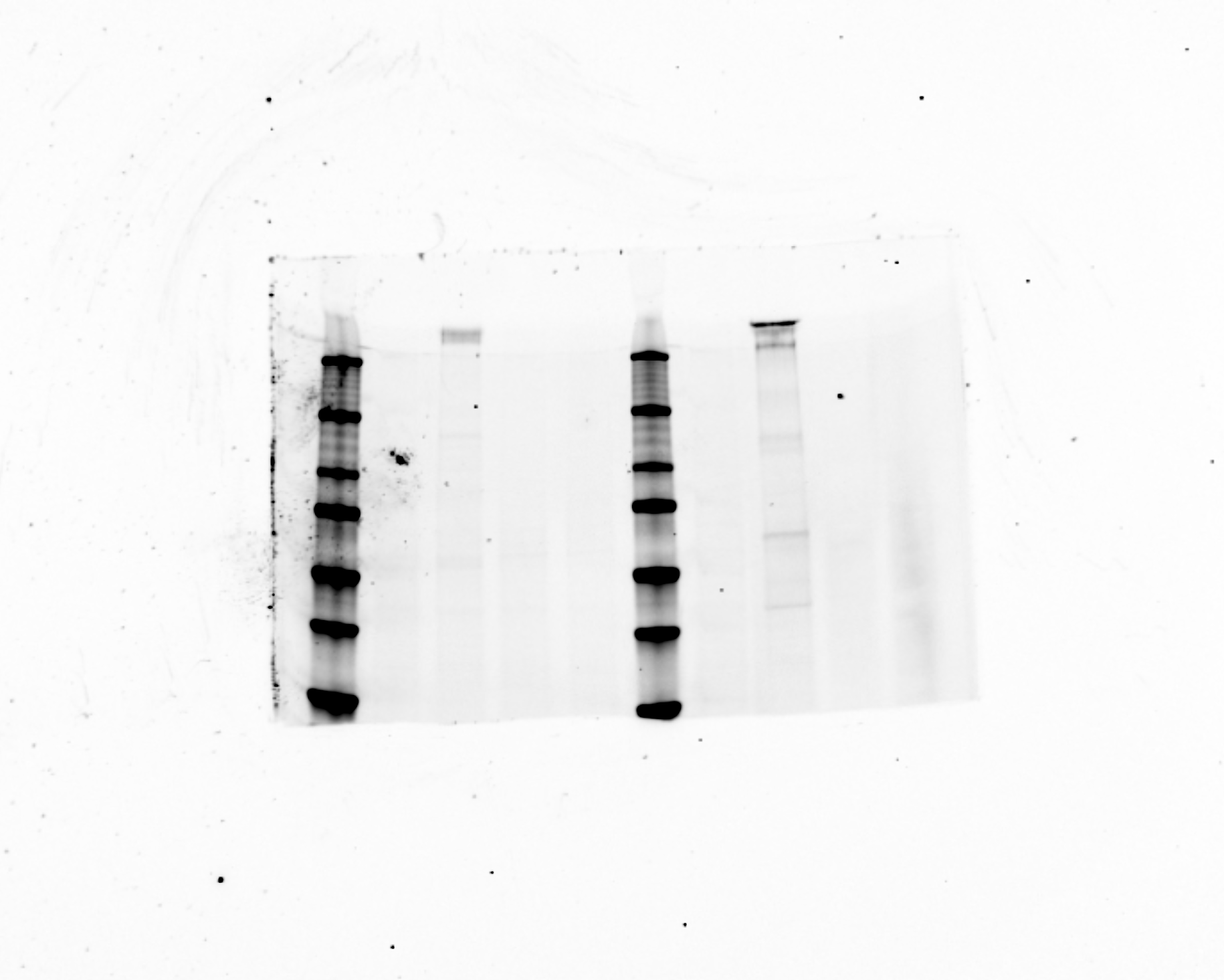

Supplement: Figure 2—figure supplement 1—source data 1. [file elife-87086-fig2-figsupp1-data1.zip › Figure 2-Figure Supplement 1-Source Data 1/Figure 2-figure supplement 1F/JFX650 Fluorescent Gel HaloTag 53BP1 and knockout clones.tif]

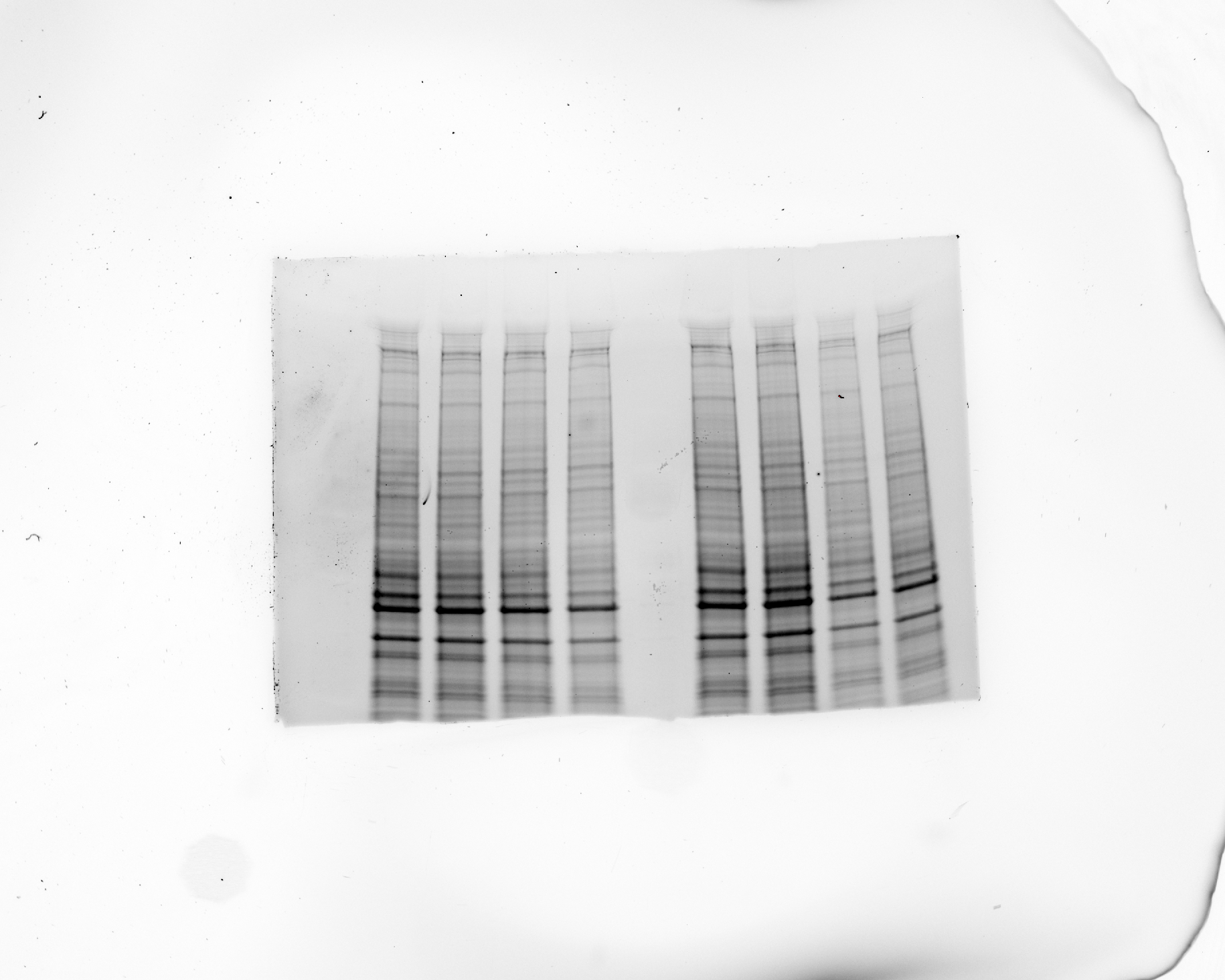

Supplement: Figure 2—figure supplement 1—source data 1. [file elife-87086-fig2-figsupp1-data1.zip › Figure 2-Figure Supplement 1-Source Data 1/Figure 2-figure supplement 1F/Stain-Free Loading Control.tif]

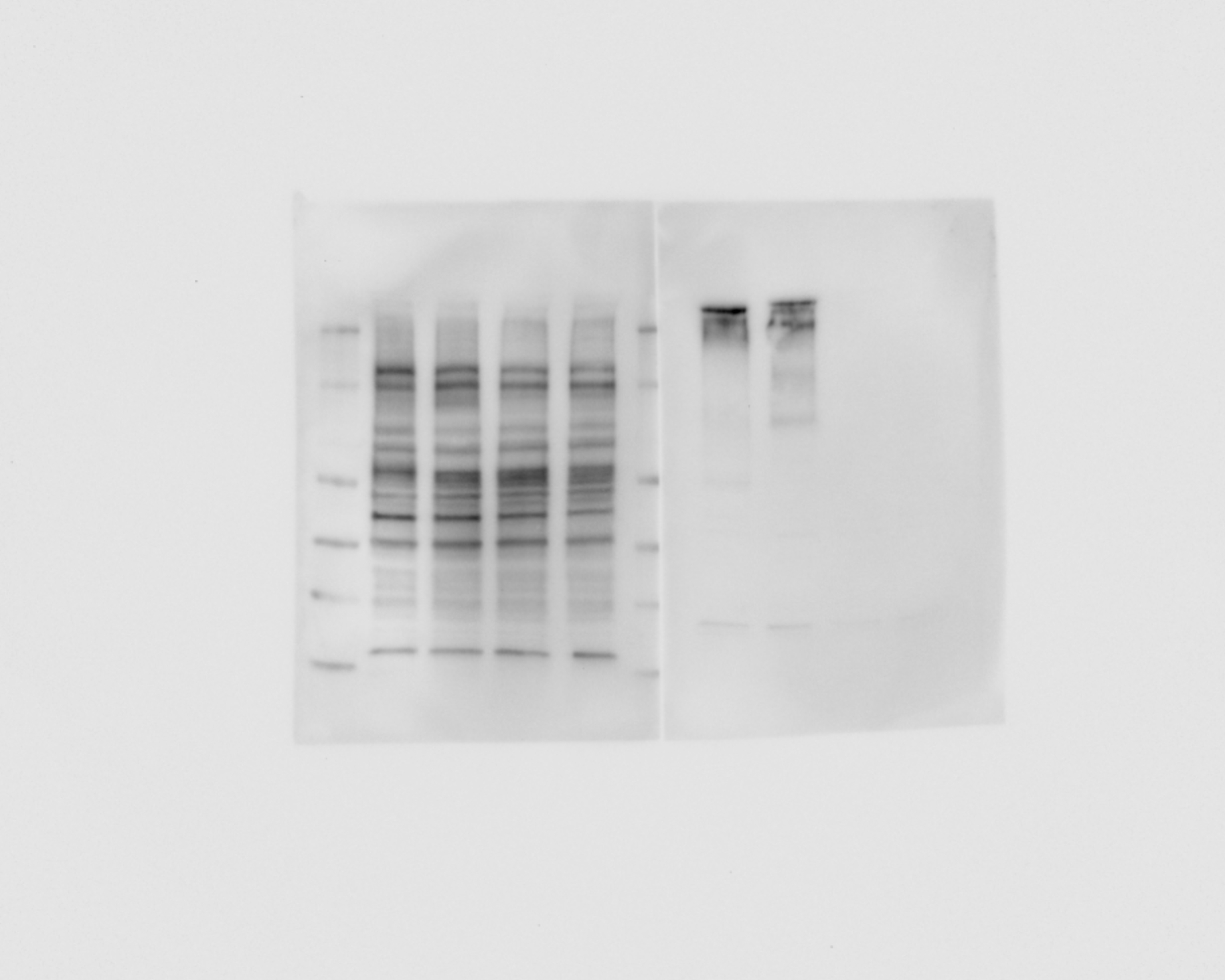

Supplement: Figure 2—figure supplement 1—source data 1. [file elife-87086-fig2-figsupp1-data1.zip › Figure 2-Figure Supplement 1-Source Data 1/Figure 2-figure supplement 1F/Western Blot of 53BP1.tif]

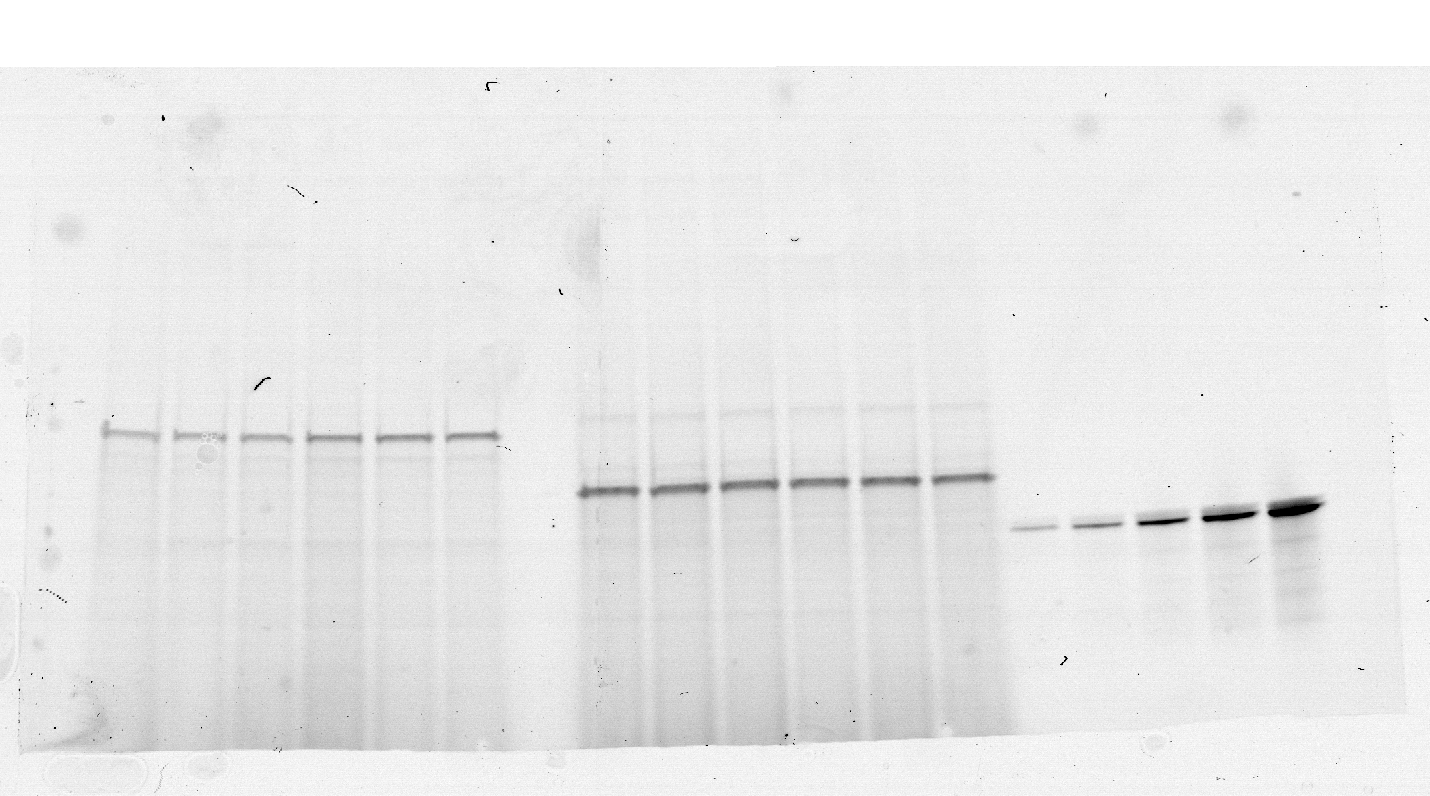

Supplement: Figure 3—source data 1. [file elife-87086-fig3-data1.zip › Figure 3-Source Data 1/Figure 3A/JF646 Fluorescent Gel of SHLD1 and SHLD3 HaloTag Clones.tif]

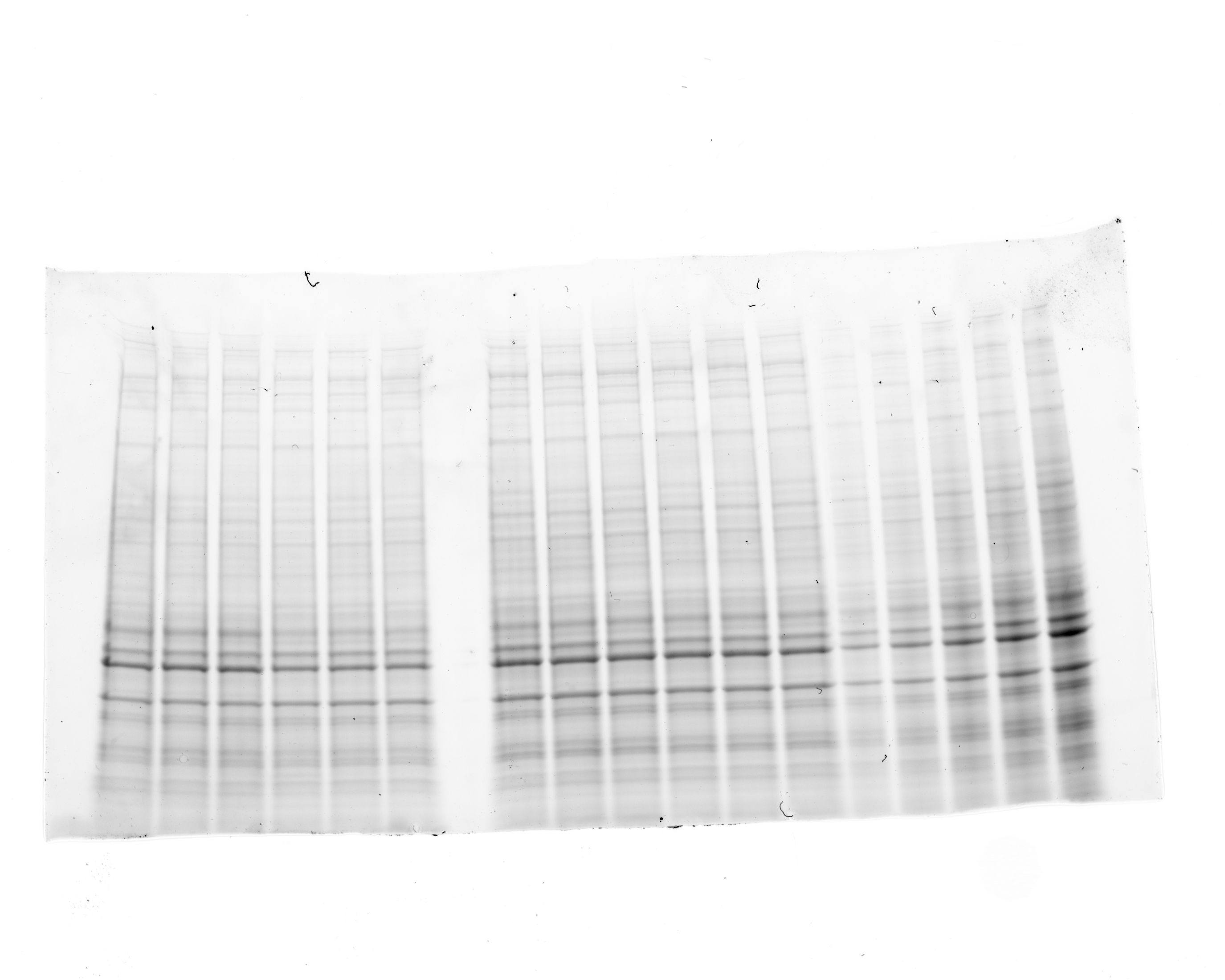

Supplement: Figure 3—source data 1. [file elife-87086-fig3-data1.zip › Figure 3-Source Data 1/Figure 3A/Stain-Free Loading Control.tif]

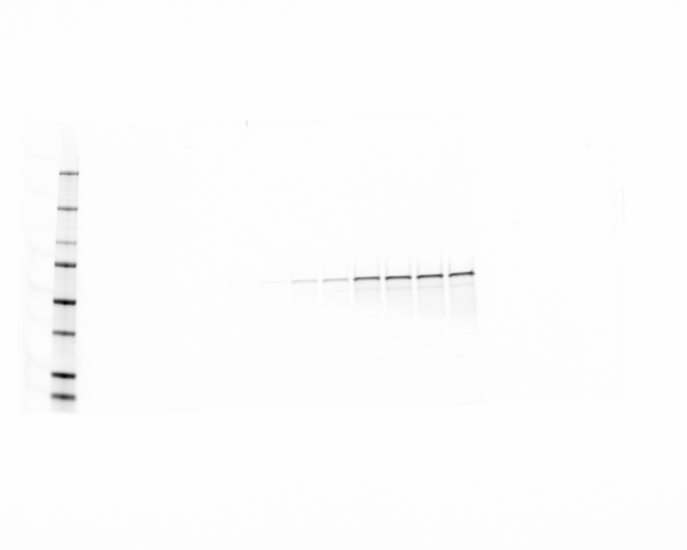

Supplement: Figure 3—figure supplement 1—source data 1. [file elife-87086-fig3-figsupp1-data1.zip › Figure 3-Figure Supplement 1-Source Data 1/Figure 3-figure supplement 1A/REV7 JF646 Saturation Fluorescent Gel.tif]

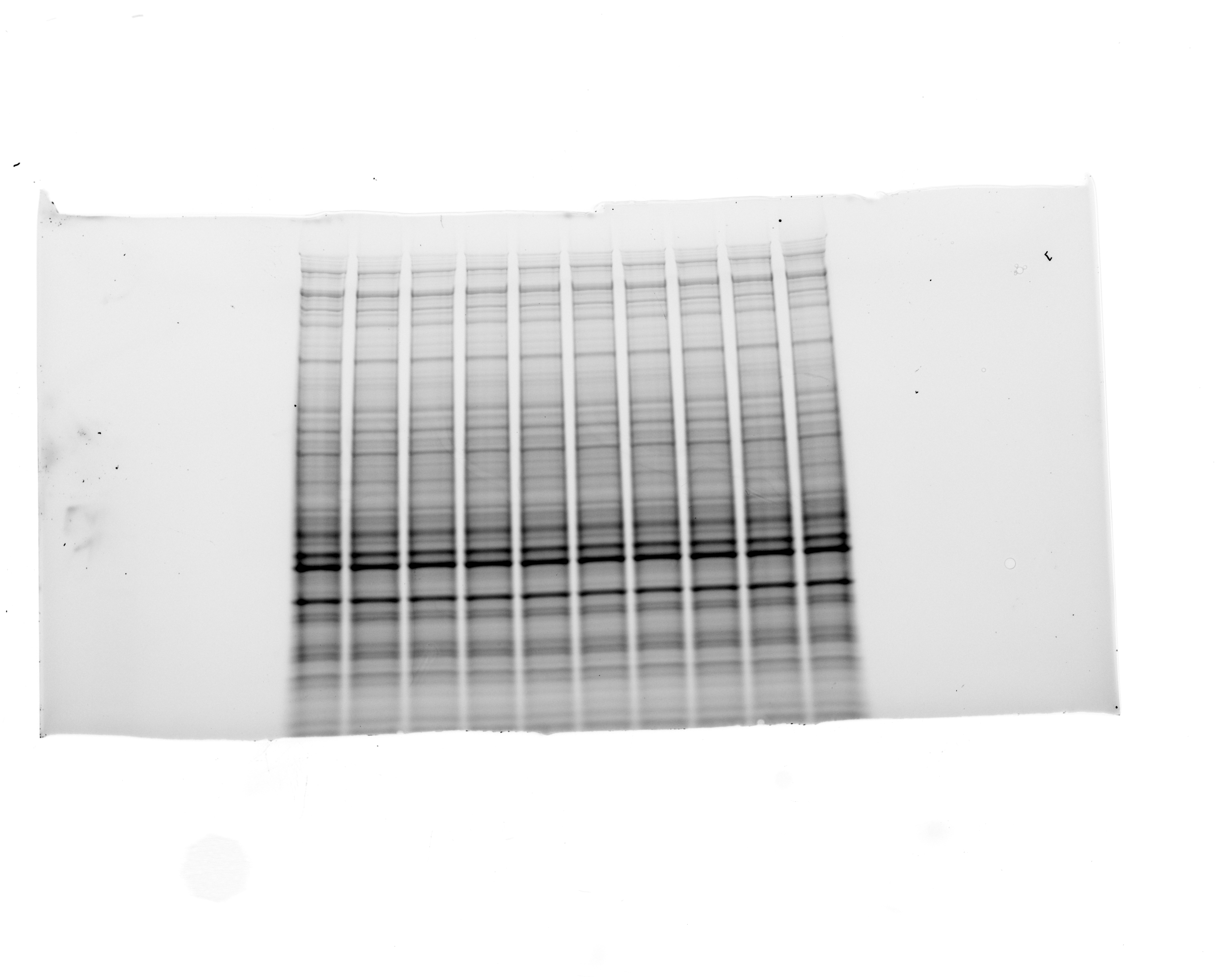

Supplement: Figure 3—figure supplement 1—source data 1. [file elife-87086-fig3-figsupp1-data1.zip › Figure 3-Figure Supplement 1-Source Data 1/Figure 3-figure supplement 1A/Stain-Free Loading Control.tif]

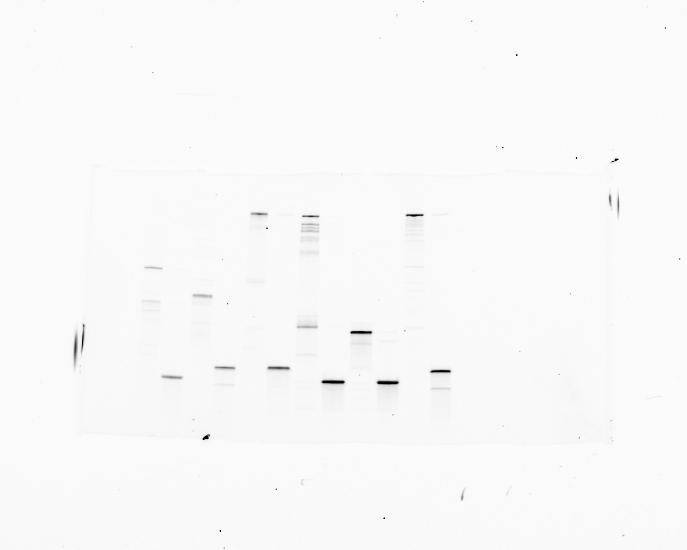

Supplement: Figure 3—figure supplement 1—source data 1. [file elife-87086-fig3-figsupp1-data1.zip › Figure 3-Figure Supplement 1-Source Data 1/Figure 3-figure supplement 1B/JF646 Fluorescent Gel TEV Supp 3B Left Panel.tif]

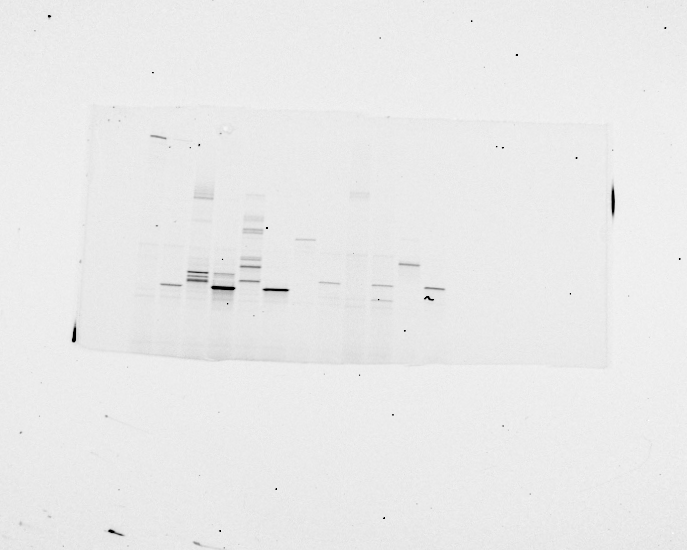

Supplement: Figure 3—figure supplement 1—source data 1. [file elife-87086-fig3-figsupp1-data1.zip › Figure 3-Figure Supplement 1-Source Data 1/Figure 3-figure supplement 1B/JF646 Fluorescent Gel TEV Supp 3B Right Panel.tif]

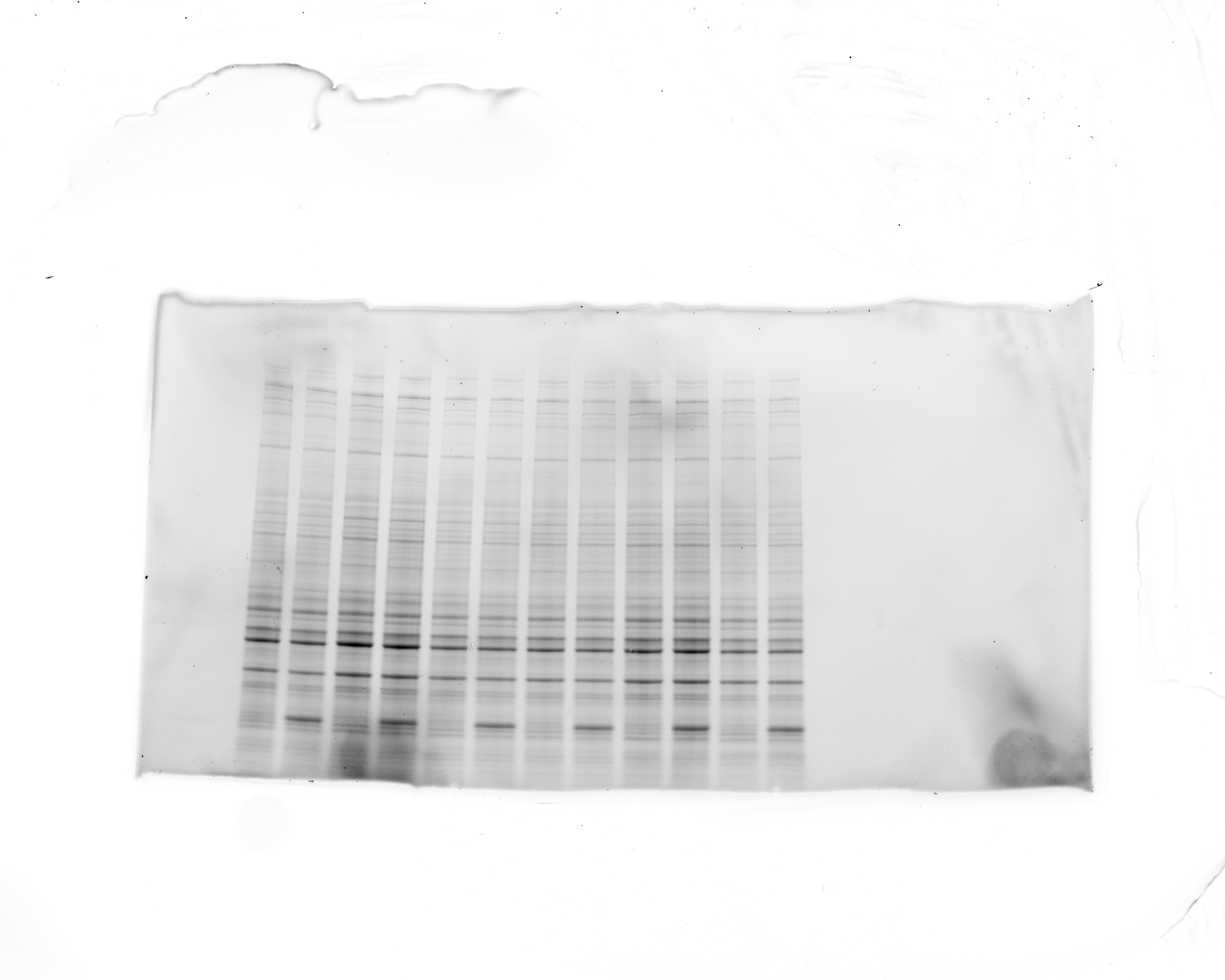

Supplement: Figure 3—figure supplement 1—source data 1. [file elife-87086-fig3-figsupp1-data1.zip › Figure 3-Figure Supplement 1-Source Data 1/Figure 3-figure supplement 1B/Stain-Free Loading Control Supp 3B Left Panel.tif]

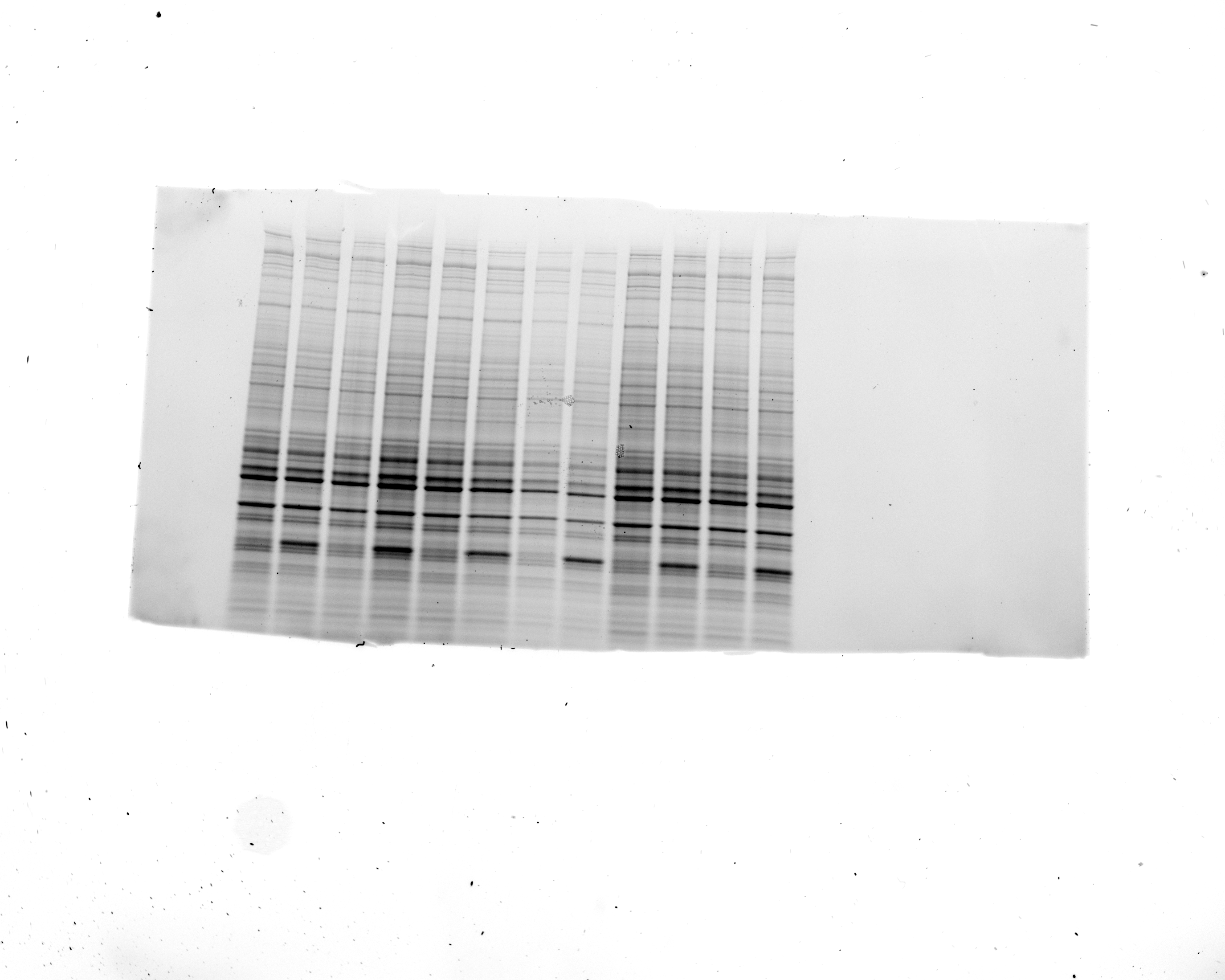

Supplement: Figure 3—figure supplement 1—source data 1. [file elife-87086-fig3-figsupp1-data1.zip › Figure 3-Figure Supplement 1-Source Data 1/Figure 3-figure supplement 1B/Stain-Free Loading Control Supp 3B Right Panel.tif]

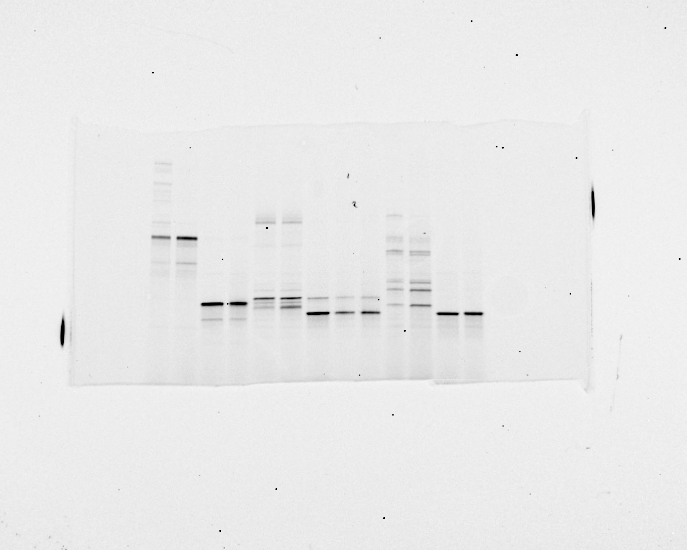

Supplement: Figure 3—figure supplement 1—source data 1. [file elife-87086-fig3-figsupp1-data1.zip › Figure 3-Figure Supplement 1-Source Data 1/Figure 3-figure supplement 1C/JF646 Fluorescent Gel Supp Fig 3C.tif]

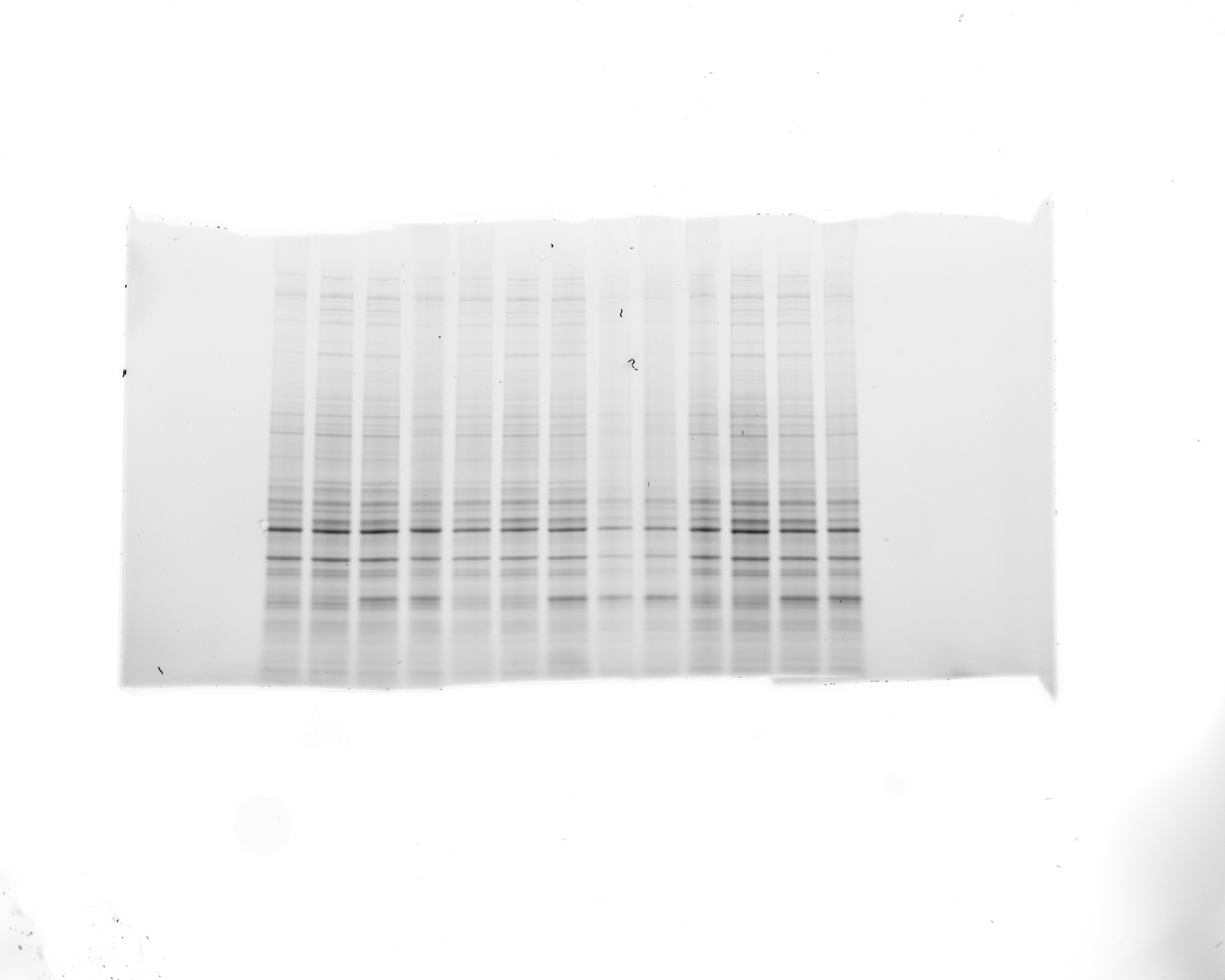

Supplement: Figure 3—figure supplement 1—source data 1. [file elife-87086-fig3-figsupp1-data1.zip › Figure 3-Figure Supplement 1-Source Data 1/Figure 3-figure supplement 1C/Stain-Free Loading Control Supp Fig 3C.tif]

Figure 5-figure supplement 1-source data 1

Figure 5—figure supplement 1A

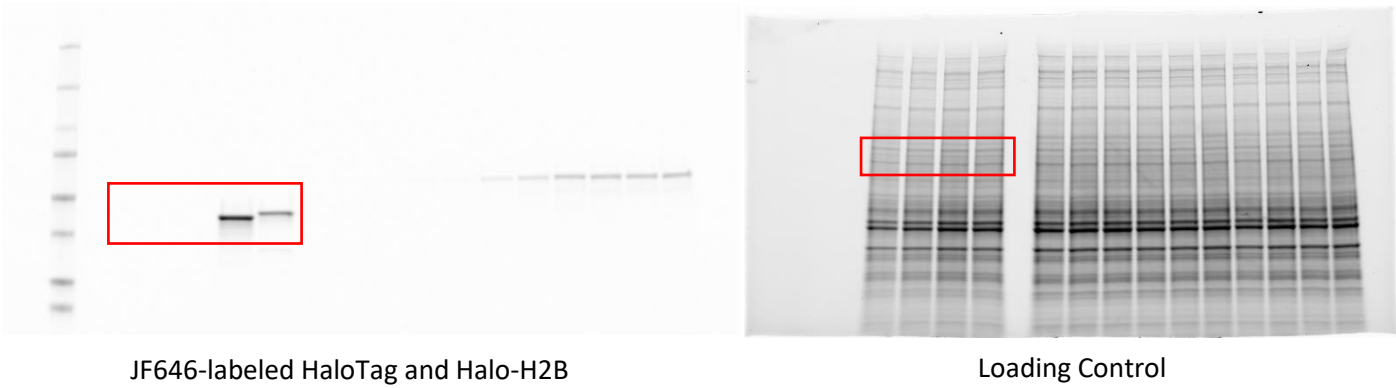

Supplement: Figure 5—figure supplement 1—source data 1. [file elife-87086-fig5-figsupp1-data1.zip › Figure 5-Figure Supplement 1-Source Data/Figure 5 - figure supplement 1 - source data 1.pdf]

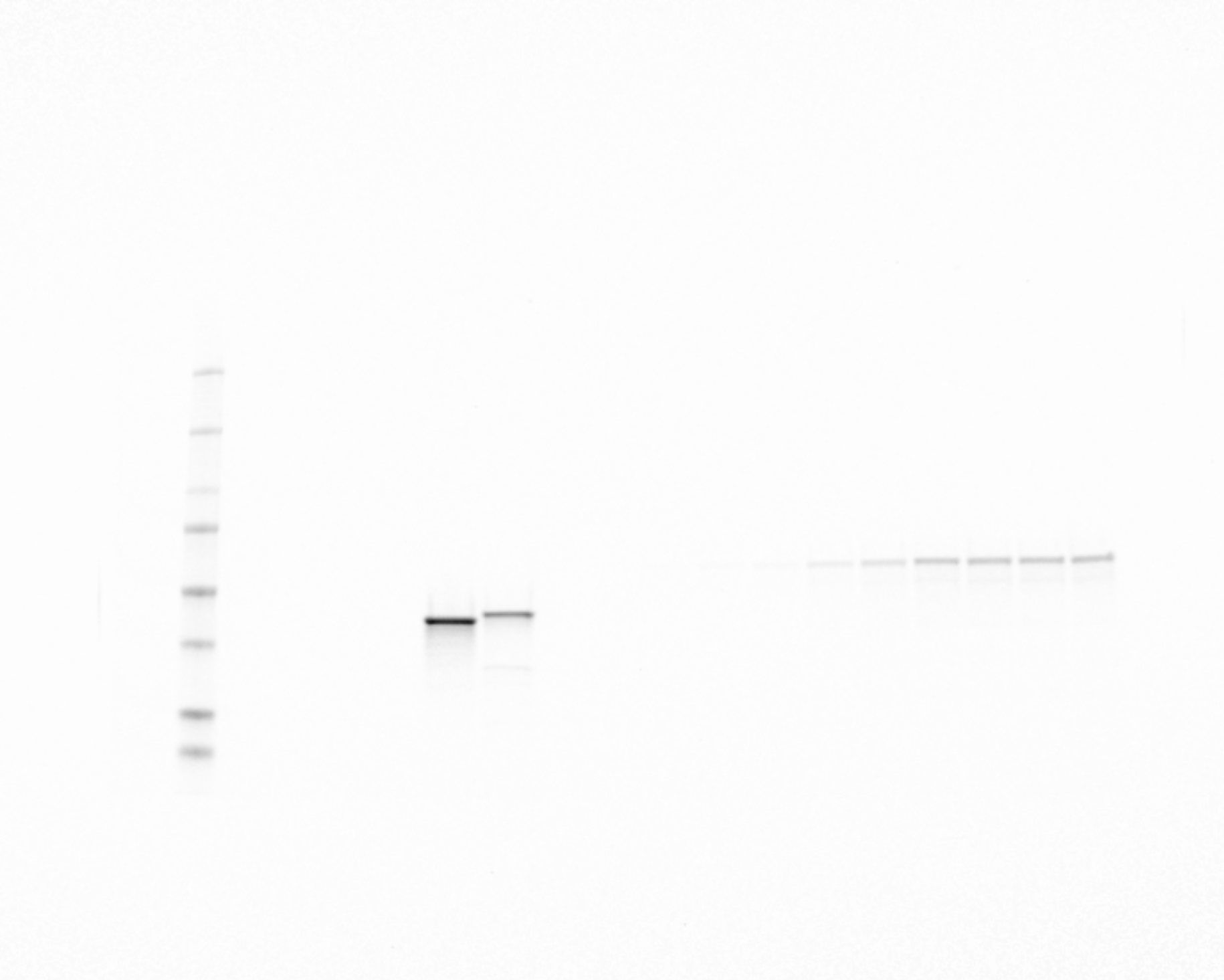

Supplement: Figure 5—figure supplement 1—source data 1. [file elife-87086-fig5-figsupp1-data1.zip › Figure 5-Figure Supplement 1-Source Data/Figure 5--figure supplement 1A/JF646 Fluorescent Gel Expression of Halo-NLS and Halo-H2B.tif]

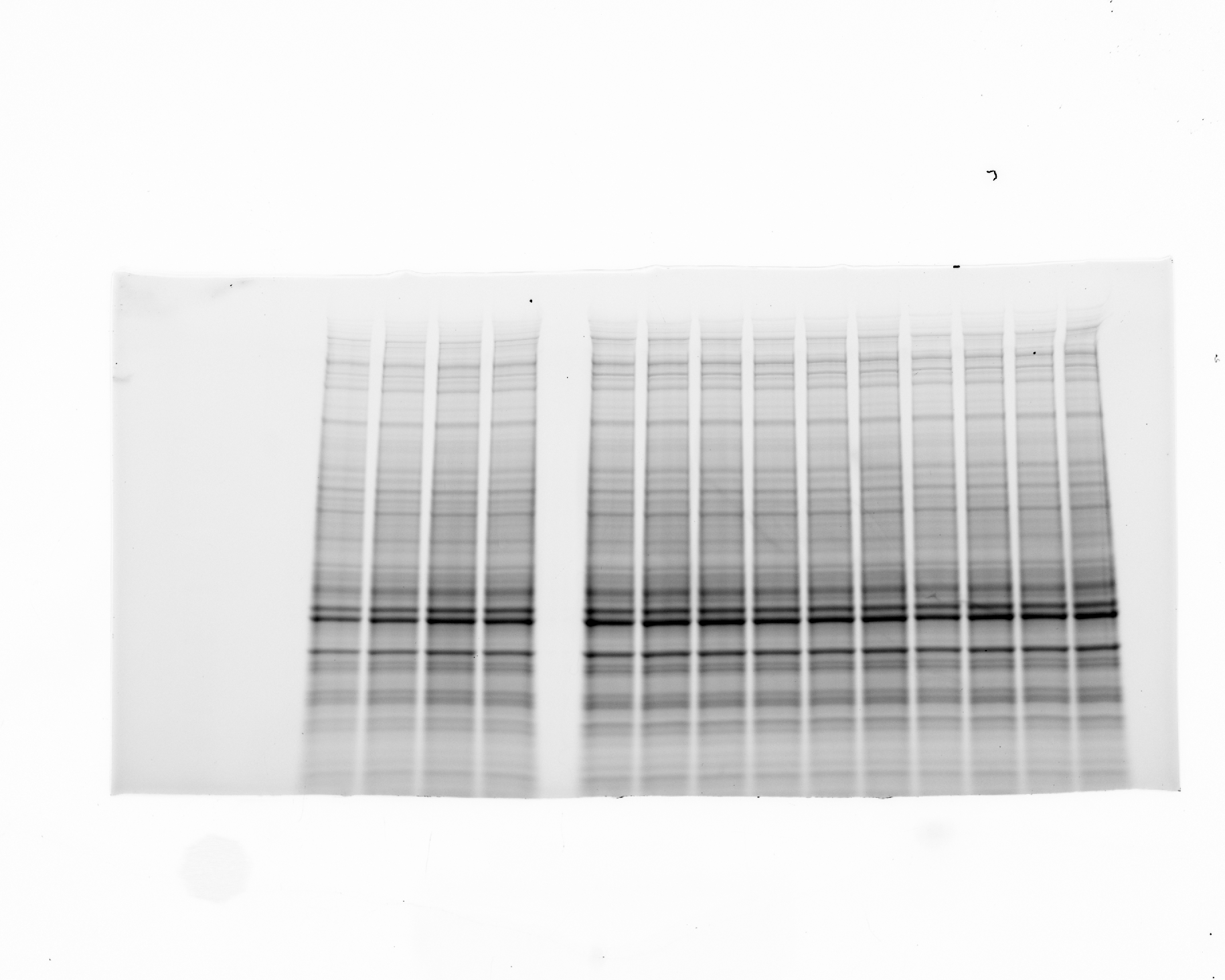

Supplement: Figure 5—figure supplement 1—source data 1. [file elife-87086-fig5-figsupp1-data1.zip › Figure 5-Figure Supplement 1-Source Data/Figure 5--figure supplement 1A/Stain-Free Loading Control.tif]

Figure 7-source data 1

Figure 7B

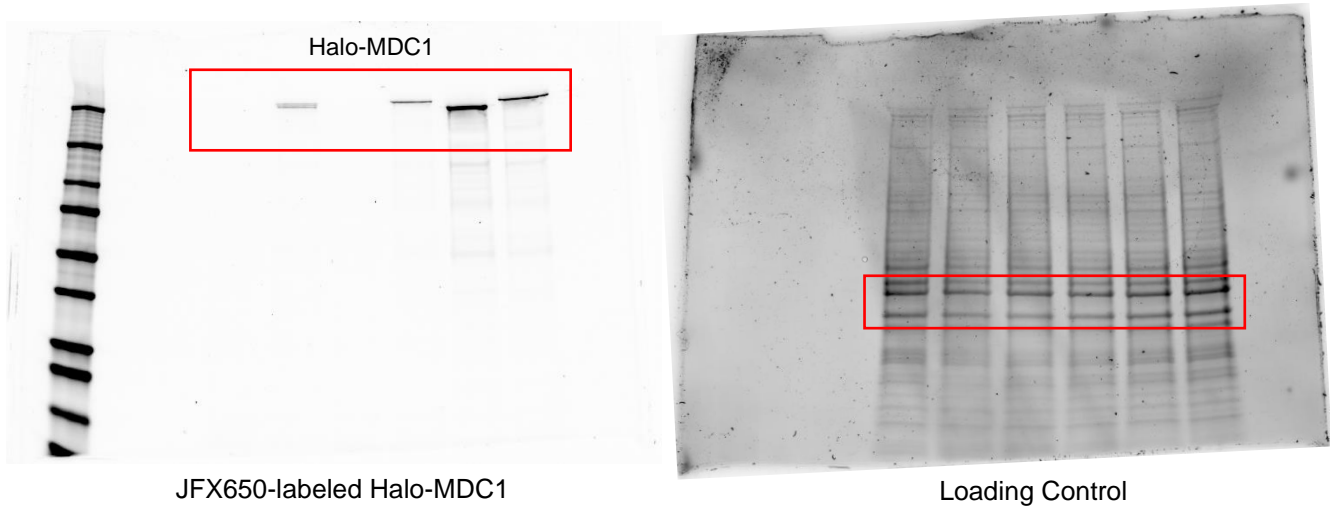

Supplement: Figure 7—source data 1. [file elife-87086-fig7-data1.zip › Figure 7-Source Data 1/Figure 7- source data 1.pdf]

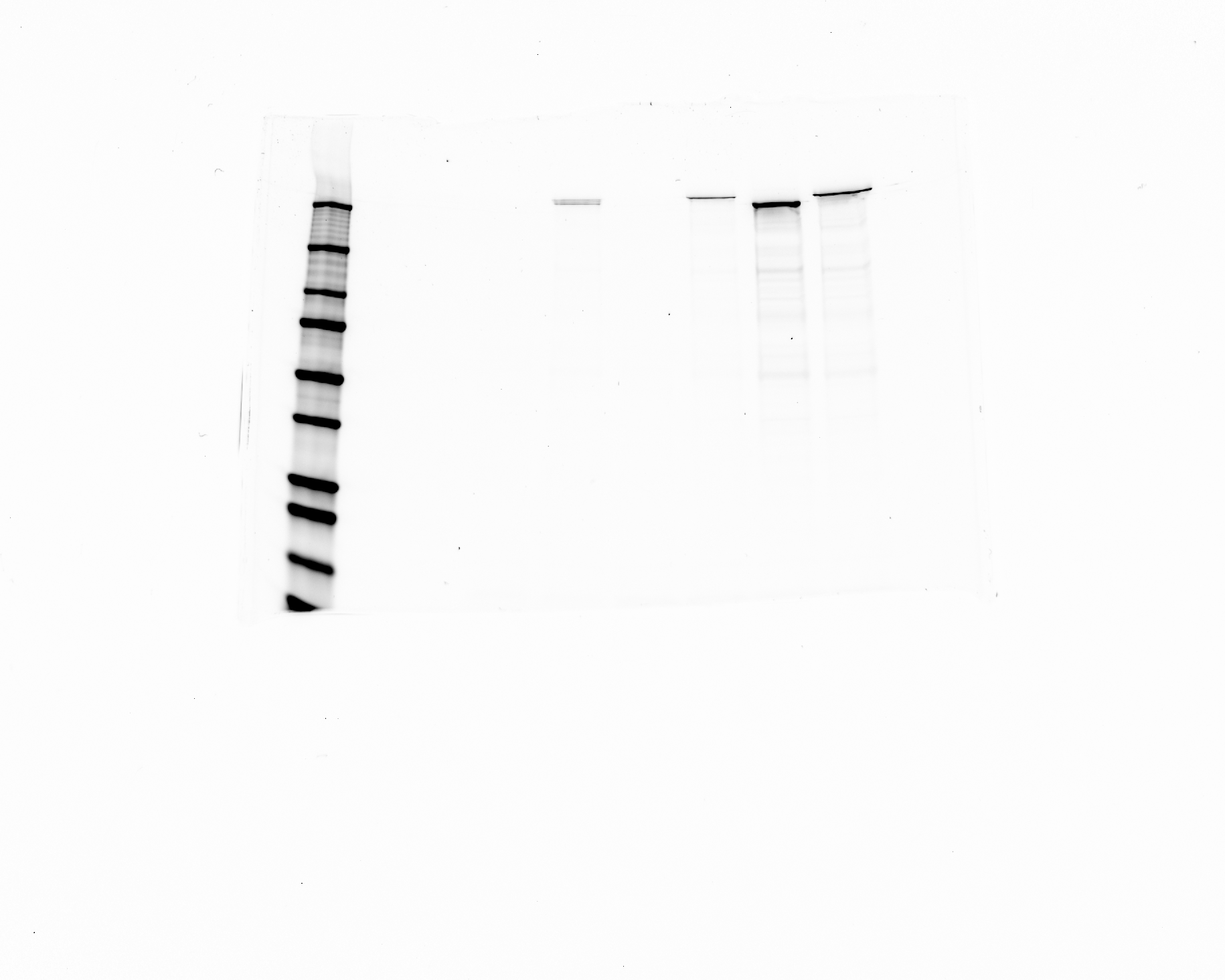

Supplement: Figure 7—source data 1. [file elife-87086-fig7-data1.zip › Figure 7-Source Data 1/Figure 7B/JF646 Fluorescent Gel Expression of HaloTag MDC1 deletion mutants.tif]

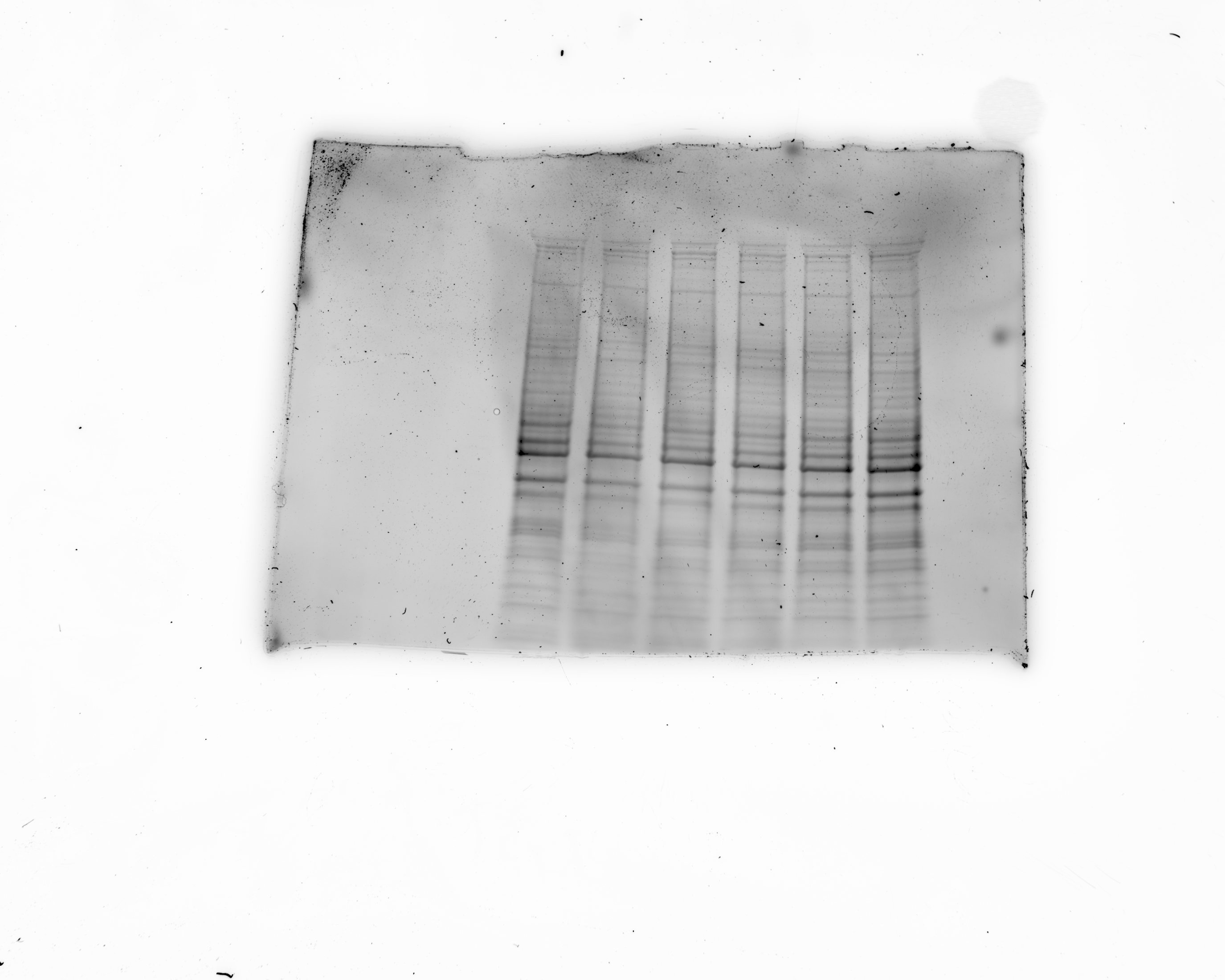

Supplement: Figure 7—source data 1. [file elife-87086-fig7-data1.zip › Figure 7-Source Data 1/Figure 7B/Stain-Free Loading Control.tif]
