# Supplementary material for: Systematic analysis of the molecular and biophysical properties of key DNA damage response factors
Source: eLife. 2023 Jun 21;12:e87086. doi: 10.7554/eLife.87086 (PMC10319438; doi:10.7554/eLife.87086)
Supplement: Figure 1—figure supplement 1—source data 1. [file elife-87086-fig1-figsupp1-data1.zip › Figure 1-Figure Supplement 1-Source Data 1/Figure 1 - figure supplement 1 - source data 1.pdf]

Figure 1-figure supplement 1-source data 1

Figure 1—figure supplement 1C

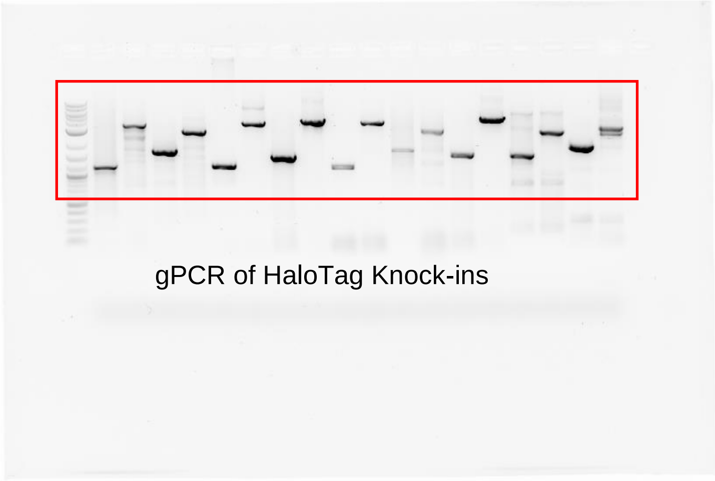

Figure 1—figure supplement 1D

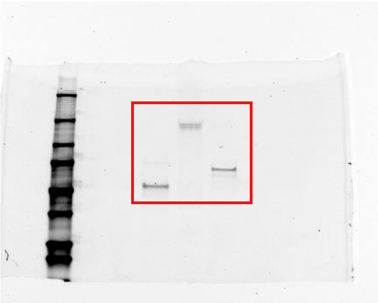

Halo-SHLD1

Halo-SHLD2

Halo-SHLD3

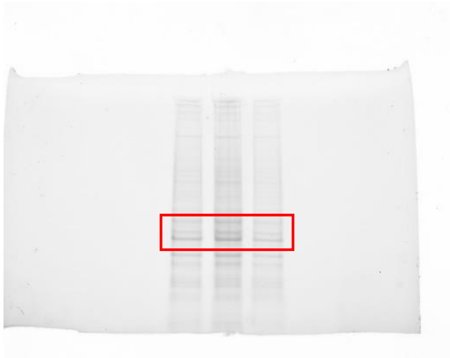

Loading Control

Figure 1—figure supplement 1F

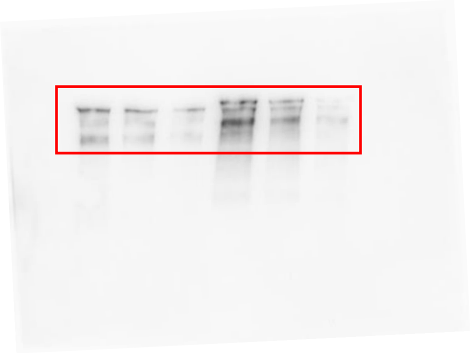

53BP1

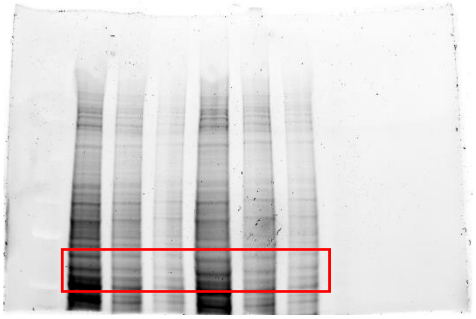

Loading Control

Figure 1-figure supplement 1-source data 1

Figure 1—figure supplement 1F, Continued

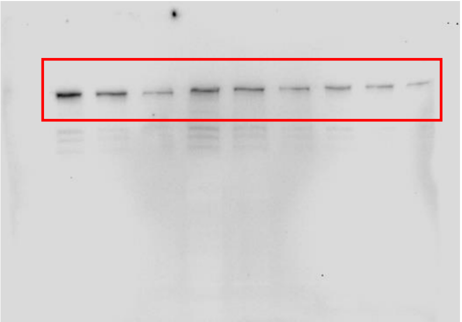

ATM

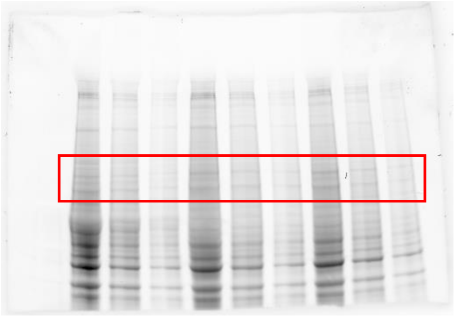

Loading Control

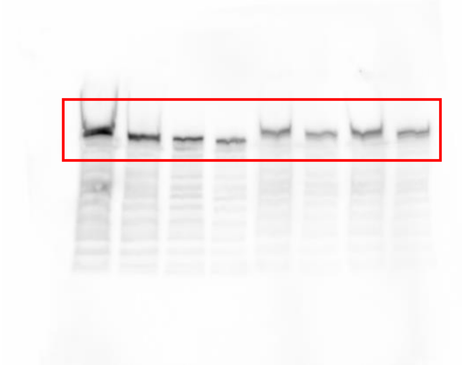

DNA-PKcs

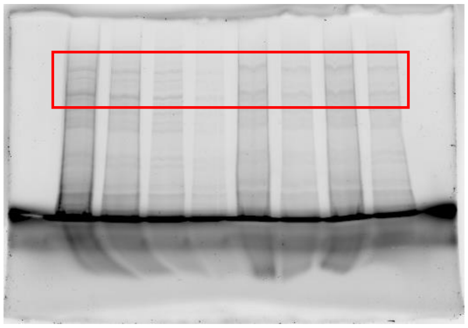

Loading Control

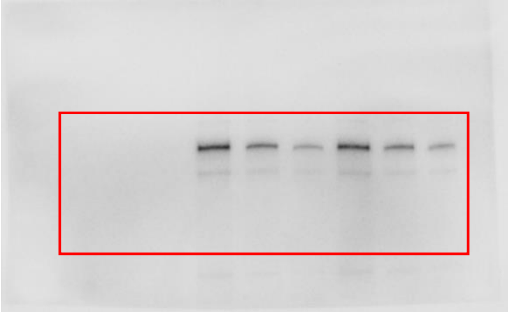

FLAG (Halo-RNF169)

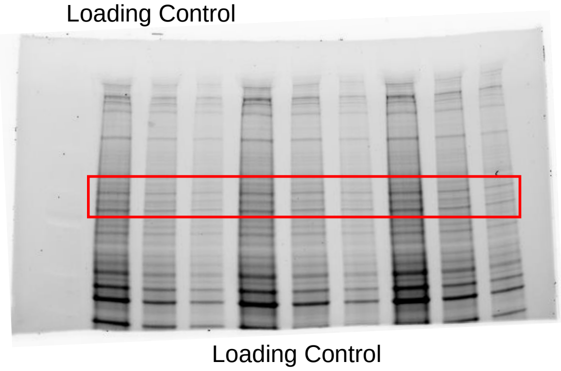

Loading Control

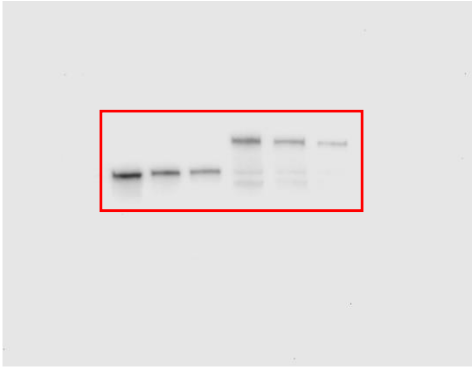

NBS1

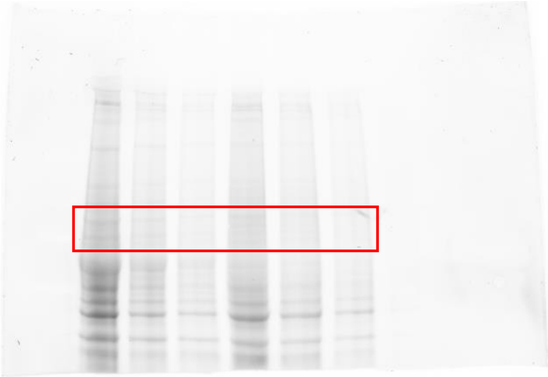

Loading Control

Figure 1-figure supplement 1-source data 1

Figure 1—figure supplement 1F, Continued

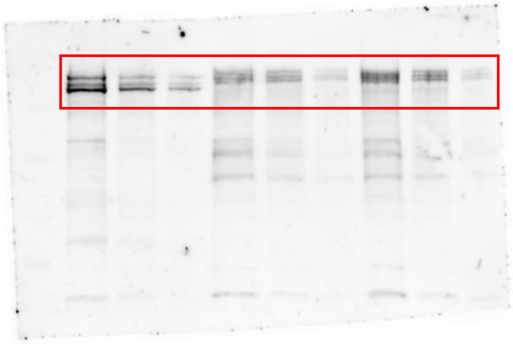

MDC1

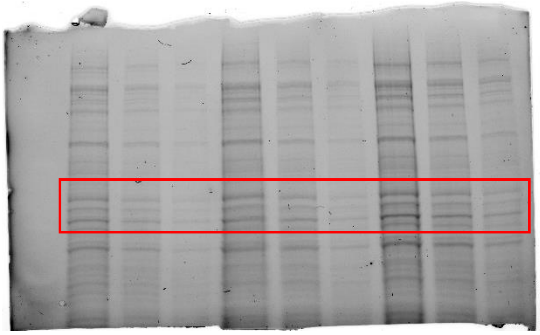

Loading Control

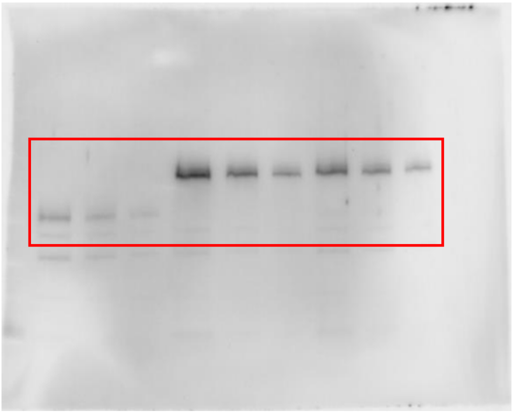

RNF168

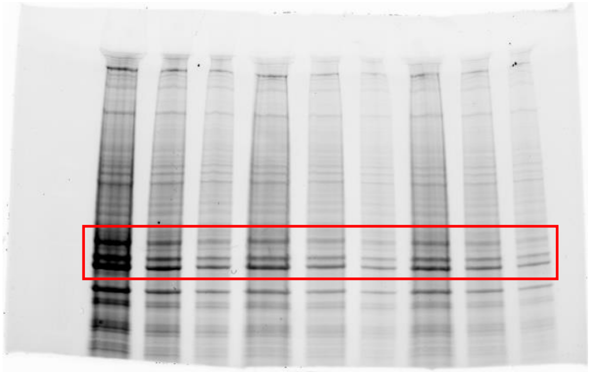

Loading Control

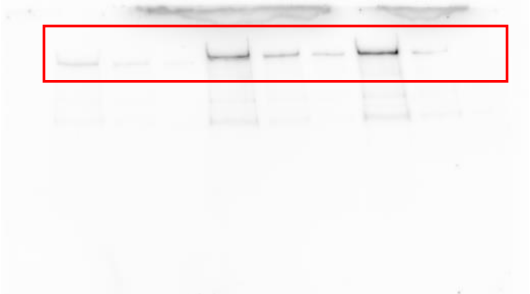

RIF1

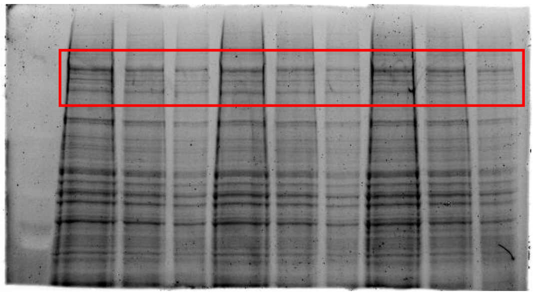

Loading Control

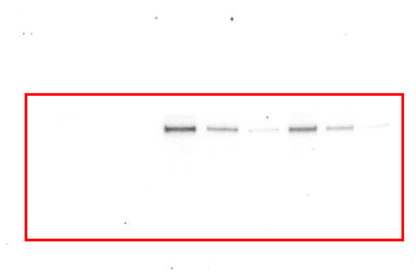

FLAG (Halo-SHLD3)

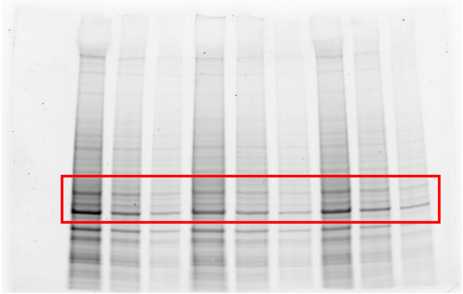

Loading Control

Figure 1-figure supplement 1-source data 1

Figure 1—figure supplement 1F, Continued

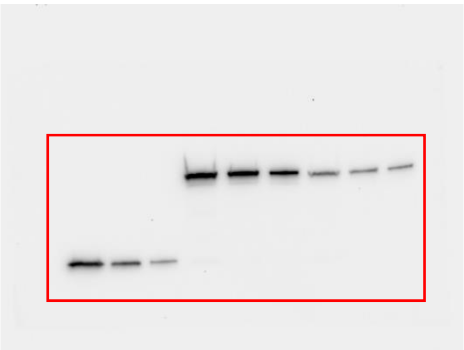

REV7

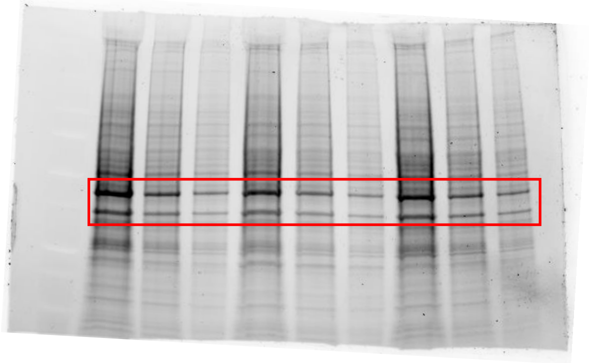

Loading Control

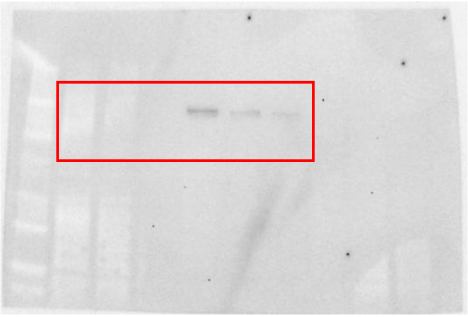

FLAG (Halo-SHLD2)

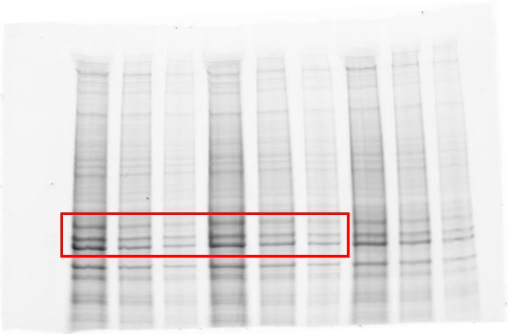

Loading Control

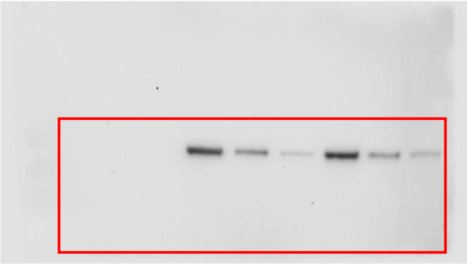

FLAG (Halo-SHLD1)

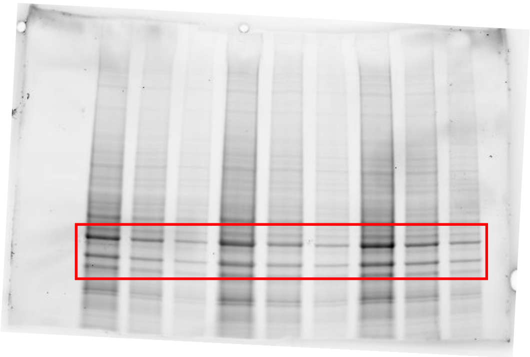

Loading Control
