# Supplementary material for: Systematic analysis of the molecular and biophysical properties of key DNA damage response factors
Source: eLife. 2023 Jun 21;12:e87086. doi: 10.7554/eLife.87086 (PMC10319438; doi:10.7554/eLife.87086)
Supplement: Figure 2—figure supplement 1—source data 1. [file elife-87086-fig2-figsupp1-data1.zip › Figure 2-Figure Supplement 1-Source Data 1/Figure 2 - figure supplement 1 - source data 1.pdf]

Figure 2-figure supplement 1—source data 1

Figure 2—figure supplement 1B

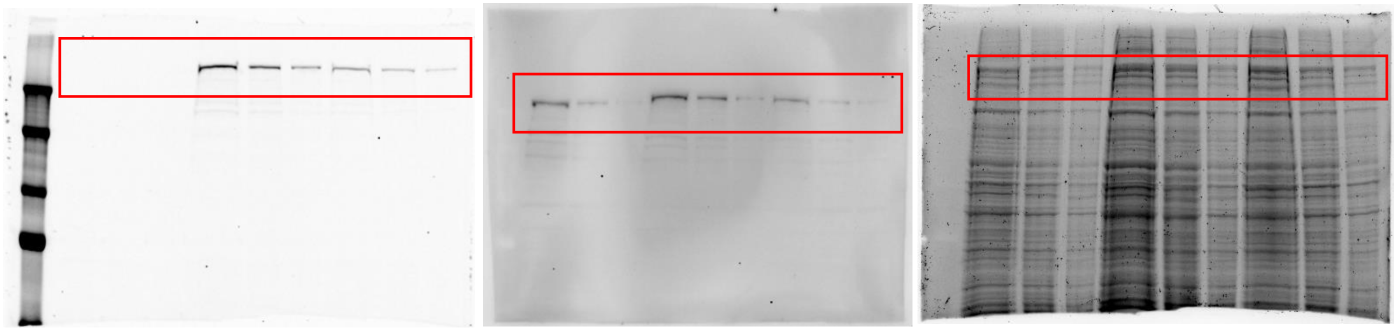

JFX650 (Halo-ATM) ATM Western B Loading Control

Figure 2—figure supplement 1E

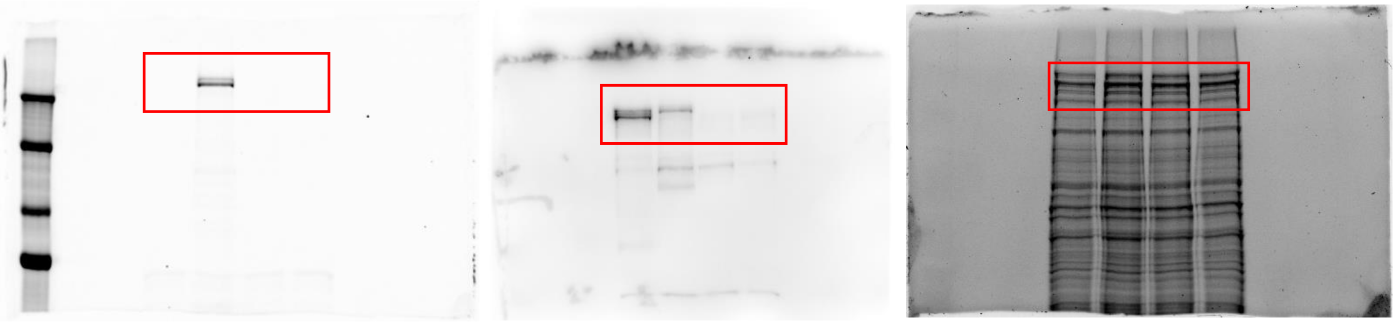

JFX650 (Halo-MDC1) MDC1 Western Blot Loading Control

Figure 2—figure supplement 1F

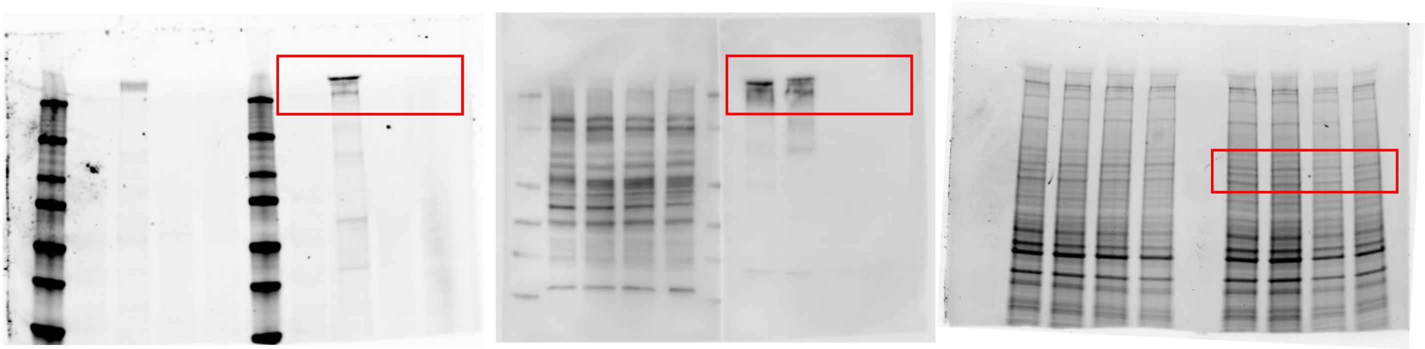

JFX650 (Halo-53BP1) 53BP1 Western Blot Loading Control
