# Supplementary material for: Systematic analysis of the molecular and biophysical properties of key DNA damage response factors
Source: eLife. 2023 Jun 21;12:e87086. doi: 10.7554/eLife.87086 (PMC10319438; doi:10.7554/eLife.87086)
Supplement: Figure 3—source data 1. [file elife-87086-fig3-data1.zip › Figure 3-Source Data 1/Figure 3-Source Data 1.pdf]

Figure 3-source data 1

Figure 3A

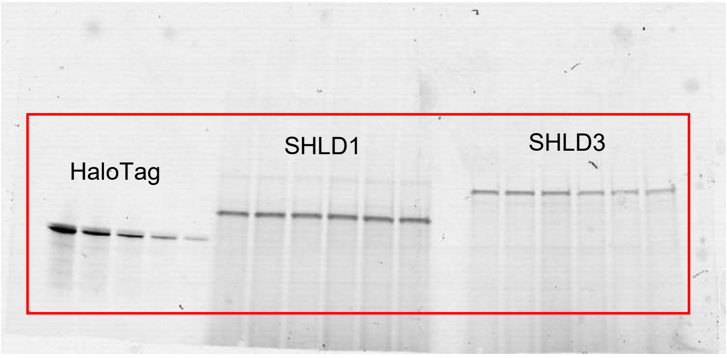

Detection of purified HaloTag, HaloTagged SHLD1, and SHLD3 labeled with JF646

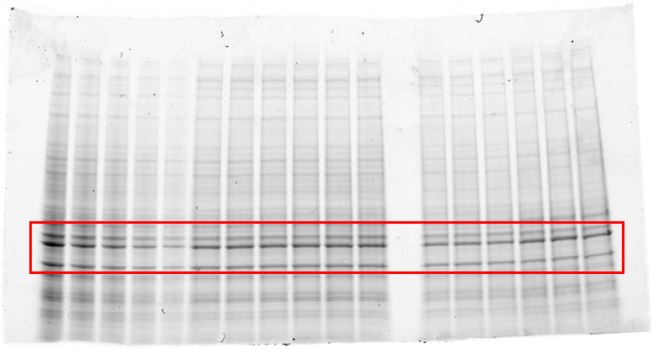

Loading Control
