# Supplementary material for: Systematic analysis of the molecular and biophysical properties of key DNA damage response factors
Source: eLife. 2023 Jun 21;12:e87086. doi: 10.7554/eLife.87086 (PMC10319438; doi:10.7554/eLife.87086)
Supplement: Figure 3—figure supplement 1—source data 1. [file elife-87086-fig3-figsupp1-data1.zip › Figure 3-Figure Supplement 1-Source Data 1/Figure 3 - figure supplement 1 - source data 1.pdf]

Figure 3-figure supplement 1-source data 1

Figure 3—figure supplement 1A

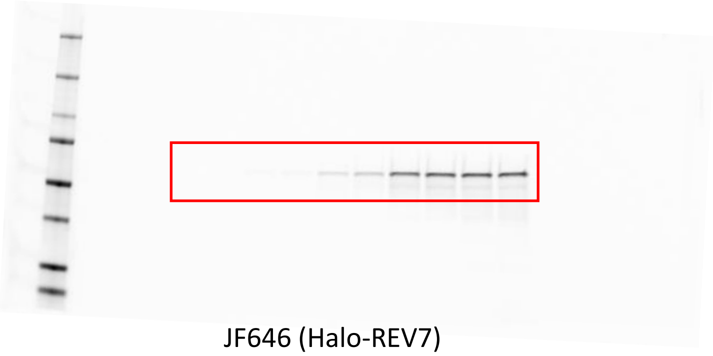

JF646 (Halo-REV7)

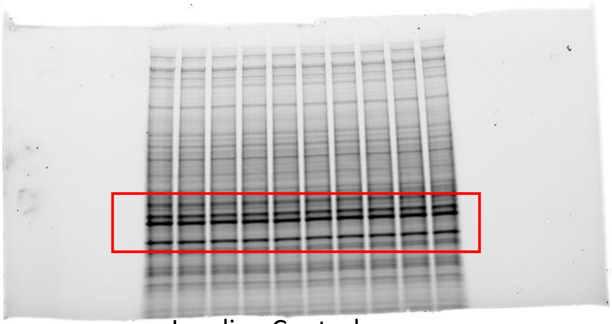

Loading Control

Figure 3—figure supplement 1B

Left Panel:

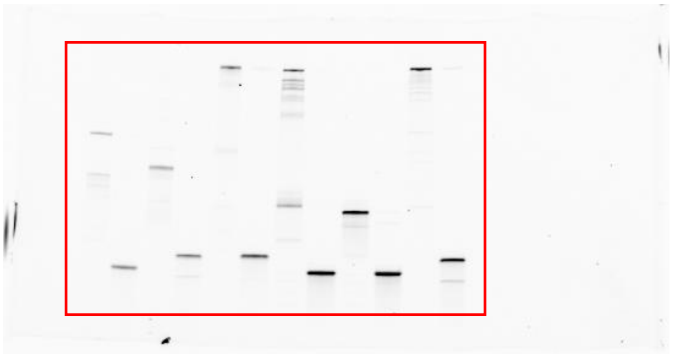

JF646-labeled HaloTagged Proteins

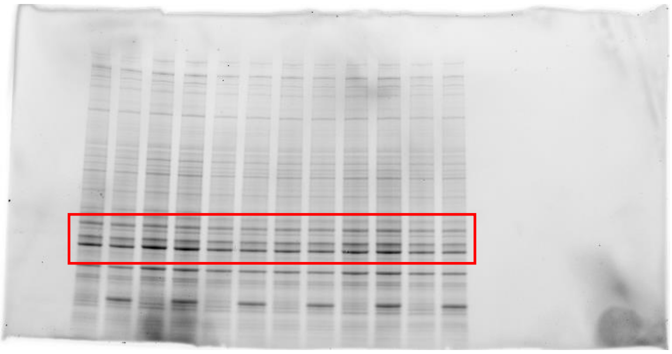

Loading Control

Right Panel:

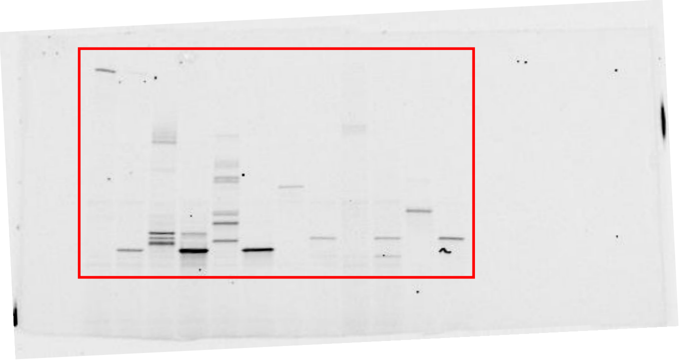

JF646-labeled HaloTagged Proteins

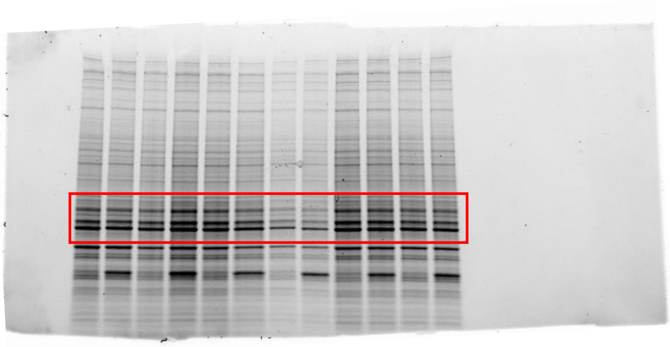

Loading Control

Figure 3—figure supplement 1C

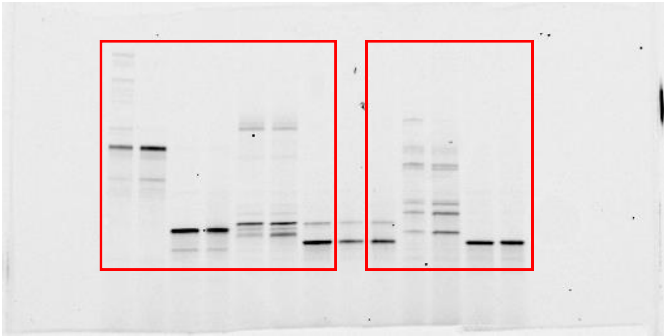

JF646-labeled HaloTagged Proteins

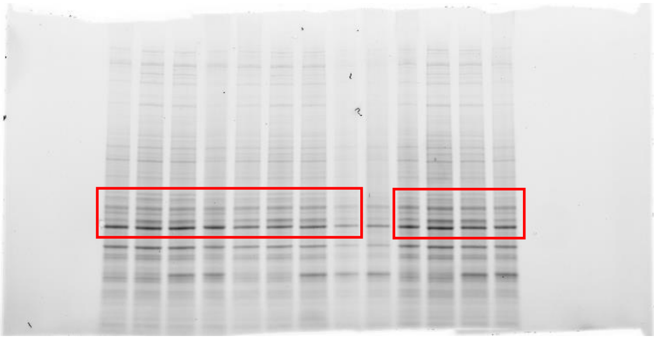

Loading Control
