# Supplementary material for: Systematic analysis of the molecular and biophysical properties of key DNA damage response factors
Source: eLife. 2023 Jun 21;12:e87086. doi: 10.7554/eLife.87086 (PMC10319438; doi:10.7554/eLife.87086)
Supplement: Supplementary file 1. [file elife-87086-supp1.docx]

| **OLIGONUCLEOTIDES** |
| --- |
| **CLONING of HDR DONORS IN pFASTBAC DUAL (5’ – 3’)** |
| **3XFLAG-PURO-HALOTAG FOR N-TERMINAL HALOTAGGING**  ATGGACTACAAAGACCATGACGGTGATTATAAAGATCATGATATCGATTACAAGGATGACGATGACAAGATAACTTCGTATAATGTATGCTATACGAAGTTATAAGCTTCACACAAAAAACCAACACACAGATGTAATGAAAATAAAGATATTTTATTTCAGGCACCGGGCTTGCGGGTCATGCACCAGGTGCGCGGTCCTTCGGGCACCTCGACGTCGGCGGTGACGGTGAAGCCGAGCCGCTCGTAGAAGGGGAGGTTGCGGGGCGCGGAGGTCTCCAGGAAGGCGGGCACCCCGGCGCGCTCGGCCGCCTCCACTCCGGGGAGCACGACGGCGCTGCCCAGACCCTTGCCCTGGTGGTCGGGCGAGACGCCGACGGTGGCCAGGAACCACGCGGGCTCCTTGGGCCGGTGCGGCGCCAGGAGGCCTTCCATCTGTTGCTGCGCGGCCAGCCGGGAACCGCTCAACTCGGCCATGCGCGGGCCGATCTCGGCGAACACCGCCCCCGCTTCGACGCTCTCCGGCGTGGTCCAGACCGCCACCGCGGCGCCGTCGTCCGCGACCCACACCTTGCCGATGTCGAGCCCGACGCGCGTGAGGAAGAGTTCTTGCAGCTCGGTGACCCGCTCGATGTGGCGGTCCGGATCGACGGTGTGGCGCGTGGCGGGGTAGTCGGCGAACGCGGCGGCGAGGGTGCGTACGGCCCTGGGGACGTCGTCGCGGGTGGCGAGGCGCACCGTGGGCTTGTACTCGGTCATGAGCTTTTTGCAAAAGCCTAGGCCTCCAAAAAAGCCTCCTCACTACTTCTGGAATAGCTCAGAGGCCGAGGCGGCCTCGGCCTCTGCATAAATAAAAAAAATTAGTCAGCCATGGGGCGGAGAATGGGCGGAACTGGGCGGAGTTAGGGGCGGGATGGGCGGAGTTAGGGGCGGGACTATGGTTGCTGACTAATTGAGATGCATGCTTTGCATACTTCTGCCTGCTGGGGAGCCTGGGGACTTTCCACACCTGGTTGCTGACTAATTGAGATGCATGCTTTGCATACTTCTGCCTGCTGGGGAGCCTGGGGACTTTCCACACCGGATCCATAACTTCGTATAATGTATGCTATACGAAGTTATCCGGTACCATGGCAGAAATCGGTACTGGCTTTCCATTCGACCCCCATTATGTGGAAGTCCTGGGCGAGCGCATGCACTACGTCGATGTTGGTCCGCGCGATGGCACCCCTGTGCTGTTCCTGCACGGTAACCCGACCTCCTCCTACGTGTGGCGCAACATCATCCCGCATGTTGCACCGACCCATCGCTGCATTGCTCCAGACCTGATCGGTATGGGCAAATCCGACAAACCAGACCTGGGTTATTTCTTCGACGACCACGTCCGCTTCATGGATGCCTTCATCGAAGCCCTGGGTCTGGAAGAGGTCGTCCTGGTCATTCACGACTGGGGCTCCGCTCTGGGTTTCCACTGGGCCAAGCGCAATCCAGAGCGCGTCAAAGGTATTGCATTTATGGAGTTCATCCGCCCTATCCCGACCTGGGACGAATGGCCAGAATTTGCCCGCGAGACCTTCCAGGCCTTCCGCACCACCGACGTCGGCCGCAAGCTGATCATCGATCAGAACGTTTTTATCGAGGGTACGCTGCCGATGGGTGTCGTCCGCCCGCTGACTGAAGTCGAGATGGACCATTACCGCGAGCCGTTCCTGAATCCTGTTGACCGCGAGCCACTGTGGCGCTTCCCAAACGAGCTGCCAATCGCCGGTGAGCCAGCGAACATCGTCGCGCTGGTCGAAGAATACATGGACTGGCTGCACCAGTCCCCTGTCCCGAAGCTGCTGTTCTGGGGCACCCCAGGCGTTCTGATCCCACCGGCCGAAGCCGCTCGCCTGGCCAAAAGCCTGCCTAACTGCAAGGCTGTGGACATCGGCCCGGGTCTGAATCTGCTGCAAGAAGACAACCCGGACCTGATCGGCAGCGAGATCGCGCGCTGGCTGTCGACGCTCGAGATTTCCGGCGAGCCAACCACTGAGGATCTGTACTTTCAGAGCGATAACGCGATCGCTTCC |
| **HALOTAG-3XFLAG-PURO FOR C-TERMINAL HALOTAGGING**  GGAGCTCTCGAGCCAACCACTGAGGATCTGTACTTTCAGAGCGATAACGATGGATCCGAAATCGGTACTGGCTTTCCATTCGACCCCCATTATGTGGAAGTCCTGGGCGAGCGCATGCACTACGTCGATGTTGGTCCGCGCGATGGCACCCCTGTGCTGTTCCTGCACGGTAACCCGACCTCCTCCTACGTGTGGCGCAACATCATCCCGCATGTTGCACCGACCCATCGCTGCATTGCTCCAGACCTGATCGGTATGGGCAAATCCGACAAACCAGACCTGGGTTATTTCTTCGACGACCACGTCCGCTTCATGGATGCCTTCATCGAAGCCCTGGGTCTGGAAGAGGTCGTCCTGGTCATTCACGACTGGGGCTCCGCTCTGGGTTTCCACTGGGCCAAGCGCAATCCAGAGCGCGTCAAAGGTATTGCATTTATGGAGTTCATCCGCCCTATCCCGACCTGGGACGAATGGCCAGAATTTGCCCGCGAGACCTTCCAGGCCTTCCGCACCACCGACGTCGGCCGCAAGCTGATCATCGATCAGAACGTTTTTATCGAGGGTACGCTGCCGATGGGTGTCGTCCGCCCGCTGACTGAAGTCGAGATGGACCATTACCGCGAGCCGTTCCTGAATCCTGTTGACCGCGAGCCACTGTGGCGCTTCCCAAACGAGCTGCCAATCGCCGGTGAGCCAGCGAACATCGTCGCGCTGGTCGAAGAATACATGGACTGGCTGCACCAGTCCCCTGTCCCGAAGCTGCTGTTCTGGGGCACCCCAGGCGTTCTGATCCCACCGGCCGAAGCCGCTCGCCTGGCCAAAAGCCTGCCTAACTGCAAGGCTGTGGACATCGGCCCGGGTCTGAATCTGCTGCAAGAAGACAACCCGGACCTGATCGGCAGCGAGATCGCGCGCTGGCTGTCTACTCTGGAGATTTCCGGTGACTACAAAGACCATGACGGTGATTATAAAGATCATGATATCGATTACAAGGATGACGATGACAAGTAACTCGAGTCTAGACGTTTAAACCCTGCAGGCTGTGCCTTCTAGTTGCCAGCCATCTGTTGTTTGCCCCTCCCCCGTGCCTTCCTTGACCCTGGAAGGTGCCACTCCCACTGTCCTTTCCTAATAAAATGAGGAAATTGCATCGCATTGTCTGAGTAGGTGTCATTCTATTCTGGGGGGTGGGGTGGGGCAGGACAGCAAGGGGGAGGATTGGGAAGACAATAGCAGGCATGCTGGGGATGCGGTGGGCTCTATGGGTCGACAGTACTAAGCTTTACGTGTGTCAGTTAGGGTGTGGAAAGTCCCCAGGCTCCCCAGCAGGCAGAAGTATGCAAAGCATGCATCTCAATTAGTCAGCAACCAGGTGTGGAAAGTCCCCAGGCTCCCCAGCAGGCAGAAGTATGCAAAGCATGCATCTCAATTAGTCAGCAACCATAGTCCCGCCCCTAACTCCGCCCATCCCGCCCCTAACTCCGCCCAGTTCCGCCCATTCTCCGCCCCATGGCTGACTAATTTTTTTTATTTATGCAGAGGCCGAGGCCGCCTCGGCCTCTGAGCTATTCCAGAAGTAGTGAGGAGGCTTTTTTGGAGGCCTAGGCTTTTGCAAAAAGCTTACCATGACCGAGTACAAGCCCACGGTGCGCCTCGCCACCCGCGACGACGTCCCCAGGGCCGTACGCACCCTCGCCGCCGCGTTCGCCGACTACCCCGCCACGCGCCACACCGTCGATCCGGACCGCCACATCGAGCGGGTCACCGAGCTGCAAGAACTCTTCCTCACGCGCGTCGGGCTCGACATCGGCAAGGTGTGGGTCGCGGACGACGGCGCCGCGGTGGCGGTCTGGACCACGCCGGAGAGCGTCGAAGCGGGGGCGGTGTTCGCCGAGATCGGCCCGCGCATGGCCGAGTTGAGCGGTTCCCGGCTGGCCGCGCAGCAACAGATGGAAGGCCTCCTGGCGCCGCACCGGCCCAAGGAGCCCGCGTGGTTCCTGGCCACCGTCGGCGTCTCGCCCGACCACCAGGGCAAGGGTCTGGGCAGCGCCGTCGTGCTCCCCGGAGTGGAGGCGGCCGAGCGCGCCGGGGTGCCCGCCTTCCTGGAGACCTCCGCGCCCCGCAACCTCCCCTTCTACGAGCGGCTCGGCTTCACCGTCACCGCCGACGTCGAGGTGCCCGAAGGACCGCGCACCTGGTGCATGACCCGCAAGCCCGGTGCCTGAAATAAAATATCTTTATTTTCATTACATCTGTGTGTTGGTTTTTTGTGTG |
| **SHLD1 N-TERMINAL HALOTAG LEFT HOMOLOGY ARM (LHA)**  TAAAATGAATGCAATTGTTGTTGTTGGAGTGGGACAGCATGAGATTTCATTACAATACTCAGAATGGTGCACAATTTGAAATTTATGAATTGTTTATTTCTGGAGTTTTCCATGTAATATTTTTGAACCACAGTTGGGTGAGATCAACTGAAACTGCAGAGAGCAAAATCGCACATAAGGGGGGACTACTGTAAATCATTCTGATTTCACTGTTGTTTGACCTTTAAAATTTTTTGGGTGGGTATGGTGGCTCACACCTATAATCCCATCACTTTGGGAGGCTGAGGTGGGAGGATTGCTTGAGCCCAGGAGTTCAAGACCAGCCTGGGCAACTTAGTGAGACCTGATCTCTACAAAGATAAGAATAAAAATAAAAAACAAATAAAATTCTTCAAGCTCTGTCTCCAATGAAGGATCCTGCTATGTGGTGCCTTTTGTACTGAATTGTTTTCTTTTTTCCATGGCAGGACTATGGACTACAAAGACCATGACGGTGATT |
| **SHLD1 N-TERMINAL HALOTAG RIGHT HOMOLOGY ARM (RHA)**  TCAGAGCGATAACGCGATCGCTTCCGCAGCCAGGGACGCCACTTCAGGCAGCCTGTCAGAGGAGAGCAGTGCTTTGGACCTGCCATCAGCGTGTGACATAAGAGATTACGTCCTGCAGGGACCCAGCCAAGAAGCCAACAGCGAGGCTTTCAGTTCTTTGGAATTCCATTCTTTTCCTTATTCTTCTGATGTGGATCCAGGTAATAAGCAGAGTTTAAAACAAACTAACTTAAAAACAACTAGCAGCAATACGGGTGTGTGATAGTTTGTTTCTCTCTCTTCCAATAGCAGATCTCTGAGAATGCTTCATTGTCTTAGGCAGAAAAACATCTTACCTAAGCAAAGCTTTCAGGAGGAACTTCAAAGGACAGCTTACCTGGCACAGCAATGACAAGGAGCTGTTTCCTGGATGTCTTTTTGTTATTGTTGCTGTTAATCTCAGGTCTCTTTCTAATCTCCCTCAAAATGAATTCAAACTTGTTTATTGCAGCTTATAATG |
| **SHLD3 N-TERMINAL HALOTAG LHA**  TAAAATGAATGCAATTGTTGTTGTTGATACTTCCAAGTCCTTTATATCTATTGAGTTAAACATGTTGACTACTAGCGTTTTTAGGCAGGAAGTGATTTTATAGGGCATCTAGTTCAGATCCCTTATTTTGGTAAGAGAAAGAATTTTCAGGTTTGTAGATTACATTCACATTTACCAATAGCACTTTGGTAAAATACCACATTGGGATTGGTTTTTGTTTTTTGTTTTTCCTTTTCTCCCTGCCTGTTGGTAGGCAGGGTATCCCTACCATATCTTATTCTTTGCAATACTTCTACTTTAGGTCCTGTTTCCCTCTGATTTGGCAAGAGAGACAATCTTGCAATAATAAATTAACATTCTAGCTCTGAGGAGTTCAACTAAGAAATTTTCTCATCACTAAGAGTAACTGGATTACTGCAGAATGGACTACAAAGACCATGACGGTGATT |
| **SHLD3 N-TERMINAL HALOTAG RHA**  TCAGAGCGATAACGCGATCGCTTCCACTACAGAAGTAATATTACATTATCGACCATGTGAGAGTGATCCCACACAACTGCCAAAAATTGCAGAAAAAGCAATTCAAGACTTTCCTACTCGTCCGCTATCAAGATTTATACCTTGGTTTCCATATGATGGGTCCAAGCTTCCACTCAGACCTAAAAGATCACCACCTGTGATTTCTGAAGAGGCAGCTGAAGATGTGAAACAGTACTTAACCATTTCAGAACATGATGCTAAGTCACACAGTTATGATTGCACAGTAGATCTATTGGAGTTTCAACCTAGCTTGAAAAAGCAGCATTTAACCTGGTCACACACACTGAAGGAACAGACTAATTCTGGAAATCTGGGTAAACAATCAGAAAAGGGAAAACAGCACAAGAGGAGATCTTGGAGTATTTCCCTTCCCAGCAATAATTGTACTAAAAACGTTTCTCCTTTGTCTAAAAAAACTTGTTTATTGCAGCTTATAATG |
| **SHLD2 N-TERMINAL HALOTAG LHA**  TAAAATGAATGCAATTGTTGTTGTTCTCCGCCTCCTGGGTTCAAGCGATTCTTCTGCCTCAGCCTTCAAGTGGCTGGGACTACAGGCGTGTGCTACCACGCCCAGCTAATTTTTTGTTTTTAGTATAGACGGCGTTTCACGCTGTTAGGCAGGATGGTCTCAATCTCCTGACCTCGTGATCCACCCGCCTCGGCCTCCCAAAGAGCTGGGATTACAGGTGTGAGCCACTGCGCCCGGCCTAAAGTGAGTCTTTAGGTGAAAAATTTTTGTGAATATTGTGATTTCTCCTTAATAATTTCTTGTGTATTTATGAATTTTATATAATTAAATGGCATATCAAAAAAATCACCTATGTTAATTATGTTTTTGACTCAGTTATATTTTCTTTTGGTACATTATTAAGTTAATATCCATATGCAAAAATATGCTGACAATATATTAATTTTGGATTTTTCATTTTTATCAGAAATCATGGACTACAAAGACCATGACGGTGATT |
| **SHLD2 N-TERMINAL HALOTAG RHA** TCAGAGCGATAACGCGATCGCTTCCAGTGGAGGATCTCAAGTCCACATTTTTTGGGGTGCTCCAATTGCTCCACTGAAAATCACAGTATCAGAAGACACAGCTTCTTTAATGTCTGTTGCTGACCCCTGGAAAAAAATTCAGCTTTTATACAGTCAACATTCTTTATATCTGAAGGATGAAAAACAGCACAAAAATCTTGAAAACTATAAAGTCCCAGAATCTATTGGTTCTCCAGATCTTAGTGGTCATTTCTTAGCAAACTGTATGAATAGACATGTTCATGTGAAAGATGACTTTGTACGTTCTGTTTCTGAAACACAGAATATAGAATCCCAGAAGATTCACTCCTCTAGACTGAGTGATATAACTAGCTCTAATATGCAAATATGTGGATTTAAAAGCACAGTTCCGCATTTCACCGAAAACTTGTTTATTGCAGCTTATAATG |
| **REV7 C-TERMINAL HALOTAG LHA** TAAAATGAATGCAATTGTTGTTGTTGGCAGGCCAGGGCCCAGCCCCGTCACCCCCCTGACCTTTGTCTCCTCCCTAGGATTTCCCCTGGATCCTGGCGGATGAGCAGGATGTCCACATGCATGACCCCCGGCTGATACCACTAAAAACCATGACGTCGGACATTTTAAAGGTGAGCTTCGTGGGGACCCGGGAAGCTTTATTTAGGGGCTGCTTAACGAAGTGAGGCCCGGTGCTGAGGCCTTACTAGAGGGCTTGCGGGAGAGAACGGGGGAGTGCCTGGTGGAGGGCTCCTCCCATCTCTACTCTTCTCCACCATGGCTATGGCCACGAGGCCCCACCTGTGGTGGAGGTCTGCCCACCAGGGGCTTCTGTGTCCTCCAGCAGGGATGCCCTGGCTGTCTTGCTGACTGTGGCTGTTTGCTTGTGTCCGTCAGATGCAGCTTTACGTGGAAGAGCGCGCTCATAAAGGCAGCGGAGCTCTCGAGCCAACCACTGAGG |
| **REV7 C-TERMINAL HALOTAG RHA** CATCTGTGTGTTGGTTTTTTGTGTGGGGGGCACCTGCCACCCCACTGATGCCCAAACTGTCAGACTTTGGGGGATCCCCGCCTAGGGCAGTGCTGCATGGCTGCCCTGATTCCAAGTGCTCTTATCGCCTCTGTGTGTGGATCGCCCGCCCCAGCCCGGGGCCGCTCAGGTCTGCTTGGAGGATGCCTCCCCCAGGAGGGCAGTGAGGGATGCCGCAACCTCGACTTCTCAGCCTCCTGGGGTTCCGCCGGCCAACACTGTCTGTCTCAAATACTGTGCTGTGAGTTGTTTCAATAAAGGGGCCCCAAGGGCTGGGCTGAGCTGAGCCATCATTAACTGGAGTGGGAGGGACAGGTATGGCCAGGGTAACCTGGGAGAGGGGCTGCTTCTCCACCAAAATGCCAGGGACCCCCTCTCCCCACTCCTAAAACCAACCCCAGGCTGGTTTTACTGAAAACATTTATTTTTTATTTAAACTTGTTTATTGCAGCTTATAATG |
| **RNF168 C-TERMINAL HALOTAG LHA**  TAAAATGAATGCAATTGTTGTTGTTAGACATAAACAAGAAGAACAGGACAGGTTATTGGCATTACAACTTCAGAAGGAGGTGGATAAAGAGCAAATGGTGCCAAACCGGCAAAAAGGATCCCCAGATGAGTATCACTTACGCGCTACATCCTCCCCTCCAGACAAAGTGCTAAATGGACAGAGGAAGAATCCCAAAGATGGGAACTTCAAAAGGCAAACTCACACAAAGCATCCAACACCAGAGAGAGGCTCAAGGGACAAAAATAGGCAAGTGTCTTTAAAGATGCAGTTGAAGCAGTCAGTTAATAGAAGAAAGATGCCAAATTCTACTAGAGATCACTGTAAGGTATCCAAAAGTGCTCACTCCCTACAGCCTAGCATTTCACAGAAAAGTGTTTTTCAGATGTTTCAGAGATGCACAAAGGGAGCTCTCGAGCCAACCACTGAGG |
| **RNF168 C-TERMINAL HALOTAG RHA** CATCTGTGTGTTGGTTTTTTGTGTGGGCCTGGTAAAGGGAGTGCTTTGTGATCTAGTAAAGCTGGAATGTGAAGCTCTTTCCTATCATGGAATCTTCATCCATGTGTCATTCATGCTGCTCTCTAGGCACACTCATTGTCCTTAATGAAGGTCTGTGTGATTGTAAGGACTGCATAGACACATGAGCTTCTGCACAGCTCAGCGGTAAGCAGGGGTCCCCTGTCGTTTCCCTTCTTAATAACAGATGTGTATTAAGCTTTACAATTCTTAGAGATATCTTAAGCTTTGGCCAACTCCAGGAAATCTTCAAGTGCCATTGTCTTTCCAAAAACTGCATGTTTTATGGCCTTTGCTCACCTTTTTGTCGTGACTGGTAATCAAAGCAAGATTATACTATTCTGAGTGATTAAGGAACCCATGCAGGGTTTAAAATCCATTTTCTTGCTATTGTCCAATGTTTACAGAGTACTCAAAAACTTGTTTATTGCAGCTTATAATG |
| **RIF1 C-TERMINAL HALOTAG LHA** TAAAATGAATGCAATTGTTGTTGTTGAGCTGAGATTACGCCACTGCACTCCAGCCTGGGCAATAGAATGAGGCTCCATCTCATAATAATAATAAGCAGCAGCGGCAGCTCTGGTAGATTTTTTTGTGTGCTTGTCCTCATGAAGACTTAAGCCTGCTCTCTCAATTTGAAACCTAGGCACTTGGACTAACTTAAAAGATCATGTAAAAAATTTAATTTTTGTCTGGTATAGATTTAATTGTGGTTTTTTTTTTCTCTTTTAGATATAATTGATCCTGTTGCTTTAGAAATTCCATTATCCAAAAACCTTCTGGCACAGATTAGTGCTCTTGCTCTTCAGCTGGATTCAGAAGATCTTCATAATTATTCAGGAAGCCAACTATTTGAAATGCACGAGAAACTAAGTTGTATGGCAAACTCTGTAATAAAAAATCTACAGTCACGTTGGAGATCACCATCCCATGAAAATTCTATTGGAGCTCTCGAGCCAACCACTGAGG |
| **RIF1 C-TERMINAL HALOTAG RHA** CATCTGTGTGTTGGTTTTTTGTGTGTATTTTCAGAGAAAATTGAAGGTTTTTTTAAACATCACTGGATTTCTTGATTGAGGAAACAAGTTCTGAAATAATAGCACAATTTCAAAGAAGAGACTCTTTGCAAAGTTGATAACATTTCAAACCCTGAAGGACAGTGACTTATTATGTAAGTTCAATTTTGTAAGTTCATTATGTAAGATCCTTTTTTTTTTCATAATATGTATTCTTGGCTGCTATGCGTGGTTTTTCAGGAAATTTAATTATCTTACTGAGATGTGAAAGCAAAACTAGTAACAGAACTTACATTTTATTTCATGCTTTCTTAAACCCGTGCATATTCTGGTGAAACATGTAAAATACTTTAAGTAAAATTGAACATTTTTATTTGAATTTTTGCTGAACTGATAAAGGTGTTTATATTTTTGTTTGTTGGTTTGTTTAATTCATGTTTGTTGGGACTGAGGTTTAACTTGTTTATTGCAGCTTATAATG |
| **ATM C-TERMINAL HALOTAG RHA** TAAAATGAATGCAATTGTTGTTGTTTAATACATATGTTCTCTCTGTTTAGGTCCTTCTATATGATCCACTCTTTGACTGGACCATGAATCCTTTGAAAGCTTTGTATTTACAGCAGAGGCCGGAAGATGAAACTGAGCTTCACCCTACTCTGAATGCAGATGACCAAGAATGCAAACGAAATCTCAGGTGAGCAGTATTTTAAGAAGGTCCTGTTGTCAGTTTTTCAGATTTTCTTATTCCCAAGGCCTTTAAACTGTTCACCTCACTGAAACCTTTGTGTTTTTGTCCTTAGTGATATTGACCAGAGTTTCAACAAAGTAGCTGAACGTGTCTTAATGAGACTACAAGAGAAACTGAAAGGAGTGGAAGAAGGCACTGTGCTCAGTGTTGGTGGACAAGTGAATTTGCTCATACAGCAGGCCATAGACCCCAAAAATCTCAGCCGACTTTTCCCAGGATGGAAAGCTTGGGTGGGAGCTCTCGAGCCAACCACTGAGG |
| **ATM C-TERMINAL HALOTAG RHA**  CATCTGTGTGTTGGTTTTTTGTGTGTCTTCAGTATATGAATTACCCCTCCTTACTGGCATAACATATTATATTTTAGCCTTTATTTTTAACCTGCCAACATACTTTAAGTAGGGATTAATATTTAAGTGAACTATTGTGGGTTTTTTTGAATGTTGGTTTTAATACTTGATTTAATCACCACTCAAAAATGTTTTGATGGTCTTAAGGAACATCTCTGCTTTCACTCTTTAGAAATAATGGTCATTCGGGCTGGGCGCAGCGGCTCACGCCTGTAATCCCAGCACTTTGGGAGGCCGAGGTGAGCGGATCACAAGGTCAGGAGTTCGAGACCAGCCTGGCCAAGAGACCAGCCTGGCCAGTATGGTGAAACCCTGTCTCTACTAAAAATACAAAAATTAGCCGAGCATGGTGGCGGGCACCTGTAATCCCAGCTACTCGAGAGGCTGAGGCAGGAGAATCTCTTGAACCTGGGAAACTTGTTTATTGCAGCTTATAATG |
| **RNF169 C-TERMINAL HALOTAG LHA** TAAAATGAATGCAATTGTTGTTGTTTAGACAAAACCTGTATAAGCAGAGCCATGAAAATCACCACAGTTAATTCAGTGCTACCCCAAAACAGTGTTTTGGGTGGAGTCCTCAAAACAAAGCAACAATTGAAGACATTAAATCATTTTGATCTGACTAATGGTGTTCTAGTTGAGAGCCTAAGTGAAGAGCCACTTCCTTCTTTGCGTCGAGGCCGGAAAAGACACTGCAAGACCAAGCACTTAGAACAAAATGGCTCCCTTAAAAAACTGCGACAAACCAGTGGGGAGGTGGGTCTGGCCCCAACAGACCCAGTCCTGCGAGAGATGGAGCAGAAGCTTCAGCAAGAGGAAGAAGACCGACAGTTGGCTCTGCAGTTGCAGCGCATGTTCGACAATGAGAGGCGGACTGTGAGCCGGCGAAAAGGAAGTGTGGATCAGTATCTCCTACGGTCCAGCAACATGGCCGGGGCCAAGGGAGCTCTCGAGCCAACCACTGAGG |
| **RNF169 C-TERMINAL HALOTAG RHA** CATCTGTGTGTTGGTTTTTTGTGTGCACCTAATGAAGTGTTACCTATTTTTAAAAGGTCTTAGGCCTTGATCATTTATCCTGAAGAGCTGAGTGTTCTCACTTTGGTTTTATTTTAATGGCAAAACACTGTCTAATATGGTTCTGAGAGGTTCCAGGGCCTTTGTAGTCAATATCCAAGGGAAAAGCATCTCCGTTTCTCTGTGACCCAGGCCAGAAGCCTGAGTGACCCATCCCTAAGGGCTTCTGGGCCAAACCTGGCAGCACCCACTGGGAATGAGATTTGGAACGGCCTCAGGAGCATAATGGCCACAGTTAGTAATGGTAAAGAGGGGATACCTTCTTAAACTGATAGACTTCCTGACTTCTTTCAGCAGGGTATTGTTTTAAATCAGCCTTGCAGATAAAAATTAATTCCATCTTTTTCAGACAAGTGAACAATTTAGTTCCTTGGCAGATCCAAAATGATAGATGGTAACTTGTTTATTGCAGCTTATAATG |
| **NBS1 C-TERMINAL HALOTAG LHA** CATAAAATGAATGCAATTGTTGTTGTTGGCGGATTACGAGGTCAGGAGTTCAACACTAGCCTGGCCAGCATGGAGAAACCCCGTCTCTACTAAAAATACAAAAATTAGCCAGGCGTGGTGGCATGCACCTGTAATCCCAGCTACTCAGGAGGCTGAGGCAGGAGAATCGCTTGAACCCAGGAGGCGGAGGCTGCGTGAGCCAAGATCGCGCCACTGCACTCCAGAGAGAGACTCCATCTCAAAAAAAAAAAAAAAAAAAAGTGTTGACAGACAATACTGGTTTTTCATTAGTCAAATGAGATGGGTTGTTCAGCCATATTTGTTTAACTGCTGTAGGAGAAAATGTTGTAAAGTGCTAATTACATTTAAGTAGATTATGCTCTTTTGTGCAAATGATATGCTTTTATTATGGGGAGGAATGGGGATCTTTGAAGCCATACAGAAAGAAGTGTAAACATACCTTCATTTTTAAACTGTTTTCATTAATTATTTTAAAGCTTTAAAGGATTTTTGTTATTGTTGATTTTTTTTTTGTTTTTACCATATTTTCATCAAAGCAACATCAAAGGGATACATGAAAATAAAAATGTCAGTGCTTAGTATCTGATCCTAAGGACAAAATTGTCATTCCCATCCTATTTGCCAAAGTGTGACTACAGTTTGAAAGTTCTTTACCTTTAAAAGAAATACCATCCCTTATCTAAATATCATTTAACCCCATTTCTTTCTTTGCAGATACAATCCTTATTTAAAAAGGAGAAGAGGAGCTCTCGAGCCAACCAC |
| **NBS1 C-TERMINAL HALOTAG RHA** TATAGCATACATTATACGAAGTTATCTGAGGATTTTAAAAAGAAGCCATGGAAAAACTTCCTAGTAAGCATCTACTTCAGGCCAACAAGGTTATATGAATATATAGTGTATAGAAGCGATTTAAGTTACAATGTTTTATGGCCTAAATTTATTAAATAAAATGCACAAAACTTTGATTCTTTTGTATGTAACAATTGTTTGTTCTGTTTTCAGGCTTTGTCATTGCATCTTTTTTTCATTTTTAAATGTGTTTTGTTTATTAAATAGTTAATATAGTCACAGTTCAAAATTCTAAATGTACGTAAGGTAAAGACTAAAGTCACCCTTCCACCATTGTCCTAGCTACTTGGTTCCCCTCAGAAAAAAATTCATGATACTCATTTCTTATGAATCTTTCCAGGGATTTTTGAGTCCTATTCAAATTCCTATTTTTAAATAATTTCCTACACAAATGATAGCATAACATATGCAGTGTTCTACACCTTGCTTTTTTACTTAGTAGATTAAAAATTATAGGAATATCAATATAATGTTTTTAATATTTTTTCTTTTCCATTATGCTGTAGTCTTACCTAAACTCTGGTGATCCAAACAAAATGGCTTCAGTGGTGCAGATGTCACCTACATGTTATTCTAGTACTAGAAACTGAAGACCATGTGGAGACTTCATCAAACATGGGTTTAGTTTTCACCAGAATGGAAAGACCTGTACCCCTTTTTGGTGGTCTTACTGAGCTGGGTGGGAACTTGTTTATTGCAGCTTATAATG |
| **ATM N-TERMINAL HALOTAG LHA** CATAAAATGAATGCAATTGTTGTTGTTGCCTGGCCAAGAGTGCTTTTAAATAGTTCATTGTCAATGTTAAATCCTTGAGTGCTCATTTCCTTATTTACCTGGCTGTTTTCTATTCATCTTTCATGTCTCAATTTAAATGTCATGTTTCTTTGGTCTCAGAGTAATAAAAAGTAAATATACTTCCCCCATCTCCCGCCCCGCAGAGCCTTCCTCTGTTGCCCAGGCTGGAGTACAGTGGCTCGATCTTGGCTCACTGAAAGCTCTGCCTCCCAGATTCAAGCGATTCTCCTGCCTCAGCCTCCTGAGTAGCTGGGATTACAGGCGCGCGCCACCAAGCCCGGCTAATTTTTGTATTTTTAGTAGAGACAGGGTTTCACCATGTTGGTCAGGCTGGTCGAACTCCTGACCTTGTGATCCTCCCACCTTGGCCTCCCAAAGTGCTGGGATTACAGGCGTGAGCCACTGCGTCCAGTGTAAATTATACTTTTATTTTAATCCTGCTACTACTGCAAGCAAGGCAAACATTTTTGTGTTACAGCATTACTTGTATAGATTTTAAGAAAATCTCATTTTAAATACGGAAATGTTAAGAAAAATTATTGTGCCTTTGACCAGAATGTGCCTCTAATTGTACAGTTAAATCTAACTATAAATACTGCAGTATAAAATAATTATATACACATTTTTTCACACCTCTTTCTCTCTATATATGCATATATACATATACATATATATACCTATATGTATTTTTTTTACAGACAGTGATGTGTGTTCTGAAATTGTGAACCATGGACTACAAAGACCATGAC |
| **ATM N-TERMINAL HALOTAG RHA** CGCGATCGCTTCCGGTACCATGAGTCTAGTACTTAATGATCTGCTTATCTGCTGCCGTCAACTAGAACATGATAGAGCTACAGAACGAAAGGTAGTAAATTACTTAAATTCAATTTTTCCTTGAAATAAGTGTGATTAGTAACCCATTATTATTTCCTTTTTATTTTCAGAAAGAAGTTGAGAAATTTAAGCGCCTGATTCGAGATCCTGAAACAATTAAACATCTAGATCGGCATTCAGATTCCAAACAAGGAAAATATTTGAATTGGGATGCTGTTTTTAGGTATTCTATTCAAATTTATTTTACTGTCTTTATTTTTCTCTTTCATATTTATTTCTGTTGTGATATTACTTTTGTGTGTAAGTCTTAACATTTATCTTTGCTTCCTATATATCATTATGCCTTGCATATGAATTTGGCATTTAATATTTATCCAAAACATAATTTTTAAAGGTTGTTCATATAGAAACTTAAAAATTATAAATTATTTCTTCAATAAAATGTTTTAGACATATCTCACTCAAAATTGAGAGAGAATTTCTTTCATTTTAGTTAGTTCACAAGACTTCAGATTAGAGGAAAAAATTATAATAAATGTTAGGATATTATTTTCACACCTTTAAATATTACTCTGATTATAAGTAAATGCTGTGTGTTAAATTGTAATAAAACAAGTAAAAGGAAGATTGGAATACTTGTGTATGAATTTGGTTAAAAACAAAAAATACCTCGAACAATGAAAAAACACAACTGATTGCTGGACAGGAGTGGAACTTGTTTATTGCAGCTTATAATG |
| **MDC1 N-TERMINAL HALOTAG LHA** GAGGAGAGACATCTGAGTGATTTGGGGCAAGTCTTAACCTTATATATGCTTATGTATGTTTCAAGGAGATCTGAAAATCTTAACCCAGCTATGTTGCAGTTTCTTTATCTGAAGAAATGGAAGATAGTAATACTACTTTACAAGGTTCTTGTGAAGATTAAATGAGTCAGAGTCGATATAAAGTAATTAGAACAGTGCTTGACACATAGGAAGCTTCTATATATGTCATCTTTTATTATCATTTTTTGCAGTTGCATCTGATCAAACTGAGGTGGTCTGGTAAAAGTATAAAAAGCATGGGCTAAAAAAGAAAGAAAAACAACGGGGCGCAGTGGCTCACGCCTGTAATCCCAGCACTTTGGGAGGCCAAGGTGGGCGGATCACCCAAGGTCAAGAGTTCGAGTCCACCCTGGCCAATATGGCAAAACCCCATCTCTTCTAAAAATACAAAAATTAGCCAGGCATGGTGGCAGGTGCCTGTAATCTCAGCTACTTGGGAGGCTGAGGTGAGAGAATTGCTTGAAACCCGGGAGGCAGAGGTTGCCGTGGGCCCAAGATCTTACCACTGCATGCCAGCCTGGGGAGCAGAACGAGACTCCATCACAAAAAAGAAAAAAATGTTTTAAGTGCTTCAGAAGGTTAACAACAGAAAGATTGGTGGAAAGAGAAGCATCAACGTTCATAATGATCCAGTTTACCCCCTAGTTTTCAGTCTGATAACTCTGATGCTGTTTGTGTGAACCAATAATGAGGATAATTGATAATGTGTATCCTTCCCAGATCATGGACTACAAAGACCATGAC |
| **MDC1 N-TERMINAL HALOTAG RHA** CGCGATCGCTTCCGGATCCGAGGACACCCAGGCTATTGACTGGGATGTTGAAGAAGAGGAGGAGACAGAGCAATCCAGTGAATCCTTGAGGTGTAACGTGGAGCCAGTAGGGCGGCTACATATCTTTAGTGGTGCCCATGGACCAGAAAAAGGTCAGAGGGTATTGGATGTTCAAGTATTGATATAGATCTTTTATTTTTGTGGTAAGGTATTTGGAGGGTTGCAAGAAGCTTATGTATGTTTCAATGAGATGTGAATTTTTTTTTTTCATGAATGGAAGAGATGGGGCCGATTGAGTTGATAGTTGCAGACTACTACCCTGGACTCACTGGAAGTGATTTTACTTTGATAAAAGAAAATTAAGAGATTGAAATGAAGCTGGGTGCAGTGACTCACACCTGTAATTCCCGTGCTTTGAGAGGCCAAGGCAGAAGGATCACTTGAGACTAGGAGTTAGAGACCAGCCTGGGTAGCATAGCAAAACCCTGTCTCTACGAAAAATTTAAAAATTAGCCAGGTGTGGTGGTTCATTTCACATCTGCAGTCCCACCTACTTGGGAGGCTGAGCCAGATGATCACTTGAACCCAAGAGTTCAAGATTGCACTGATCTATGATCATGACACTGCATTCTAGCCTGGGTGACAGAATGAGACCCTGTCTCAAAAAAAAAAAAAAAATCAAATAAAATCCAATTGTGTGCTCTGGAGATGGATGGTAGTGATGATTGCAC |
| **53BP1 N-TERMINAL HALOTAG LHA**  CATAAAATGAATGCAATTGTTGTTGTTCAGCGAATGGACGATCCGATATTGTGTGACGTGACGGGAAAGGGGGAGTTCGCGGCCGGTGGCGGCGGTGGCGACAGCGGCGACCTAGGGATCGATCTGGAGGGACTTGGGGAGCGTGCAGAGACCTCTAGCTCGAGCGCGAGGGACCTCCCGCCGGGATGCCTGGTACTGTTTGGAGAGAAATGGGAGTGCAGTGGGCTCTGAAGGCCTGGATTTCGGGTCTGAAGGGATGGCTGACCGGAGGGGGCGGGGTGGCAAGCATGGCGGCGGTGACGGGGAAGGGGTTGGAGGGCCCTAAGGAGGGGGTGGGGAAACGTTAGCTAAGAAGGCTTGGTGGTCGTGAGGAAAGCTGAAAGGCTGTGGGGCCTCTGGCGAACAGATTTTAGAAAACATTCTTTTGGGGGGAGGTAGAGGCGCCAGAAGGACGTGGCGGGGGGGATGGAAAAAGGGGCGGGGAGAGAAGAGGTTAGGAAGGAGGGGAAAGGGTGTCTACGAGAAGGTGATGAAAAATAGGGAGGGATAGTCAGGCTTATTGTAAATAACGAGGAATGGTGACTTGGTAGGCATGATAGGAAAGTGCTTGCAGCAATTATAAATATAATATTCTAATTGGAGGAGTCCTGAAGTGGTCCCGTTTCCCAGCTCTTGTACTTCCGCCCCATTATTTAGGTTTAGACTGCGTGAGCAAGGCTAGTAGGTTACACGGAACTGATCTTTTGTTATTCCATTCCAGGGGAGCAGATGGACAAAGACCATGAC |
| **53BP1 N-TERMINAL HALOTAG RHA**  CGCGATCGCTTCCGGTACCATGGACCCTACTGGAAGTCAGTTGGATTCAGATTTCTCTCAGCAAGATACTCCTTGCCTGATAATTGAAGATTCTCAGCCTGAAAGCCAGGTTCTAGAGGATGATTCTGGTTCTCACTTCAGTATGCTATCTCGACACCTTCCTAATCTCCAGACGCACAAAGAAAATCCTGTGTTGGTGAGTGATACCTTGTTTGAAGAAGATTGAGCAGATTTTTTTTTTTAAACCGAAAACCAGCAAACATAAGTATTTAGAAGATCATTACCTTCTCAATAAAGTTGATTGGAAAAATTGTTCACAAATAGTAGGTTTCATTTTGAACTATTTCATATATAATGGGGATATTTTGTATGTGTATTTTCTAGGATGTTGTGTCCAATCCTGAACAAACAGCTGGAGAAGAACGAGGAGACGGTAATAGTGGGTTCAATGAACATTTGAAAGAAAACAAGGTTGCAGGTGAGGTGTTTAAAGCTATCTGTCCCCTTTGCATTTTTTGGGTGCTCTTGTTGGTGCTGGGTGGATTTAAACTTTATGTGGTTGTGTCGAATGTATGTAAGGAAGCCTACTGTGGTATCTGCGTAGTAGTCACTCAAAAGTTGATGTGCTAGTGTCACTGATTAGATTGGTATTGTCTGTATTCCTGATTTTGTATTAAGGTTTGTTGGTAAATGTCTTTCATGCTAATATCCAGTTATTTTTTTCAGACCCTGTGGATTCTTCTAACTTGGACACATGTGGTTCCATCAGTCAACTTGTTTATTGCAGCTTATAATG |
| **DNA-PKCS N-TERMINAL HALOTAG LHA** CATAAAATGAATGCAATTGTTGTTGTTGGGGGGGACAGTGCCGCTTCTTTCAGCGCCCGTGCGTAAACCAGAAGTAGGCCTCGCTCGGCCCCCCTTGGTCCAGATGGTCTGACCCCCTGCGCGGCAGCGTTTTCGCGGGAAAACTGGGTCCTACAGCACCAGCTCTCGGGGTGTGTTCCTCTAGCGTTGGCTAGGGAGCGTGTGCTTCTTTATCCCTTAGGCGTTTTTGGTTAATAAGGACATTACAGATGCATTTCTCACTTAAAGATTTTTTTTATTAAAAAAAATTAAGGACGGGAGGTGGTGGGGATGGTCTCATTATGTTGCCCAGACTGGTCTCGAACTCCTGCGATCCCCCCGCCTCAGCTTCCTAAAGTGTTGGGACTGCAGGCGGGCATCACCGCCCCCGACCGCGTTTGTCGTTTTTTATCGAGGAACAAACTTGGAACTCTTGACCTAGGCCCCTCGCTTGTTTTATCTGCCTCTGGTATTTATTTAGCCAAGTCCAACACCAAGTAGCCACCCAAACTACCTCCGCAGGTCAGACGTTTTTCCTTAGGTTTCCATGTTGATTCGGGCCAAAGAGGCGCGCTTACTGGCCAGGCCTTCCCGCAGGGGTCCCCGGGAAAGTTCCTGCCGCCGCGCCCCGCAGCCCCGCCTCCGCGCGTAGGGGCATTTCCGGGTCCGGGCCGAGCGGGCGCACGCGCGGGAGCGGGACTCGGCGGCATGGACTACAAAGACCATGAC |
| **DNA-PKCS N-TERMINAL HALOTAG RHA** CGCGATCGCTTCCGGTACCATGGCGGGCTCCGGAGCCGGTGTGCGTTGCTCCCTGCTGCGGCTGCAGGAGACCTTGTCCGCTGCGGACCGCTGCGGTGCTGCCCTGGCCGGTCATCAACTGATCCGCGGCCTGGGGCAGGAATGCGTCCTGAGCAGCAGCCCCGCGGTGCTGGGTGGGTACCGGCCCGAGCTGGGCCGCGGGTGGGTCCTGGCTTCCCGAGTCGTTCTGGAGCCGGGCAGCCCGGCGCTTCTCTGTGTTTAGATGACGGTATTTTGAAGTACAGCAATTAGATTCTTGGGGGAATTTCTGTGTATGTGTATAAAGGGATTTGTCATACTGCTTTTTACCCCCTAGTATTAGAGGAAAGATATTTTAAAGCCAAAGTTGGTGAAAACGACCCCTAATATGGCAATCATTGCTATGCAGAGTGGGGGGCAGGGCAAGAGAAATAATTTTTTTTTTTTTTGAGACGGAGTCTCGCTCTGTCGCCCAGGCTGGAGTGAAGTGGCGCGATCTCGGCTCACTGCAACCTCCGCCTCCCGGGTTCAAGCTATTCTCCTGCCTCAGCCCCCCGAGTAGCTGGGATTACAGGCGCGCGCCACCACGCCCGGCTAATTTTTTTGTATTTTTAGTAGAGATGGGGTTTCACCATGTTGGTCAGGCTGGTCTCGAACTCCTGACCTCGTGATCCCCCCGCCAACTTGTTTATTGCAGCTTATAATG |
| **gRNA SEQUENCES USED FOR KNOCK-IN/KNOCKOUT (5’ – 3’)** |
| **ATM N-TERMINAL HALOTAG KNOCK-IN**  ATCATTAAGTACTAGACTCA |
| **MDC1 N-TERMINAL HALOTAG KNOCK-IN**  GTATCCTTCCCAGATCATGG |
| **53BP1 N-TERMINAL HALOTAG KNOCK-IN**  GGGGAGCAGATGGACCCTAC |
| **NBS1 C-TERMINAL HALOTAG KNOCK-IN**  TAAAAAGGAGAAGATAACTG |
| **DNA-PKCS N-TERMINAL HALOTAG KNOCK-IN**  AGCGGGACTCGGCGGCATGG |
| **SHLD3 N-TERMINAL HALOTAG KNOCK-IN**  TTACTGCAGAATGACTACAG |
| **REV7 C-TERMINAL HALOTAG KNOCK-IN**  GCTCATAAAGGCAGCTGAGG |
| **RIF1 C-TERMINAL HALOTAG KNOCK-IN**  AATACTAAATAGAATTTTCA |
| **ATM C-TERMINAL HALOTAG KNOCK-IN**  TTTCTAAAGGCTGAATGAAA |
| **SHLD2 N-TERMINAL HALOTAG KNOCK-IN**  TTTTTATCAGAAATCATGAG |
| **RNF169 C-TERMINAL HALOTAG KNOCK-IN**  ACACTTCATTAGGTGCTACT |
| **RNF168 C-TERMINAL HALOTAG KNOCK-IN**  GAGATGCACAAAGTAAGGCC |
| **SHLD1 N-TERMINAL HALOTAG KNOCK-IN**  ATGGCAGGACTATGGCAGCC |
| **HALOTAG KNOCKOUT**  GTCGATGTTGGTCCGCGCGA |
| **MDC1 KNOCKOUT**  GGTGTAACGTGGAGCCAGTA |
| **53BP1 KNOCKOUT**  AGATTCTCAGCCTGAAAGCC |
| **SHLD3 KNOCKOUT gRNA #1**  CGCTATCAAGATTTATACCT |
| **SHLD3 KNOCKOUT gRNA #2**  CTGAAGGAACAGACTAATTC |
| **PCR PRIMERS (5’ – 3’)** |
| **N-TERMINAL 3XFLAG-PURO-HALOTAG FW**  ATGGACTACAAAGACCATGACGGTG |
| **N-TERMINAL 3XFLAG-HALOTAG RV**  GGTACCGGAAGCGATCGCG |
| **C-TERMINAL HALOTAG-3XFLAG-PURO FW**  GGAGCTCTCGAGCCAACCAC |
| **C-TERMINAL HALOTAG-3XFLAG-PURO RV**  CACACAAAAAACCAACACACAGATG |
| **GENOMIC PCR PRIMERS (5’ – 3’)** |
| ***MDC1* FORWARD**  GGAGGGTCTCGCTAAGGAGTTTAG |
| ***MDC1* REVERSE**  CCCCATCCATCCACAATGGATG |
| **NBS1 FORWARD**  GTAAAAATGTTGACAAACAGGCCGG |
| **NBS1 REVERSE**  GACATTCATTGTTTCTTACCATCTCCC |
| **ATM (N-TERMINAL KNOCK-IN) FORWARD**  GAGTAGCTGGGACTACAAGAGCA |
| **ATM (N-TERMINAL KNOCK-IN) REVERSE**  TTTGCCACTCCTGTCCAGC |
| **SHLD3 FORWARD**  GCGATTCTCCTCCCTCAGC |
| **SHLD3 REVERSE**  GGCCTAAATGCCTCTGAATTGTAGC |
| **53BP1 FORWARD**  GATACCGTCATTCTCCGGCTC |
| **53BP1 REVERSE**  CCTTGGGATGAGGCAACAGG |
| **SHLD2 FORWARD**  GTTTGATGTGAGTGTGCTTAGCT |
| **SHLD2 REVERSE**  TGGCACACACTCTCTTTGTACCA |
| **SHLD1 FORWARD**  GCATATCTGACTTGCTGGCATCAC |
| **SHLD1 REVERSE**  GGGCCTCCAGACTCAGATCA |
| **REV7 FORWARD**  TGCCTCTCCAGGCTGTACC |
| **REV7 REVERSE**  AGCCTGGGGTTGGTTTTAGGAG |
| **RNF168 FORWARD**  GTCGTGCCTACTGATCAGTAA |
| **RNF168 REVERSE**  CTGGGATTTCCCACATATGAAT |
| **RIF1 FORWARD**  GCAGTGGTAGGTTGGGTATGGT |
| **RIF1 REVERSE**  ACTTCCTAAACCTCAGTCCCAACA |
| **ATM (C-TERMINAL KNOCK-IN) FORWARD**  TCCAGTCAGGAAACATGGGTCA |
| **ATM (C-TERMINAL KNOCK-IN) REVERSE**  GATGGAGTTTCGCTCTTGTCACC |
| **RNF169 FORWARD**  GACCCACCTCTGCTGATCTTGATC |
| **RNF169 REVERSE**  GCACTATCAGTGTTCAATGCCCAG |
| **DNA-PKCS FORWARD**  AGTCCACCTGGCGAGTAGCG |
| **DNA-PKCS REVERSE**  AGGTACTGGTGGGATTAGGCGAG |
